# How a Single 5 eV Electron Can Induce Double-Strand Breaks in DNA: A Time-Dependent Density Functional Theory Study

**DOI:** 10.1021/acs.jpcb.3c08367

**Published:** 2024-04-23

**Authors:** Anil Kumar, Michael D. Sevilla, Leon Sanche

**Affiliations:** † Department of Chemistry, Oakland University, Rochester, Michigan 48309, United States; ‡ Department of Nuclear Medicine and Radiobiology and Clinical Research Center, Faculty of Medicine and Health Sciences, Université de Sherbrooke, Sherbrooke, QC J1H 5N4, Canada

## Abstract

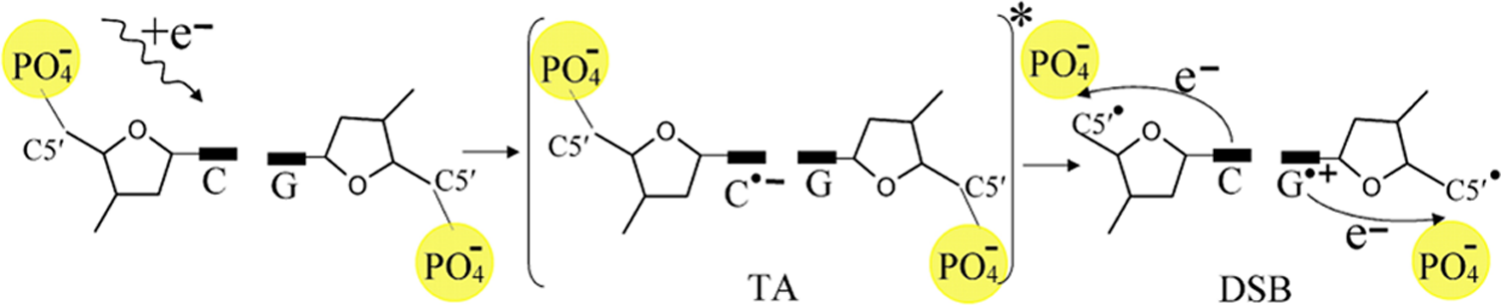

Low-energy (<20 eV) electrons (LEEs) can resonantly interact with DNA to form transient anions (TAs) of fundamental units, inducing single-strand breaks (SSBs), and cluster damage, such as double-strand breaks (DSBs). Shape resonances, which arise from electron capture in a previously unfilled orbital, can induce only a SSB, whereas a single core-excited resonance (i.e., two electrons in excited orbitals of the field of a hole) has been shown experimentally to cause cluster lesions. Herein, we show from time-dependent density functional theory (TDDFT) that a core-excited resonance can produce a DSB, i.e., a single 5 eV electron can induce two close lesions in DNA. We considered the nucleotide with the G–C base pair (ds[5′-G-3′]) as a model for electron localization in the DNA double helix and calculated the potential energy surfaces (PESs) of excited states of the ground-state TA of ds[5′-G-3′], which correspond to shape and core-excited resonances. The calculations show that shape TAs start at ca. 1 eV, while core-excited TAs occur only above 4 eV. The energy profile of each excited state and the corresponding PES are obtained by simultaneously stretching both C5′–O5′ bonds of ds[5′-G-3′]. From the nature of the PES, we find two dissociative (σ*) states localized on the PO_4_ groups at the C5′ sites of ds[5′-G-3′]. The first σ* state at 1 eV is due to a shape resonance, while the second σ* state is induced by a core-excited resonance at 5.4 eV. As the bond of the latter state stretches and arrives close to the dissociation limit, the added electron on C transfers to C5′ phosphate, thus demonstrating the possibility of producing a DSB with only one electron of ca. 5 eV.

## Introduction

Radiation-induced ionization and excitation of biomolecules represent the initial steps in radiation damage induced in cells.^1−11^ The most detrimental damages arise from the ionization of DNA and surrounding molecules that initiate the production of “holes” and secondary electrons (SEs). SEs possess most of the kinetic energy of the initial radiation and can therefore inflict considerable damage to DNA. As SEs lose their kinetic energy in the irradiated medium, they eventually thermalize and attach to DNA bases favoring those of highest electron affinities, i.e., thymine and cytosine.^12^ Most SEs are initially produced as low-energy electrons (LEEs) with energies below 20 eV.^13,14^ LEEs are created in large numbers (ca. 4 × 10^4^ electrons per MeV energy deposited),^15^ along the tracks of high-energy ionizing radiation. For over 2 decades, LEEs have been recognized as contributors to various types of DNA lesions, including single-strand breaks (SSBs), cross-links, base damage and release, double-strand breaks (DSBs), and other clustered lesions.^15,16^ These experiments showed that LEEs having energies below the ionization threshold of DNA were able to cause SSBs and DSBs. The yields of all measured lesions were found to depend on the energy of the interacting LEE and were proposed to be primarily caused by electron resonances and the rapid fragmentation of the resulting transient anions (TAs). These TAs were localized on fundamental DNA components, i.e., the bases, sugar, and phosphate backbone.^15,16^ Additionally, LEEs have been found to cause a variety of damage in DNA model compounds.^17−26^

To aid in understanding these experiments,^15−26^ a number of theoretical efforts have been reported for SSB formation and base release, which have helped to unravel the underlying mechanisms.^27−36^ Mainly, three types of mechanisms of LEE-induced SSB formation in DNA were proposed: (i) Using the Hartree–Fock level of theory, Simons and co-workers^27^ proposed a mechanism for SSB formation in which an excess electron primarily attaches to the π* molecular orbital (MO) of the DNA base (shape resonance) and subsequently transfers to the C–O bond region joining the sugar phosphate group, causing the bond to dissociate. Using the B3LYP/DZP++ level of theory, Bao et al.^29^ also supported Simons’s^27^ proposal of SSB formation. (ii) The second mechanism of SSB formation was proposed by Li et al.^28^ using the B3LYP/6-31+G(d) level of theory embedded in the ONIOM approach. In their model, they considered a sugar–phosphate–sugar (S–P–S) model in which an excess electron interacts directly with the sugar phosphate backbone, initiating the SSB process. (iii) In addition, using the BH and HLYP/6-31G* level of theory, we calculated the potential energy surfaces (PESs) of the C5′–O5′ bond cleavage for the excited states of the 5′-dTMPH radical anion in their gas-phase vertical state (mimicking TA formation) and proposed that LEE < 1.5 eV can access the dissociative phosphate (PO_4_) group causing the SSB.^34^ Recently, Kopyra^37^ reported the attachment of LEEs to entire gas-phase nucleotides (2′-deoxycytidine 5′-monophosphate) and showed that the additional electron resided mainly at the phosphate (60%) with sugar and base following with a lower abundance.

From the above discussion, it is evident that most of these theoretical^27−35^ efforts are limited to the comprehension of SSBs and base damage and release, resulting from the addition of an electron in a previously unfilled orbital of a ground state of a subunit or a small constituent of the DNA molecule. With minimum computational complexities, these studies^27−35^ well described the interaction of subexcitation (<1 eV) electrons with DNA model compounds. However, above the subexcitation electron energy (>4 eV), understanding the mechanism leading to bond rupture must include excitation into shape resonances and excitations at higher energies (>4 eV), which are even more difficult to treat theoretically since they involve the formation of core-excited resonances (i.e., two-electron one-hole states). These latter states consist of electrons localized on multiple electronically excited molecular orbitals in the field of a hole.^11,38^ Thus, theoretical studies on the formation of core-excited resonances in DNA systems have been quite limited.^38,39^ Using the equation-of-motion coupled cluster for electron affinities (EOM-EA-CCSD) and the complete active space self-consistent field (CASSCF), Fennimore and Matsika^39^ calculated the core-excited state of uracil. They proposed that core-excited resonances lying >4.6 eV are expected to play an important role in the dissociative electron attachment (DEA) of uracil. In agreement with the previous work by Kumar and Sevilla,^34^ we show via time-dependent density functional theory (TDDFT) calculations of a deoxyadenosine diphosphate transient anion (TA) radical that core excitations begin at >4 eV. Experiments by Sanche and co-workers^41−43^ demonstrated that a LEE < 20 eV can induce SSBs (1–4 eV) and DSBs (4–12 eV) in plasmid DNA. The yields of DSBs vs electron energy and yield functions of other cluster lesions^41−43^ exhibit two strong maxima around 6 and 10 eV, which were interpreted to result from the formation of core-excited resonances.

**Scheme 1:**
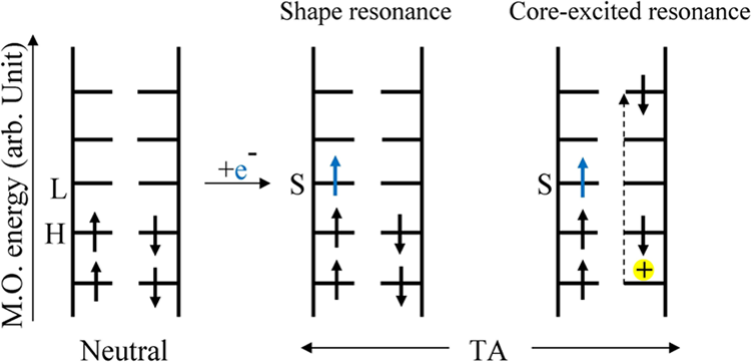
Schematic Diagram Showing the Electronic Configuration of a Neutral Molecule on the Left and Its Transient Negative Ions (TAs) An excess electron is initially captured into an unoccupied molecular orbital (MO) of the neutral molecule, resulting in TA formation via a shape resonance (middle) or a core-excited resonance (right). In a shape resonance, the electron can temporarily occupy any previously unoccupied MO, but only those orbitals that retain the electron for sufficient time and lead to bond dissociation in the Frank–Condon region can cause damage and undergo DEA. In a core-excited resonance, the incident electron is captured by the electron affinity of an electronically excited state. The transition takes place from an occupied orbital to the unfilled MOs, creating a “hole” (+ charge) in the inner shell, shown by a dashed vertical arrow. With the additional electron, this overall configuration is called a 2-electron 1-hole state. The core-excited TA thus consists of two electrons occupying a previously unoccupied orbital and a hole. The shorter up and down arrows show the occupancy of the MOs with electrons of α and β spins. H is highest occupied molecular orbital (HOMO); L is the lowest unoccupied molecular orbital (LUMO); and S denotes a singly occupied molecular orbital (SOMO).^7^

From experiments, it is evident that shape and core-excited resonances (see Scheme 1) are playing crucial roles in producing SSBs and DSBs. Though the mechanism of the SSB below 4 eV is well understood, the mechanism leading to SSBs and DSBs above this energy has not been addressed theoretically. Thus, an important question remains, “how can a single electron of 5–6 eV break two DNA bonds, i.e., induce a DSB?” In this context, a detailed knowledge of excited electronic orbitals, characterizing shape and core-excited states of TAs of DNA is required. In this present work, the calculation of the excited states is based on the concept that LEE resonances forming TAs are equivalent the vertical electronic excited states of the electron adduct of the parent ground state (GS) of the targeted subunit. Since such resonances and core excitations can lie in the continuum, the choice of the basis set is important in these calculations. Thus, to avoid the mixing of valence bound states with dipole bound states and the continuum, we use the compact basis set 6-31G* in the present calculation.^43^ Studies of TAs and determination of the negative electron affinities of DNA bases with the 6-31G* basis set are well documented in the literature,^43−47^ including in our own work.^34,40,48^

**Figure 1:**
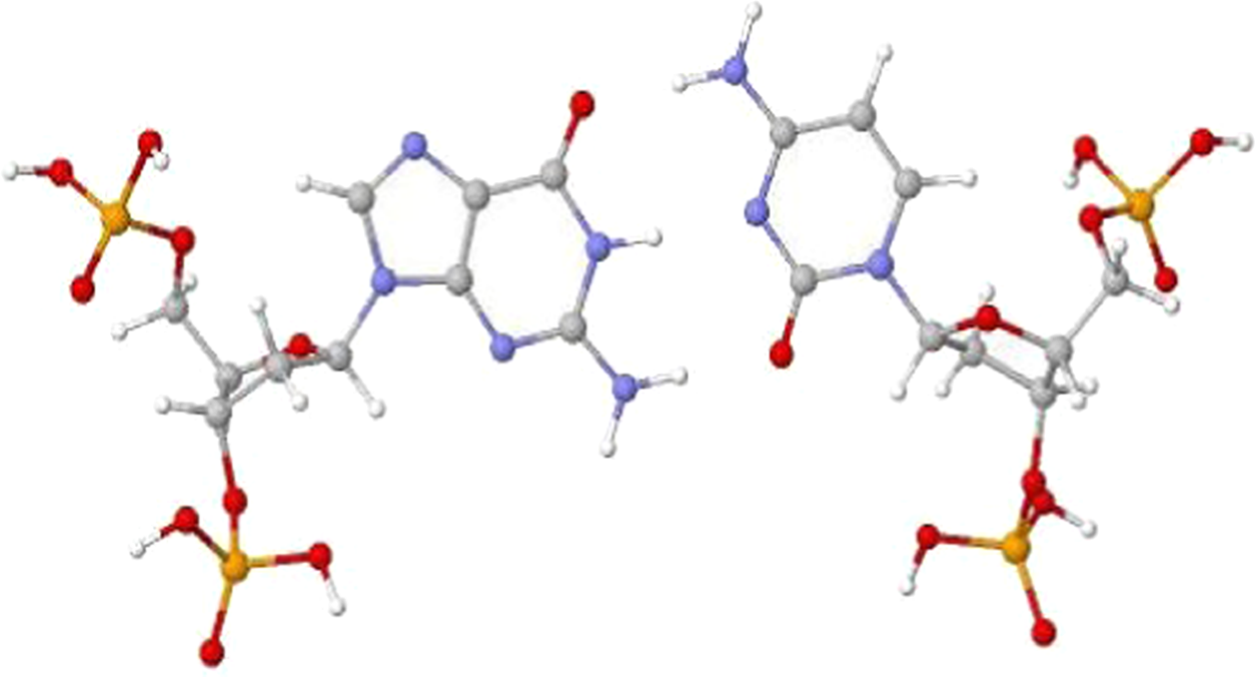
Structure of ds[5′-G-3′]. The PO_4_ groups are attached at 3′- and 5′-ends of the deoxyribose (sugar) ring on both G and C bases. The color codes for atoms are hydrogen (white), nitrogen (blue), oxygen (red), and phosphorus (yellow).

Herein, we consider the double-stranded G–C base pair (ds[5′-G-3′]) as a model for electron attachment in the DNA double helix and calculate the excited states of the TAs of ds[5′-G-3′]. The transition energies were calculated in the gas phase by using time-dependent density functional theory (TDDFT). We calculate the excited states to characterize the shape and core-excited resonances and identify the states leading to DSBs in the chosen ds[5′-G-3′] system. The aim is to provide a mechanism leading to the spontaneous formation of a DSB caused by the attachment of a single LEE, considering that the core-excited TA lives a sufficiently long time to allow for bond dissociation. Our calculations show that the involvement of two dissociative σ* states, arising from a shape and the associated core-excited state, can lead to a DSB. The dissociative nature of σ* electronically excited states is well documented in the literature.^34,48−54^ In ds[5′-G-3′], PO_4_ groups are attached at 3′- and 5′-ends of the deoxyribose (sugar) ring on both G and C bases, as shown in Figure 1.

## Methods of Calculation

Many density functionals reported in the literature^55−58^ have been employed in the calculation of excitation energies. We chose CAM-B3LYP for the present studies because benchmarked studies of the performance of CAM-B3LYP vs other functionals and methods show that for excitation energies, CAM-B3LYP has among the lowest errors vs experiment.^55−58^ CAM-B3LYP prevents errors in charge-transfer excitations, and it provides a low-cost method to excellent results. We note that these benchmark studies were for stable systems, and in this work, we are treating transient anions. Thus, after choosing the CAM-B3LYP functional with a 6-31G* basis set for the present calculations, we also checked the reliability and accuracy of our chosen methodology for the prediction of shape resonance energies of DNA/RNA bases. Electron transmission spectroscopy (ETS)^46^ has been used to find the shape resonance energies of the lowest three π* shape resonances of DNA/RNA bases. The CAM-B3LYP/6-31G* calculated transition energies along with the other theoretical calculations^34,39^ are compared to experimental values for shape resonance values, as shown in Table 1. We note that the shape resonance energies in the ETS experiments correspond to the TA energies. The differences in these TA energies give good estimates of the excitation energies. In this manner, we can compare calculated transition energies to the experimental shape resonance energies. From Table 1, it is evident that CAM-B3LYP/6-31G* calculated values for shape resonances of bases are in good agreement with experimental values within ca. 0.5 eV. For uracil, the CAM-B3LYP/6-31G* calculated shape resonance values are in better agreement than EOM-EA-CCSD/aug-cc-pVDZ+1s,1p,1d calculated values, as shown in Table 1. This clearly demonstrates that our chosen methodology (CAM-B3LYP/6-31G*) is quite suitable for our current work.

**Table 1:** Comparison of Vertical Excitation Energies (Δ*E*, eV) of the Transient Anion (TA) of DNA/RNA Bases Calculated Using TD-CAM-B3LYP Methods with Available Experimental Values of Gas-Phase Shape Resonances

		Δ*E* (eV)				
		CAM-B3LYP	B3LYP^a^	BH&HLYP^a^	EOM-EA-CCSD^b^	
		basis set				
transition	molecule	6-31G* c^,^^d^	6-31G* c^,^^d^	6-31G* c^,^^d^	aug-cc-pVDZ+1s,1p,1d	exp.^e^^,^^f^
	uracil	0.22	0.22	0.22	0.22 (0.65)	0.22 (π_1_*)
(π_1_* → π_2_*)		1.87 (1.65)^d^	1.55 (1.33)^d^	2.07 (1.85)^d^	1.81 (2.24)	1.58 (π_2_*)
(π_1_* → π_3_*)		4.72 (4.50)^d^	4.49 (4.27)^d^	4.95 (4.73)^d^	4.51 (4.94)	3.83 (π_3_*)
	thymine	0.29	0.29	0.29		0.29 (π_1_*)
(π_1_* → π_2_*)		1.98 (1.69)^d^	1.67 (1.38)^d^	2.18 (1.89)^d^		1.71 (π_2_*)
(π_1_* → π_3_*)		4.50 (4.31)^d^	4.15 (3.86)^d^	4.75 (4.46)^d^		4.05 (π_3_*)
	cytosine	0.32	0.32	0.32		0.32 (π_1_*)
(π_1_* → π_2_*)		2.04 (1.72)^d^	1.87 (1.55)^d^	2.23 (1.91)^d^		1.53 (π_2_*)
(π_1_* → π_3_*)		5.09 (4.77)^d^	4.79 (4.47)^d^	5.38 (5.06)^d^		4.50 (π_3_*)
	adenine	0.54	0.54	0.54		0.54 (π_1_*)
(π_1_* → π_2_*)		1.42 (0.88)^d^	1.42 (0.88)^d^	1.54 (1.0)^d^		1.36 (π_2_*)
(π_1_* → π_3_*)		2.37 (1.83)^d^	2.43 (1.89)^d^	2.40 (1.86)^d^		2.17 (π_3_*)

a Ref (34).

b Ref (39).

c Transition energies of radical anions were calculated at the optimized neutral geometry of the molecules.

d Calculated transition energies are compared to shape resonance energies by addition of the lowest shape resonance energy, *E*(π_1_*), to the calculated transition energies, i.e., Δ*E* (eV) *+ E*(π_1_*). The calculated transition energies are given in parentheses for the first column. All transition energies are obtained by simply subtracting the first value, *E*(π_1_*), from the other tabulated values for that base.

e Energies of the shape resonances in the electron transmission spectroscopy (ETS) experiment (ref (46)).

f π_1_* corresponds to the resonance energy of low-energy electron addition to the LUMO, forming the transient singly occupied molecular orbital (SOMO). The difference of the π_1_* from the π_2_* and π_3_* shape resonance energies gives an estimate of the π_1_* → π_2_* and π_3_* transition energies which we calculated.

We have also calculated excitation energies of bases given in Table 1 using a diffuse basis set (6-31++G**) and the calculations are not shown as they fail owing to the formation of dipole bound states instead of valence bound states. Only compact basis sets as used herein are able to properly treat transient anion states.

**Figure 2:**
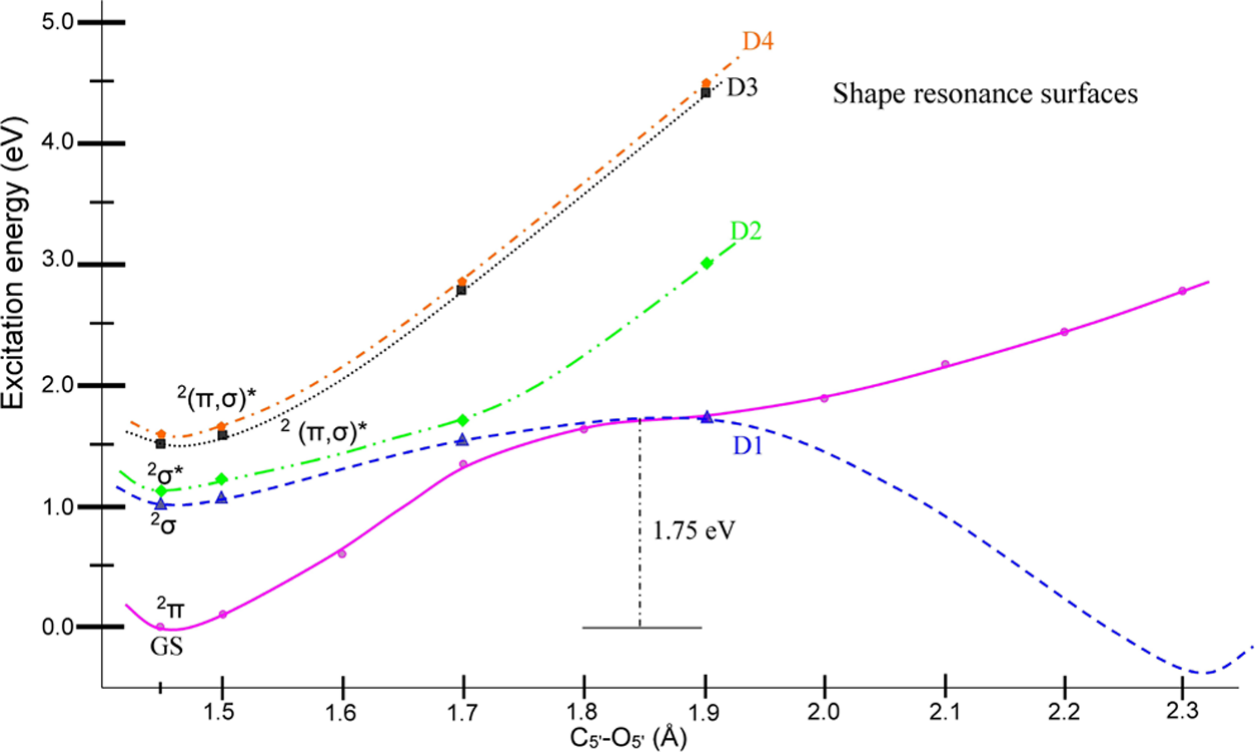
TD-CAM-B3LYP/6-31G* calculated ground-state (lower curve) and vertical excited-state (upper curves) PESs of the ds[5′-G-3′] transient negative ion (TA) along the simultaneous C5′–O5′ bond elongations. The transitions (S1–S4) are taking place from the SOMO (MO number 211) localized on cytosine to higher UMOs. Energies and distances are given in eV and Å, respectively. π and σ designate the nature of each surface on the PES, while GS and asterisk (*) designate the ground-state and excited-state surfaces, respectively.

**Figure 3:**
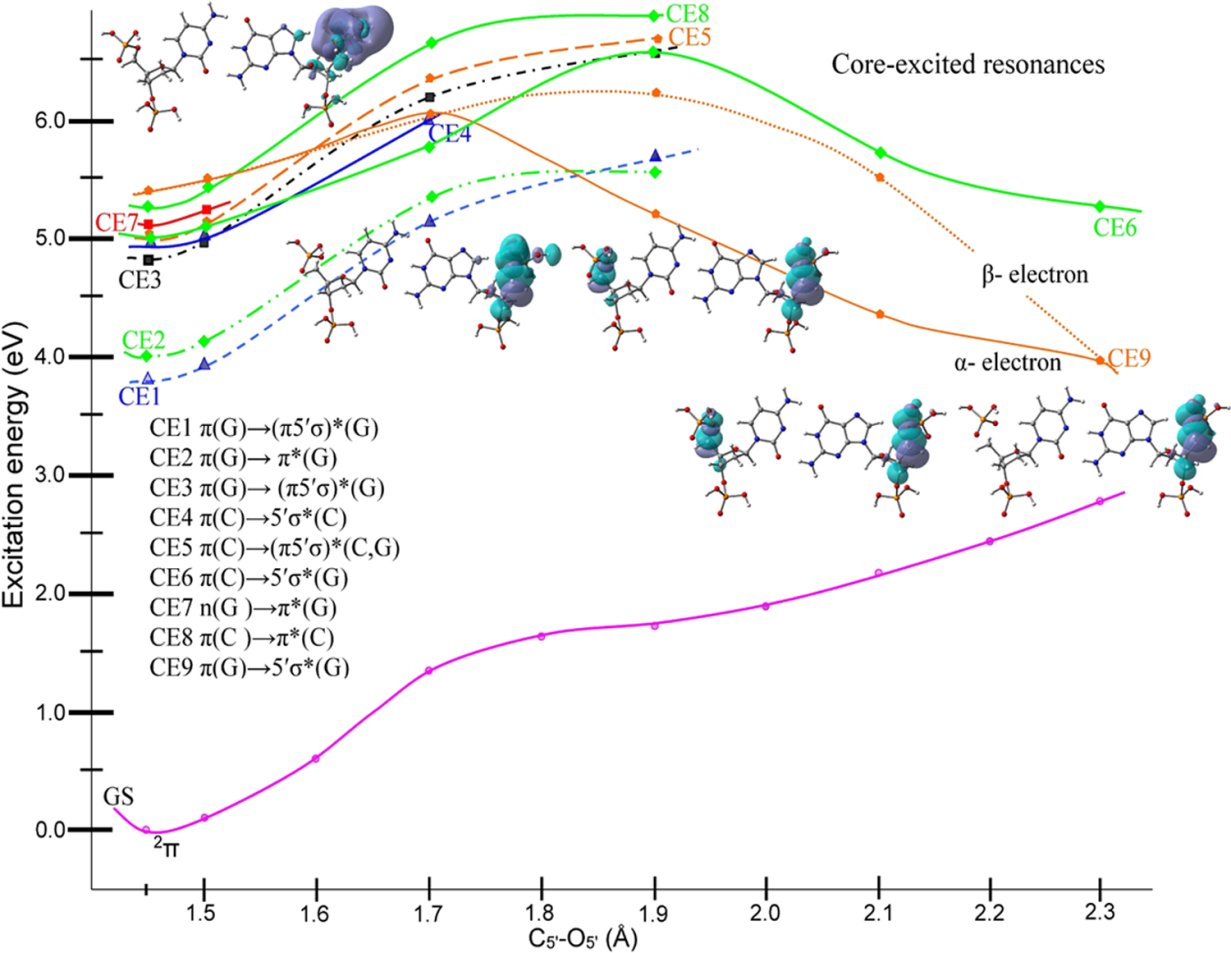
Lowest nine core-excited resonance PESs of the TA of ds[5′-G-3′], calculated by the TD-CAM-B3LYP/6-31G* level of theory, in the neutral optimized geometry of ds[5′-G-3′] with C5′–O5′ bond elongation. Energies and distances are given in eV and Å, respectively. Lowest curve: ground state (GS) of the ^2^π-type. Upper curves: CE1–CE9 core-excited resonances. CE9 upper state molecular orbital configurations are shown during elongation from 1.5 to 2.3 Å. The ninth core-excited state (CE9) is the only clearly dissociative state (CE6 is less so). For CE9, we find that the excitation of the α-electron gives a different PE surface from the excitation of the β-electron in the lower state MO. However, CE9 starts and ends at the same energy for both α and β excitations. Further information on the nature of each core-excited surface (CE1–CE9) is provided in Figure S3-2–10.

To explore the mechanism of DNA strand breaks, a knowledge of the PES along the C5′–O5′ bond stretch in ds[5′-G-3′] is of utmost importance.^34,48^ Thus, we scanned the PES by simultaneously stretching the C5′–O5′ bond (Figure 1) from the equilibrium bond length (1.44 Å) of the ground-state anion radical of ds[5′-G-3′] in the neutral geometry (vertical surface, Figures 2 and 3) to 2.3 Å in steps of 0.1 Å using the CAM-B3LYP/6-31G* level of theory. At each fixed C5′–O5′ bond length along each PES, vertical excitation energies were calculated using the unrestricted time-dependent TD-CAM-B3LYP/6-31G* method.

For the first 100 transitions calculated at the equilibrium geometry of the ds[5′-G-3′] temporary radical anion (i.e., the TA), careful examination of the most involved molecular orbitals allows the identification of the 4 lowest shape resonances (D1–D4) and 12 lowest core-excited states (CE1–CE12), as presented in Table 2. In calculating D1–D4 and CE1–CE12 transitions, we used the following criteria: (i) for shape resonances (D1–D4), only transitions involving the lowest energy SOMO (singly occupied molecular orbital) to higher energy UMOs (unoccupied MOs) of ds[5′-G-3′] TA were considered and (ii) for core-excited transitions (CE1–CE12), dominant MO configurations below SOMOs were considered. After calculating these transitions (D1–D4 and CE1–CE12), we identified the similarly corresponding vertical transitions at each fixed C5′–O5′ bond distance to map the nature of the PES of the ds[5′-G-3′] TA. From Table 2, we see that dissociative (σ*) states at 5′-sites of the sugar moiety attached to G and C have lower transition energies than the σ* at 3′-sites; thus, we considered C5′–O5′ bond elongation to generate the PES of the TA of ds[5′-G-3′]. The shape and core-excited PESs, including the transition energies and associated MOs, are presented in the Supporting Information (SI) 1–9. The ground-state neutral geometry of ds[5′-G-3′] was optimized by the M062X/6-31G* method. The anionic PO_4_ groups were protonated and C5′- and C3′-ends were terminated by hydrogens (Figure 1). The calculations were carried out using the Gaussian 09 suite of programs.^59^ The MO contributions involved in a transition are calculated using the GaussSum program.^60^ To draw the structure and MOs involved in the transitions, we used JMOL^61^ and GaussView^62^ molecular modeling software.

**Table 2:** TD-CAM-B3LYP/6-31G* Calculated Vertical Transition Energies (Δ*E*) in eV and Oscillator Strength (*f*) of the TA of ds[5′-G-3′] in the Gas Phase

state	transition^a^	Δ*E* (eV)	λ (nm)	*f*	contribution^b^ (%)
Shape Resonance^c^					
D1	π(C) → 5′σ*(C)	0.99	1249	0.0146	95
D2	π(C) → 5′σ*(G)	1.11	1120	0.0001	98
D3	π(C) → (π5′σ)*(G)	1.50	824	0.0113	80
D4	π(C) → (π3′σ)*(C,G)	1.56	796	0.0035	89
Core-Excited Resonance^d^					
CE1	π(G) → (π5′σ)*(G)	3.83	324	0.0000	82
CE2	π(G) → π*(G)	4.03	307	0.0000	88
CE3	π(G) → (π5′σ)*(G)	4.89	254	0.0000	55
CE4	π(C) → 5′σ*(C)	4.95	251	0.0243	74
CE5	π(C) → (π5′σ)*(C,G)	5.06	246	0.0072	79
CE6	π(C) → 5′σ*(G)	5.06	245	0.0048	86
CE7	n(G) → π*(G)	5.13	242	0.0000	40
CE8	π(C) → π*(C)	5.32	233	0.3693	21
CE9	π(G) → 5′σ*(G)	5.42	229	0.1407	25–99
CE10	π(C) → (π5′σ)*(C,G)	5.47	227	0.0101	62
CE11	π(C) → 3′σ*(G)	5.51	225	0.0004	81
CE12	π(C) → 3′σ*(C)	5.58	222	0.0047	84

a C and G in brackets are guanine and cytosine bases. 5′ and 3′ are sites of the sugar moiety.

b Each shape or CE transition often mixes MOs. We show the % of the dominant contributor.

c These excitations are taking place from the SOMO to higher UMOs.

d These transitions are taking place from MOs below SOMOs to higher UMOs.

## Results and Discussion

The CAM-B3LYP/6-31G* calculated ground state of ds[5′-G-3′] has 210 doubly occupied MOs. When an excess electron is added vertically to neutral ds[5′-G-3′], it occupies the LUMO (MO number 211) of neutral ds[5′-G-3′] and generates the ground-state TA of ds[5′-G-3′], which has the excess electron on the cytosine base. The excited states of the TA of ds[5′-G-3′] are calculated using the TD-CAM-B3LYP/6-31G* method. As mentioned in the introduction, ds[5′-G-3′] states corresponding to shape resonances are calculated as an electronic transition from the ground state of the TA to higher energy UMOs. Those corresponding to core-excited resonances are the result of an electronic transition of the TA, which places an electron from the core in a UMO; this transition creates a hole in the core of the TA, i.e., two electrons in previously unfilled orbitals of neutral ds[5′-G-3′] in the field of a positive hole.

The TD-CAM-B3LYP/6-31G* calculated transition energies (eV) and oscillator strength (*f*) from the ground-state TA of ds[5′-G-3′] in the gas phase are presented in Table 2. Note that the oscillator strength gives the strength for an optical transition. LEE-induced transitions do not necessarily follow the optical transition moment integrals and therefore transitions with small *f* can occur. From the calculated excited states of the TAs, we identified 4 lowest transitions (D1–D4) forming shape resonances and 12 lowest transitions (CE1–CE12) due to core-excited resonances. We also note that below ca. 4 eV, the lowest 21 transitions are pure shape resonance types, i.e., only the lowest energy SOMO to UMO transitions occurred.^19,39,46,63,64^

### Shape Resonances

The TD-CAM-B3LYP/6-31G* method predicts four lowest excited (D1–D4) states as π(C) → 5′σ*(C), π(C) → 5′σ*(G), π(C) → (π5′σ)*(G), and π(C) → (π3′σ)*(C,G) types having transition energies 0.99, 1.11, 1.50, and 1.56 eV, respectively (Table 2). The σ* states are mostly localized on the PO_4_ group, as shown in SI 1 and 2. D1 and D2 transitions are dominant single transitions, having ca. 95 and 98% contributions, respectively. D3 and D4 are mixed (πσ)* transitions with contributions of 80 and 89%, respectively. These transitions are taking place from the SOMO of type ^2^π and are localized on the cytosine ring in the TA of ds[5′-G-3′] (Figure S1-2 in the SI). The excess electron in the TA localizes over the cytosine ring because the vertical electron affinity of cytosine is higher than that of guanine.^10,65^

The TD-CAM-B3LYP/6-31G* calculated PES of the TA of ds[5′-G-3′] with C5′–O5′ bond elongation is shown in Figure 2; Figure S1-1 of the SI provides an enlarged version. From Figure 2, we see that as the C5′–O5′ bond elongates, the energy of the ground state of the TA (^2^π) surface increases until it crosses the ^2^σ* (D1 surface) with a barrier height of ca. 1.75 eV. The MOs involved in the D1 surface at certain chosen points on the PES are shown in the SI as Figures S1-2 and S2-1–4. The second surface (D2) is also π(C) → 5′σ*(G) type, but it exhibits a bound character. Similarly, the D3 and D4 surfaces of the π(C) → (π5′σ)* type show bound characteristics, as shown in Figure 2. Thus, among D1 to D4 surfaces, only D1 has a dissociative nature. The plots of MOs involved in the transitions and the surface profile of each shape resonance (D1– D4) shown in Figure 1 are presented in Figures S1 and S2 of the SI.

### Core-Excited States (Resonances)

Our TD-CAM-B3LYP/6-31G* calculations predict that the 12 lowest core-excited states (CE1–CE12) of the parent TA of ds[5′-G-3′] lie in the range ca. 4–6 eV, as shown in Table 2. Most of these transitions involve double transitions due to α- and β-electrons from the same MO to higher energy UMOs; more details are shown in Figures S4-1–S7-8 in the SI 4–7. The first core-excited state, CE1, is π(G) → (π5′σ)*(G) in nature (Figure S4-1) and has a transition energy of 3.83 eV and an 82% contribution. CE2 is π(G)→ π*(G) type (Figure S4-2) with a transition energy of 4.03 eV and an 88% contribution. CE3 is a π(G) → (π5′σ)*(G) type transition of 4.88 eV with a 55% contribution. CE4 is a π(C) → 5′σ*(C) transition of 4.95 eV with a 74% contribution. This transition has negligible mixing with shape resonances. CE5 represents π(C) → (π5′σ)*(C,G) with a transition energy of 5.05 eV and a 79% contribution (Figure S4-5). CE6 is of the π(C) → 5′σ*(G) type with a transition energy of 5.06 eV and an 86% contribution. CE7 is a *n*(G) → π*(G) type; the transition energy is 5.13 eV with a 40% contribution (Figure S4-7). CE8 is a π(C) → π*(C) transition; its transition energy is 5.32 eV and it has a low contribution (21%). This transition (CE8) is also mixed with shape resonances with negligible contributions. CE9 is a π(G) → 5′σ*(G) transition of 5.42 eV with an initial 25% contribution at 1.4 Å. However, the contribution of π(G) → 5′σ*(G) increases as the bonds are elongated, reaching 98% at 1.9 Å and maintaining this high fraction until dissociation at 2.3 Å. CE10 belongs to the π(C) → (π5′σ)*(C,G) type with a transition energy and a contribution of 5.47 eV and 62%, respectively. The CE11 π(C) → 3′σ*(G) transition has an energy of 5.51 eV and a contribution of 81%. CE12 represents a transition π(C) → 3′σ*(C) of 5.58 eV having an 84% contribution. We note that among CE1–CE12, only CE4, CE8, CE9, and CE12 have a negligible mixing with shape resonances. This is expected even when using the CASSCF/XMCQDPT2 level of theory; in fact, a strong mixing between shape and core-excited resonances for uracil has been reported by Fennimore and Matsika.^39^

**Figure 4:**
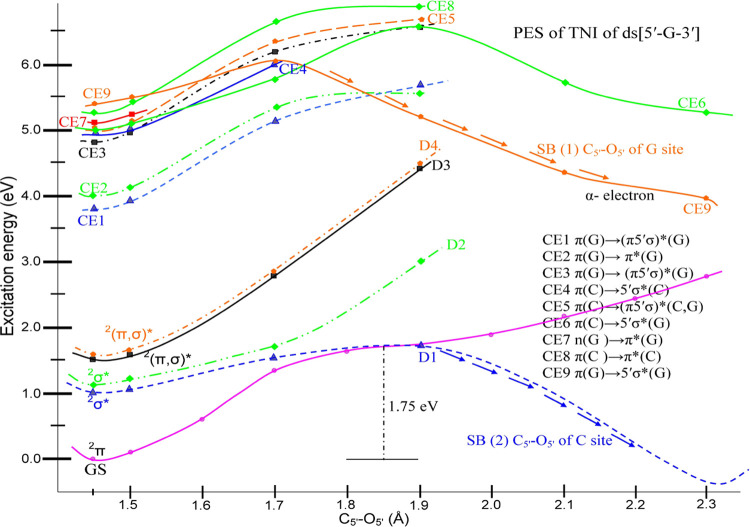
TD-CAM-B3LYP/6-31G* calculated PES of the TA of ds[5′-G-3′] in the neutral optimized geometry of ds[5′-G-3′] with C5′–O5′ bond elongation. Energies and distances are given in eV and Å, respectively, and SB stands for strand break. Lowest curve: ground state (GS) ^2^π-type. Middle curves: D1–D4 are shape resonances. Upper curves: CE1–CE9 are core-excited resonances. CE9 and D1 surfaces lead to strand breaks providing the mechanism for a DSB.

The detailed energy profile of core-excited resonances (CE1–CE9) of the TA of ds[5′-G-3′] with C5′–O5′ bond elongation is shown in Figure 3 (for an enlarged version, see Figure S3-1 in the SI3). From Figure 3, we see that energy profiles of CE1–CE8 are bound in nature, i.e., the energy increases with C5′–O5′ bond elongation. Each surface (CE1–CE9) with MOs and MOs involved in these transitions are calculated at chosen points on the PES, as shown in the SI 3–9. The energy profile of CE9 is of particular interest and shows a very interesting behavior. From Figure 3, we note that CE9 is bound up to 1.7 Å and after surpassing a small barrier of ca. 0.7 eV, it falls very rapidly. Also, below 1.7 Å, the transitions of α- and β-electrons occur from the same (π)MO on guanine and have similar transition energies. From 1.7 Å, the transition energies of α- and β-electrons from the same MO on guanine separate appreciably as the C5′–O5′ bond elongates up to 2.3 Å. The transition energy of the α-electron is significantly lower than the transition energy of the β-electron. The potential energy surface of the TA formed with an extra α-electron shows that this core-excited state could dissociate along the C5′–O5′ bond. For each potential energy surface shown in Figures 2–4, we provide the molecular orbitals (MOs) involved in the transitions as a function of elongation of the C5′–O5′ bond, as shown in the SI 1–9. As shown in Figure S3-10, for the core-excited resonance CE9, the electron wave function also lies in the 5′σ* (PO_4_) orbital of guanine. This localization to phosphate on G extends through the entire CE9 PES from excitation at 1.4 Å to dissociation at 2.3 Å. This is made clear in Figure 3 where the MOs at various points on the PES of CE9 are shown. As expected, at the dissociation limit of the CE9 5′σ* (PO_4_) curve, the α-electron lies only on 5′ PO_4_ of guanine. Thus, from our results summarized in Figure 4, two bond dissociations are predicted: (1) The C5′–O5′ bond dissociation at 5′ PO_4_ of guanine arises from the CE9 core excitation that promotes an inner shell electron to the 5′σ* dissociative path and (2) the C5′–O5′ bond dissociation at 5′ PO_4_ of cytosine induced by the captured electron in the SOMO, which at 1.9 Å, mixes with the 5′σ* (PO_4_) dissociative state and induces dissociation of the C5′–O5′ bond at the cytosine side (see the GS curve in Figure 4). Figure 4 as well as Figure S1-2 show the 5′π*(C) MO to 5′σ* (PO_4_) MO crossover at 1.9 Å, leading to DEA. These results imply that a single LEE of ca. 5 eV on addition to DNA can effectively induce a shape resonance on cytosine and an associated induced core excitation on its base pair guanine, each of which lead to a SSB on the adjacent nucleotides forming an overall DSB.

## Conclusions

The overall potential energy profile of the TAs of ds[5′-G-3′] due to shape and core-excited resonances is shown in Figure 4. The TD-CAM-B3LYP/6-31G* calculation suggests that below 4 eV, only shape resonances occur, whereas above this energy, core-excited resonances are prominent, as generally proposed in the literature.^16,19,39,42,46,63,64^ The calculated 4 lowest shape resonances (D1–D4) occur between ca. 1 and 1.5 eV (Table 2) and only D1 is dissociative in nature. Martin et al.^19^ showed that collision of 0–4 eV electrons with thin DNA films produced single-strand breaks and the corresponding yield function exhibits a sharp peak at 0.8 ± 0.3 eV and a broader feature centered at 2.2 eV. Our calculated lowest shape resonance (D1) around 1 eV is in close agreement with the 0.8 eV maximum reported by Martin et al.^19^ in the SSB yield function of plasmid DNA. Based on electron transmission spectroscopy experiments with the gaseous DNA bases, these authors assigned the 0.8 eV peak to a π* resonance; our calculations categorize D1 as a dissociative σ* configuration. The other 2.2 eV shape resonance observed by Martin et al.^19^ corresponds well to that previously calculated via our TDDFT formalism, which suggested the direct attachment of a 2 eV electron to the PO_4_ group causing a SSB.^34^

The core-excited resonances (CE1–CE9), as shown in Figure 4, lie between 4 and 5.5 eV. Among these, only CE9 has a clearly dissociative nature; it is localized on the PO_4_ group of the 5′-site of the sugar ring attached to the guanine base (Figure 1). The energy profile of the CE9 resonance shows a peculiar behavior. Below 1.7 Å C5′–O5′ bond elongation, the CE9 PES exhibits bound characteristics and has two possible transitions from the same π-MO on guanine to the 5′σ*(G) MO due to the different spins of the α- and β-electrons. However, beyond 1.7 Å of C5′–O5′ bond elongation, the transition energies of α- and β-electrons separate significantly into two different paths (Figure 3). This result clearly indicates that the dissociation process follows the lower energy path of the α-electron. The molecular orbitals of the upper MO for CE9 are shown along the dissociation pathway in Figure 3. Figure 4 shows the pathways to the formation of the DSB in our model of dsDNA. The computed excitations from the TA as a function of both C5′–O5′ bond elongations are shown. CE9 is a clear dissociative pathway for the guanine C5′–O5′ bond, whereas D1 shows a dissociative pathway for the cytosine C5′–O5′ bond. Thus, a double-strand break arises from the combined pathways. As seen in Figure 4, the σ* resonances lead to molecular dissociative pathways such as proton transfers and homo/heterolytic cleavage including DNA strand breaks.^34,49−54,66−68^

Generally, such dissociative states are not accessed directly and decayed by nonradiative mechanism^51^ but they can be populated by vibronic coupling with π → π* transitions, such as the CE8 state (Table 2).^34,49−54^ Thus, while the π → σ* transitions are difficult to observe, the π → π* transitions are bright, longer-lived, and readily populated (Table 2). A π* and σ* coupling^68^ would then lead to a rapid dissociative process resulting in DNA strand cleavage. Finally, we note that the CE9 dissociated state leaves one-electron-oxidized guanine resulting from the electron transfer. This species could also lead to base damage or base release and would then constitute a third type of local damage. Such multiple damages are considered difficult to repair as pointed out previously by Sanche and co-workers.^41,42,69^ Of course, such bond dissociations depend on the sufficient lifetime of the shape or core-excited resonances. Experimental evidence suggests that for energies above the electronic excitation threshold (>4 eV), the lifetime of shape resonances can be too short for DEA to occur,^70^ whereas core-excited TAs, owing to the electron–hole attraction, have sufficient lifetimes to break bonds via DEA, as inferred from the sharp structures they produce in electron transmission spectra.^71^

Luo et al.^41^ and, more recently, Dong et al.^40^ measured LEE yield functions of various DNA lesions. Dong et al.^42^ recorded the yields of base damages, SSBs, cross-links, DSBs, and non-DSB clustered damage induced by 2–20 eV electron impact on plasmid DNA films. Strong maxima were observed at around 5 and 6 eV in all single and cluster damage yield functions, respectively. A unified mechanism causing these damages was proposed, i.e., the capture of a single electron by a base via a core-excited resonance mechanism, followed or not by electron transfer to the phosphate group or another base both in the same or opposite strand. Base damage or removal would occur from DEA on the base, whereas the other lesions implied electron transfer to another base or the phosphate group, followed by DEA at the final electron capture site. Cluster lesions composed of a base damage and a SSB or two base damages on opposite strands could occur if the base was left in a dissociative excited state prior to transfer. The 6 eV maxima in the DSB yield function was proposed to arise from the conversion of a base lesion to a SSB in the initial combination of a base damage with a SSB on the adjacent site. As shown with the present theoretical results, this extra step is not necessary if the incident electron is initially captured by the phosphate group. The present theoretical results clearly indicate that a single 5.4 eV electron can cause a DSB by the incident electron and the associated CE states, each inducing a SSB. Therefore, a more general model of LEE-induced DNA damages should include the possibility of initial electron capture via a core-excited resonance either on a base or on the phosphate group.

As the result of the present work, we now have theoretical and experimental evidence that a single LEE can produce very closely spaced double DNA lesions, which are potentially lethal cluster damages. In fact, such single-electron-hit events within the same DNA molecule have recently been correlated to cell death.^42^ While we considered only such excitations in GC, it is clear that such effects would be expected in AT systems as well. In biological tissues irradiated by high-energy photons or charged particles, cluster damages were previously believed to occur only as the result of multiple independent events, causing more than one lesion within about 7 nm, on the same DNA.^72^ Thus, in future modeling of the sequence of events leading to cellular malfunction or death, single collisions of secondary electrons causing cluster lesions to DNA should be considered. Finally, we note that our results must be considered semiquantitative in nature but they allow us to describe the underlying mechanism of electron-induced double-strand breaks and form a qualitative picture of the likely mechanism of action.

## Supporting Information Available

Supporting Information 1: Potential energy surfaces due to lowest shape resonances (Figures S1-1 to S1-5); Supporting Information 2: Transition energies with molecular orbitals (MOs) due to four lowest shape resonances (D1–D4) (Figures S2-1 to S2-4); Supporting Information 3: Potential energy surfaces due to lowest core-excited shape resonances (Figures S3-1 to S3-10); Supporting Information 4: Transition energies with molecular orbitals (MOs) due to nine lowest core-excited shape resonances (CE1–CE9). Transition energies in eV are calculated at C_5′_–O_5′_ distance 1.44 Å (Figures S4-1 to S4-9); Supporting Information 5: Transition energies with molecular orbitals (MOs) due to nine lowest core-excited shape resonances (CE1–CE9). Transition energies in eV are calculated at C_5′_–O_5′_ distance 1.50 Å (Figures S5-1 to S5-9); Supporting Information 6: Transition energies with molecular orbitals (MOs) due to nine lowest core-excited shape resonances (CE1–CE9). Transition energies in eV are calculated at C_5′_–O_5′_ distance 1.70 Å (Figures S6-1 to S6-8); Supporting Information 7: Transition energies with molecular orbitals (MOs) due to eight lowest core-excited shape resonances (CE1–CE8). Transition energies in eV are calculated at C_5′_–O_5′_ distance 1.90 Å (Figures S7-1 to S7-8); Supporting Information 8: Transition energies with molecular orbitals (MOs) due to three lowest core-excited shape resonances (CE1–CE9). Transition energies in eV are calculated at C_5′_–O_5′_ distance 2.10 Å (Figures S8-1 to S8-3) (PDF)
