# Supplementary material for: How a Single 5 eV Electron Can Induce Double-Strand Breaks in DNA: A Time-Dependent Density Functional Theory Study
Source: J Phys Chem B. 2024 Apr 23;128(17):4053–62. doi: 10.1021/acs.jpcb.3c08367 (PMC11075081; doi:10.1021/acs.jpcb.3c08367)
Supplement: Supplementary file 1 — jp3c08367_si_001.pdf [file jp3c08367_si_001.pdf]

Supporting Information for publication

How a single 5 eV Electron can induce a Double Strand Breaks in DNA: A Time-Dependent Density Functional Theory Study

Anil Kumar,[a] Michael Sevilla[a] and Leon Sanche[b]

[a]Department of Chemistry, Oakland University, Rochester, MI 48309

[b]Department of Nuclear Medicine and Radiobiology and Clinical Research Center, Faculty of Medicine and Health Sciences, Université de Sherbrooke, Sherbrooke, QC Canada J1H 5N4

Sections:

Supporting Information 1: Figures S1-1 to S1-5. Potential energy surfaces due to lowest shape resonances.

Supporting Information 2: Figures S2-1 to S2-4. Transition energies with molecular orbitals (MOs) due to four lowest shape resonances (D1 – D4).

Supporting Information 3: Figures S3-1 to S3-10. Potential energy surfaces due to lowest core excited shape resonances.

Supporting Information 4: Figures S4-1 to S4-9. Transition energies with molecular orbitals (MOs) due to nine lowest core excited shape resonances (CE1 – CE9). Transition energies in eV are calculated at $C_{5'}$-$O_{5'}$ distance 1.44 Å.

Supporting Information 5: Figures S5-1 to S5-9. Transition energies with molecular orbitals (MOs) due to nine lowest core excited shape resonances (CE1 – CE9). Transition energies in eV are calculated at $C_{5'}$-$O_{5'}$ distance 1.50 Å.

Supporting Information 6: Figures S6-1 to S6-8. Transition energies with molecular orbitals (MOs) due to nine lowest core excited shape resonances (CE1 – CE9). Transition energies in eV are calculated at $C_{5'}$-$O_{5'}$ distance 1.70 Å.

Supporting Information 7: Figures S7-1 to S7-8. Transition energies with molecular orbitals (MOs) due to eight lowest core excited shape resonances (CE1 – CE8). Transition energies in eV are calculated at $C_{5'}$-$O_{5'}$ distance 1.90 Å.

Supporting Information 8: Figures S8-1 to S8-3. Transition energies with molecular orbitals (MOs) due to three lowest core excited shape resonances (CE1 – CE9). Transition energies in eV are calculated at $C_{5'}$-$O_{5'}$ distance 2.10 Å

Supporting Information 1

Potential energy surfaces due to lowest shape resonances.

Contents

Figure S1-1- Cam-b3lyp/6-31G* calculated potential energy surface (PES) of ds[5′-G-3′] transient anion (TA) due to 4 lowest shape resonances (D1 – D4) calculated in the neutral optimized geometry of ds[5′-G-3′] with C5′-O5′ bond elongation. Energies and distances are given in eV and Å, respectively. π, σ designate the nature of each surface on the PES while GS and asterisk (*) designate the ground and excited state surfaces.

Figure S1-2- Cam-b3lyp/6-31G* calculated potential energy surface (PES) of ds[5′-G-3′] transient anion (TA) due to 1st shape resonance; calculated in the neutral optimized geometry of ds[5′-G-3′] with C5′-O5′ bond elongation. Energies and distances are given in eV and Å, respectively. Molecular orbitals (MO) involved in transition are shown. π, σ designate the nature of MOs, GS and asterisk (*) designate the ground and excited state. Singly occupied molecular orbital (SOMO) is shown below GS curve.

Figure S1-3- Cam-b3lyp/6-31G* calculated potential energy surface (PES) of ds[5′-G-3′] transient anion (TA) due to 2nd shape resonance; calculated in the neutral optimized geometry of ds[5′-G-3′] with C5′-O5′ bond elongation. Energies and distances are given in eV and Å, respectively. Molecular orbitals (MO) involved in transition are shown. π, σ designate the nature of MOs, GS and asterisk (*) designate the ground and excited state. Singly occupied molecular orbital (SOMO) is shown below GS curve.

Figure S1-4- Cam-b3lyp/6-31G* calculated potential energy surface (PES) of ds[5′-G-3′] transient anion (TA) due to 3rd shape resonance; calculated in the neutral optimized geometry of ds[5′-G-3′] with C5′-O5′ bond elongation. Energies and distances are given in eV and Å, respectively. Molecular orbitals (MO) involved in transition are shown. π, σ designate the nature of MOs, GS and asterisk (*) designate the ground and excited state. Singly occupied molecular orbital (SOMO) is shown below GS curve.

Figure S1-5- Cam-b3lyp/6-31G* calculated potential energy surface (PES) of ds[5′-G-3′] transient anion (TA) due to 4th shape resonance; calculated in the neutral optimized geometry of ds[5′-G-3′] with C5′-O5′ bond elongation. Energies and distances are given in eV and Å, respectively. Molecular orbitals (MO) involved in transition are shown. π, σ designate the nature of MOs, GS and asterisk (*) designate the ground and excited state. Singly occupied molecular orbital (SOMO) is shown below GS curve.

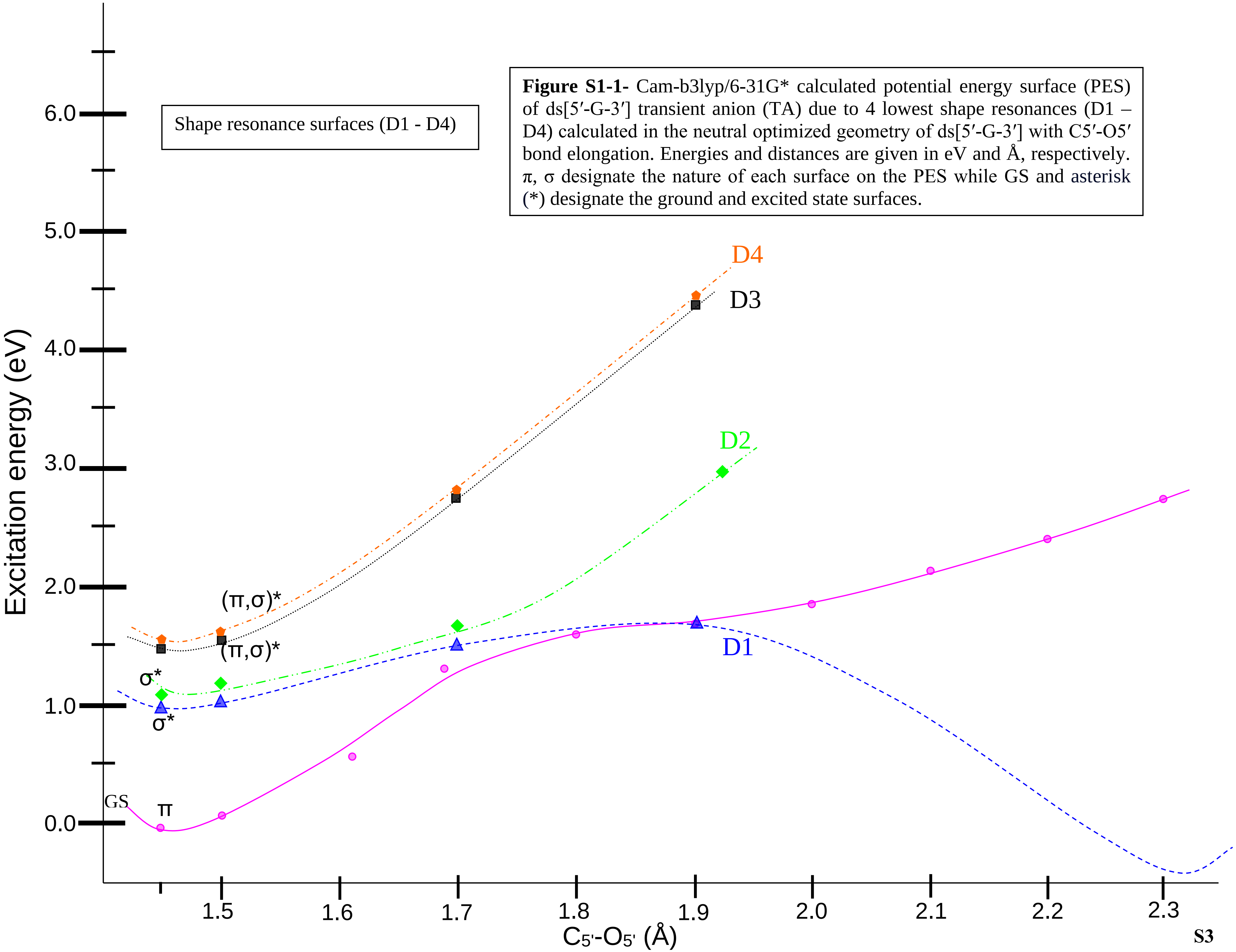

Figure S1-1- Cam-b3lyp/6-31G* calculated potential energy surface (PES) of ds[5′-G-3′] transient anion (TA) due to 4 lowest shape resonances (D1 – D4) calculated in the neutral optimized geometry of ds[5′-G-3′] with C5′-O5′ bond elongation. Energies and distances are given in eV and Å, respectively. π, σ designate the nature of each surface on the PES while GS and asterisk (*) designate the ground and excited state surfaces.

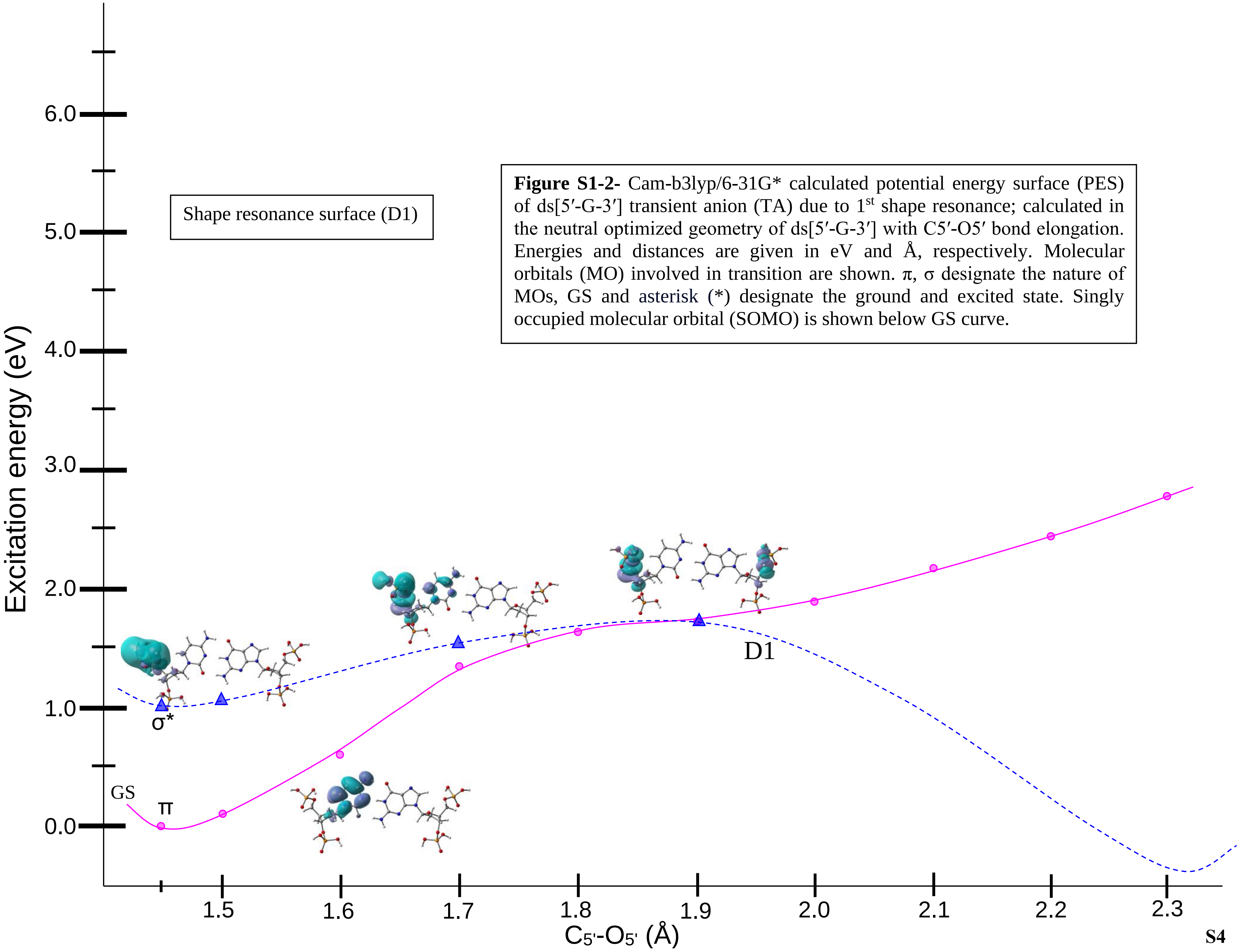

Figure S1-2- Cam-b3lyp/6-31G* calculated potential energy surface (PES) of ds[5′-G-3′] transient anion (TA) due to 1st shape resonance; calculated in the neutral optimized geometry of ds[5′-G-3′] with C5′-O5′ bond elongation. Energies and distances are given in eV and Å, respectively. Molecular orbitals (MO) involved in transition are shown. π, σ designate the nature of MOs, GS and asterisk (*) designate the ground and excited state. Singly occupied molecular orbital (SOMO) is shown below GS curve.

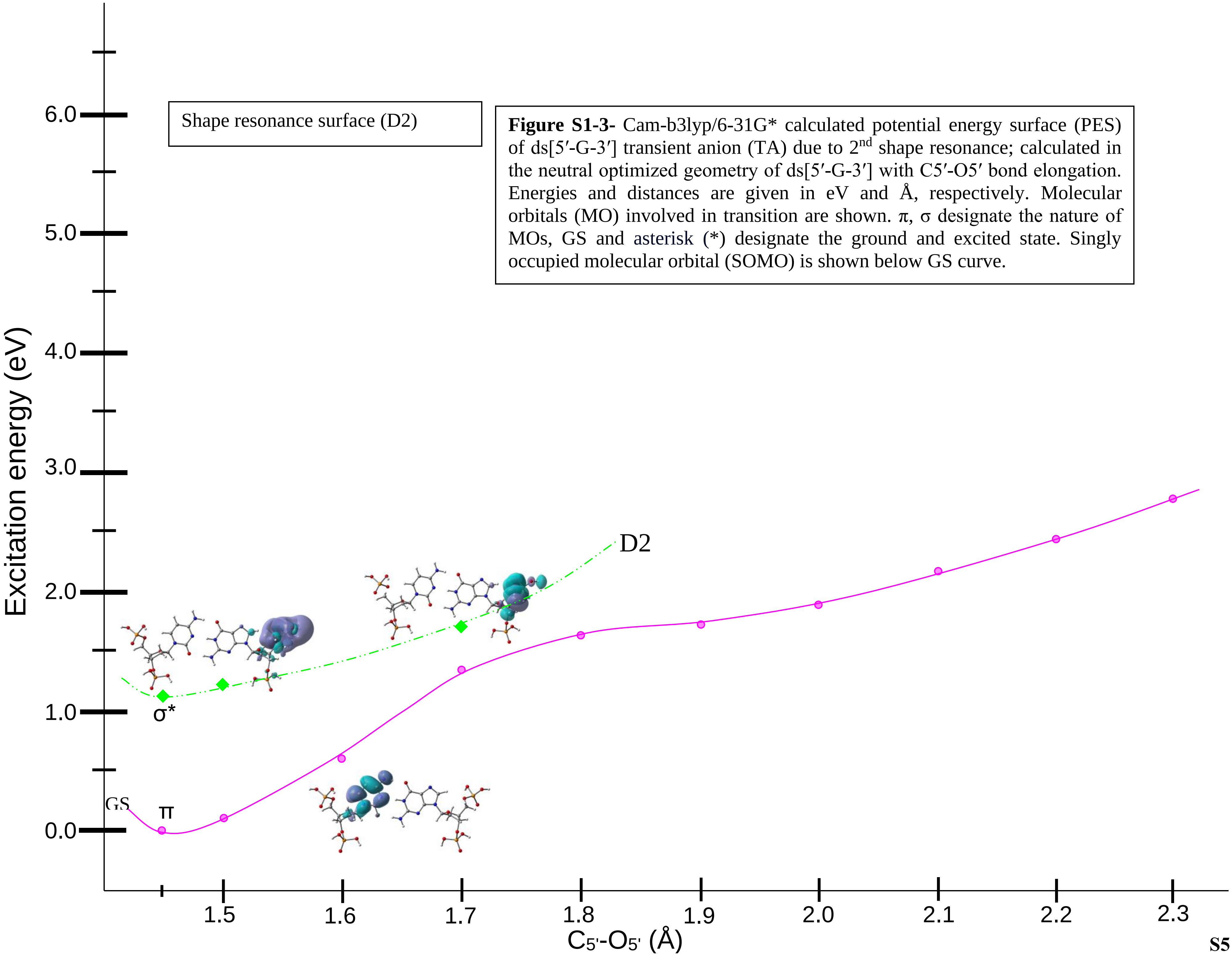

Figure S1-3- Cam-b3lyp/6-31G* calculated potential energy surface (PES) of ds[5′-G-3′] transient anion (TA) due to 2nd shape resonance; calculated in the neutral optimized geometry of ds[5′-G-3′] with C5′-O5′ bond elongation. Energies and distances are given in eV and Å, respectively. Molecular orbitals (MO) involved in transition are shown. π, σ designate the nature of MOs, GS and asterisk (*) designate the ground and excited state. Singly occupied molecular orbital (SOMO) is shown below GS curve.

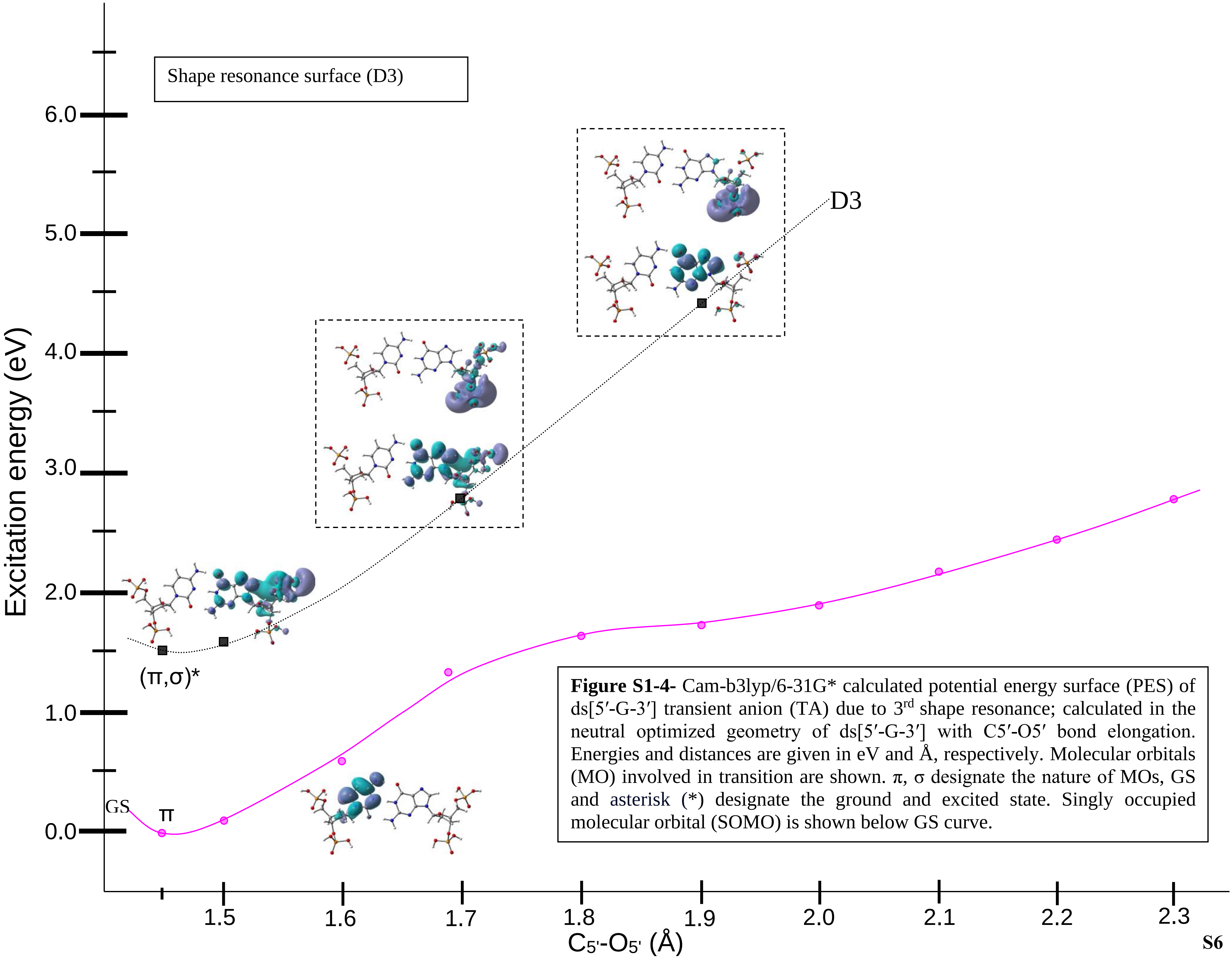

Figure S1-4- Cam-b3lyp/6-31G* calculated potential energy surface (PES) of ds[5′-G-3′] transient anion (TA) due to 3rd shape resonance; calculated in the neutral optimized geometry of ds[5′-G-3′] with C5′-O5′ bond elongation. Energies and distances are given in eV and Å, respectively. Molecular orbitals (MO) involved in transition are shown. π, σ designate the nature of MOs, GS and asterisk (*) designate the ground and excited state. Singly occupied molecular orbital (SOMO) is shown below GS curve.

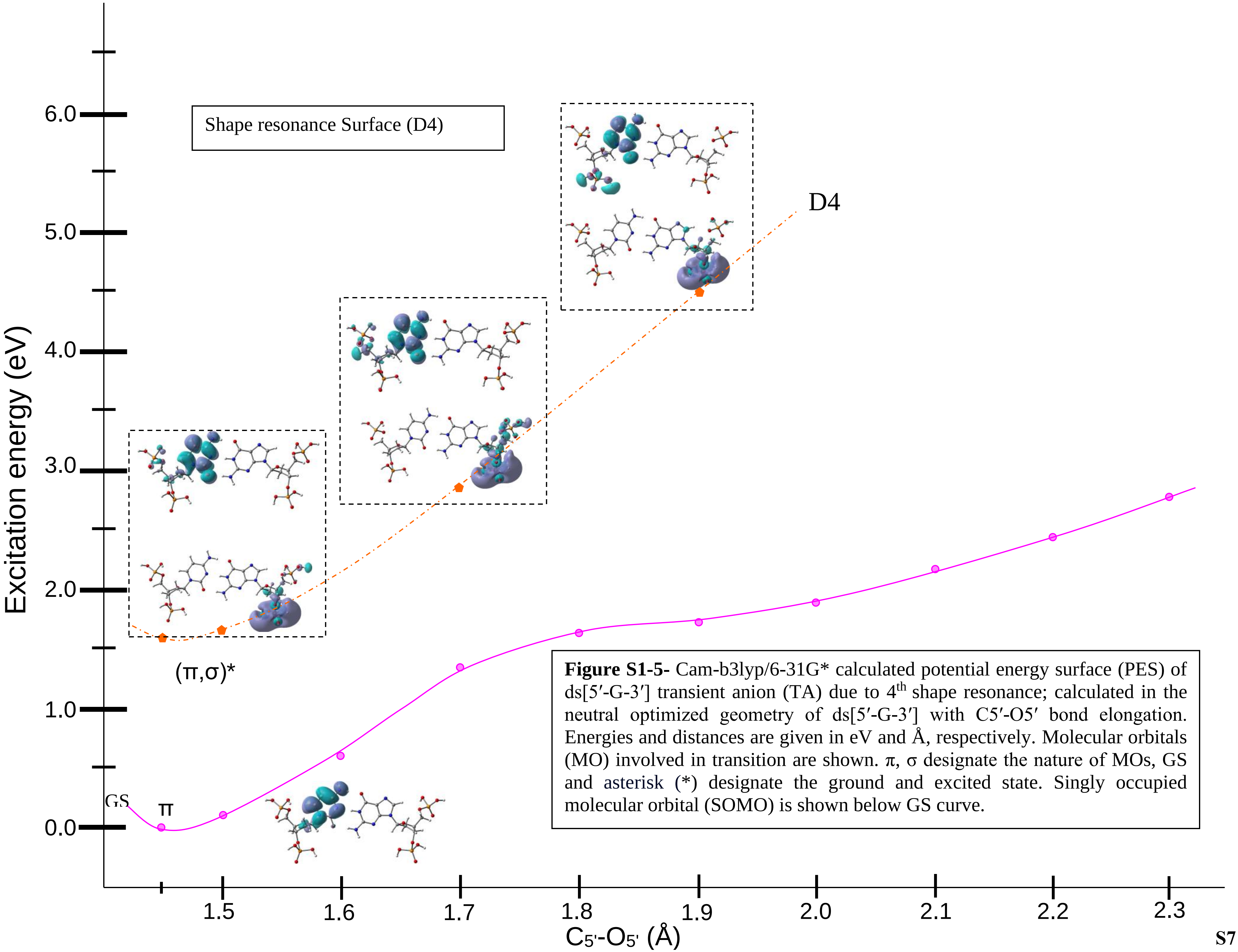

Figure S1-5- Cam-b3lyp/6-31G* calculated potential energy surface (PES) of ds[5′-G-3′] transient anion (TA) due to 4th shape resonance; calculated in the neutral optimized geometry of ds[5′-G-3′] with C5′-O5′ bond elongation. Energies and distances are given in eV and Å, respectively. Molecular orbitals (MO) involved in transition are shown. π, σ designate the nature of MOs, GS and asterisk (*) designate the ground and excited state. Singly occupied molecular orbital (SOMO) is shown below GS curve.

Supporting Information 2

Transition energies with molecular orbitals (MOs) due to four lowest shape resonances (D1 – D4)

Figure S2-1- Cam-b3lyp/6-31G* calculated transition energies of ds[5′-G-3′] transient anion (TA); calculated in the neutral optimized geometry of ds[5′-G-3′]. Energies are given in eV, respectively. Transitions (D1 – D4) are taking place from the filled LUMO (previously the lowest unoccupied molecular orbital now the SOMO) (D_0 (MO no. 211)) to higher UMOs (unoccupied molecular orbital) (. Arrow shows the filled α-electron.

Figure S2-2- Cam-b3lyp/6-31G* calculated transition energies of ds[5′-G-3′] transient anion (TA); calculated in the neutral optimized geometry of ds[5′-G-3′] by vertically stretching the $C_{5'}$-$O_{5'}$ bond at 1.50 (Å). Energies are given in eV, respectively. Transitions (D1 – D4) are taking place from the filled LUMO (previously the lowest unoccupied molecular orbital now the SOMO) (D_0 (MO no. 211)) to higher UMOs. Arrow shows the filled α-electron.

.

Figure S2-3- Cam-b3lyp/6-31G* calculated transition energies of ds[5′-G-3′] transient anion (TA); calculated in the neutral optimized geometry of ds[5′-G-3′] by vertically stretching the $C_{5'}$-$O_{5'}$ bond at 1.70 (Å). Energies are given in eV, respectively. Transitions (D1 – D4) are taking place from the filled LUMO (previously the lowest unoccupied molecular orbital now the SOMO) (D_0 (MO no. 211)) to higher UMOs. Arrow shows the filled α-electron.

Figure S2-4- Cam-b3lyp/6-31G* calculated transition energies of ds[5′-G-3′] transient anion (TA); calculated in the neutral optimized geometry of ds[5′-G-3′] by vertically stretching the $C_{5'}$-$O_{5'}$ bond at 1.90 (Å). Energies are given in eV, respectively. Transitions (D1 – D4) are taking place from the filled LUMO (previously the lowest unoccupied molecular orbital now the SOMO) (D_0 (MO no. 211)) to higher UMOs. Arrow shows the filled α-electron.

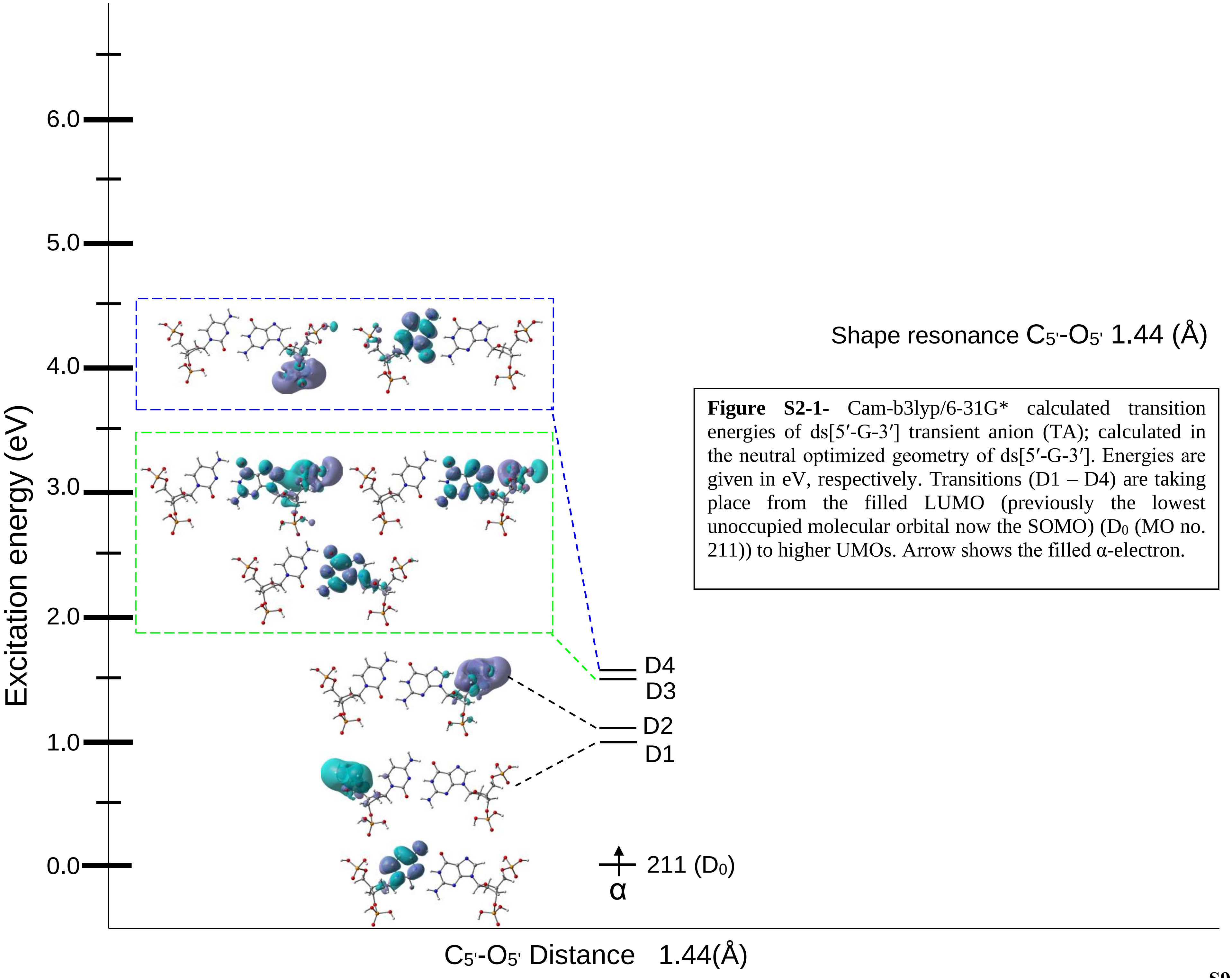

Figure S2-1- Cam-b3lyp/6-31G* calculated transition energies of ds[5′-G-3′] transient anion (TA); calculated in the neutral optimized geometry of ds[5′-G-3′]. Energies are given in eV, respectively. Transitions (D1 – D4) are taking place from the filled LUMO (previously the lowest unoccupied molecular orbital now the SOMO) (D_0 (MO no. 211)) to higher UMOs. Arrow shows the filled α-electron.

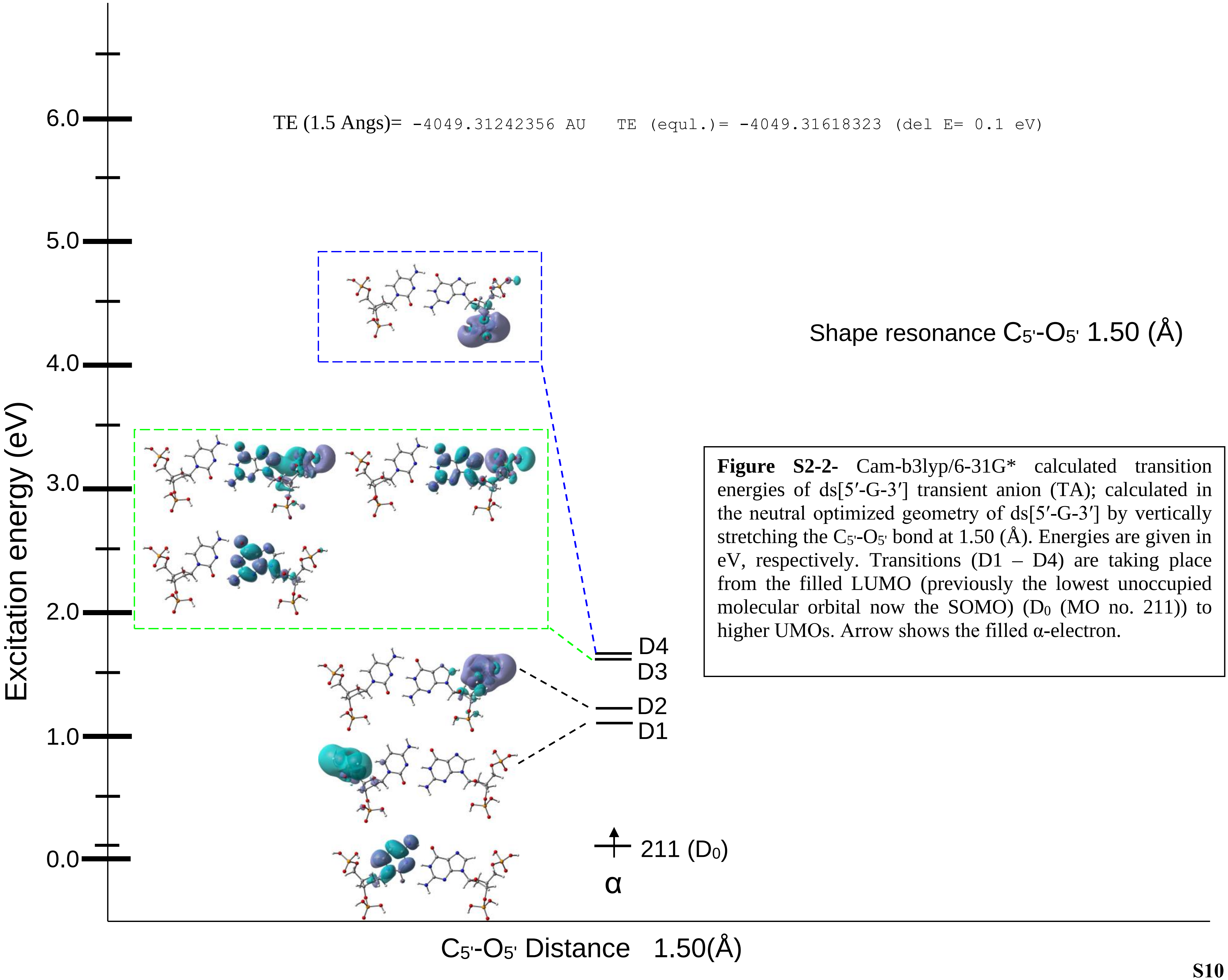

Figure S2-2- Cam-b3lyp/6-31G* calculated transition energies of ds[5′-G-3′] transient anion (TA); calculated in the neutral optimized geometry of ds[5′-G-3′] by vertically stretching the $C_{5'}$-$O_{5'}$ bond at 1.50 (Å). Energies are given in eV, respectively. Transitions (D1 – D4) are taking place from the filled LUMO (previously the lowest unoccupied molecular orbital now the SOMO) (D_0 (MO no. 211)) to higher UMOs. Arrow shows the filled α-electron.

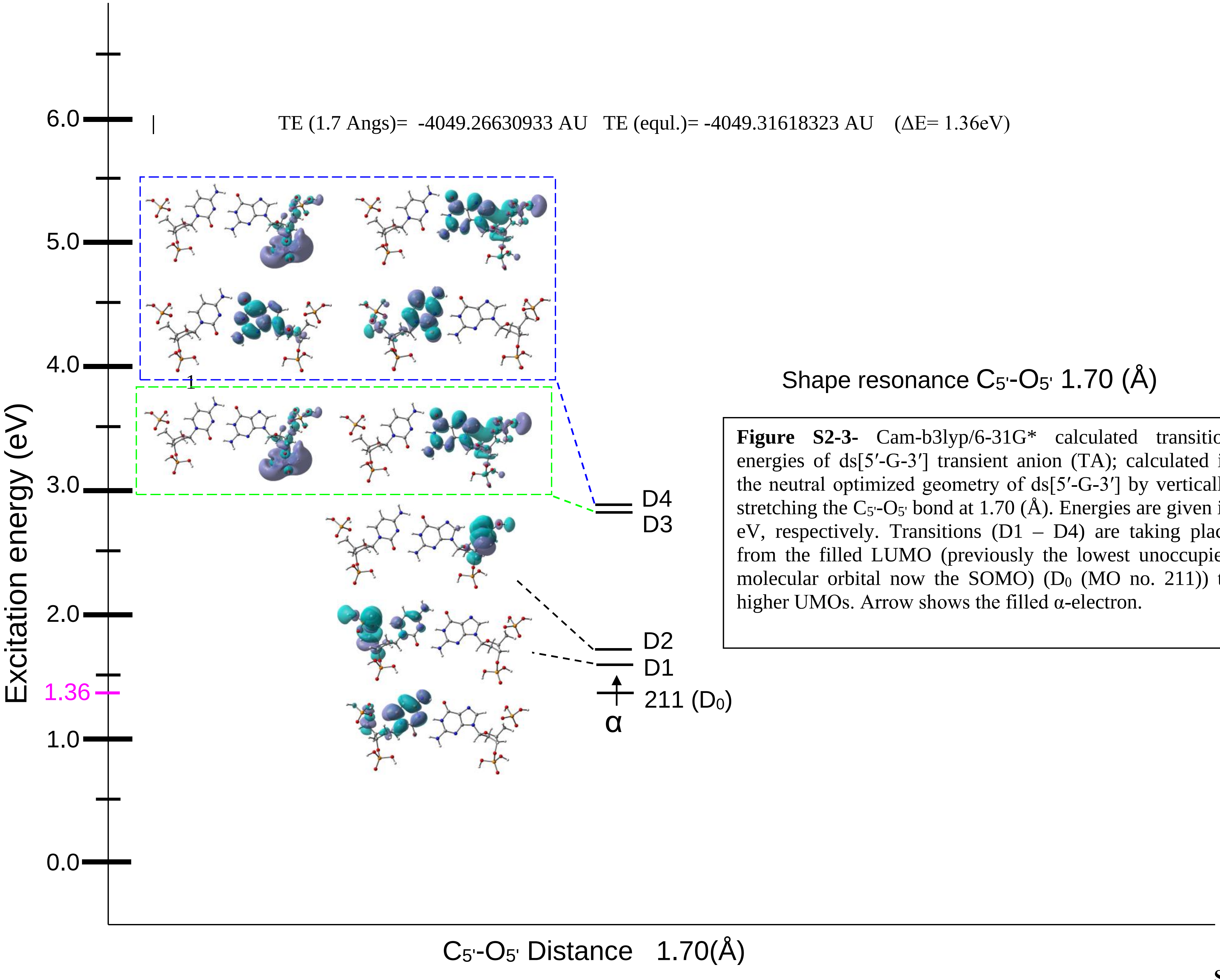

Figure S2-3- Cam-b3lyp/6-31G* calculated transition energies of ds[5′-G-3′] transient anion (TA); calculated in the neutral optimized geometry of ds[5′-G-3′] by vertically stretching the $C_{5'}$-$O_{5'}$ bond at 1.70 (Å). Energies are given in eV, respectively. Transitions (D1 – D4) are taking place from the filled LUMO (previously the lowest unoccupied molecular orbital now the SOMO) (D_0 (MO no. 211)) to higher UMOs. Arrow shows the filled α-electron.

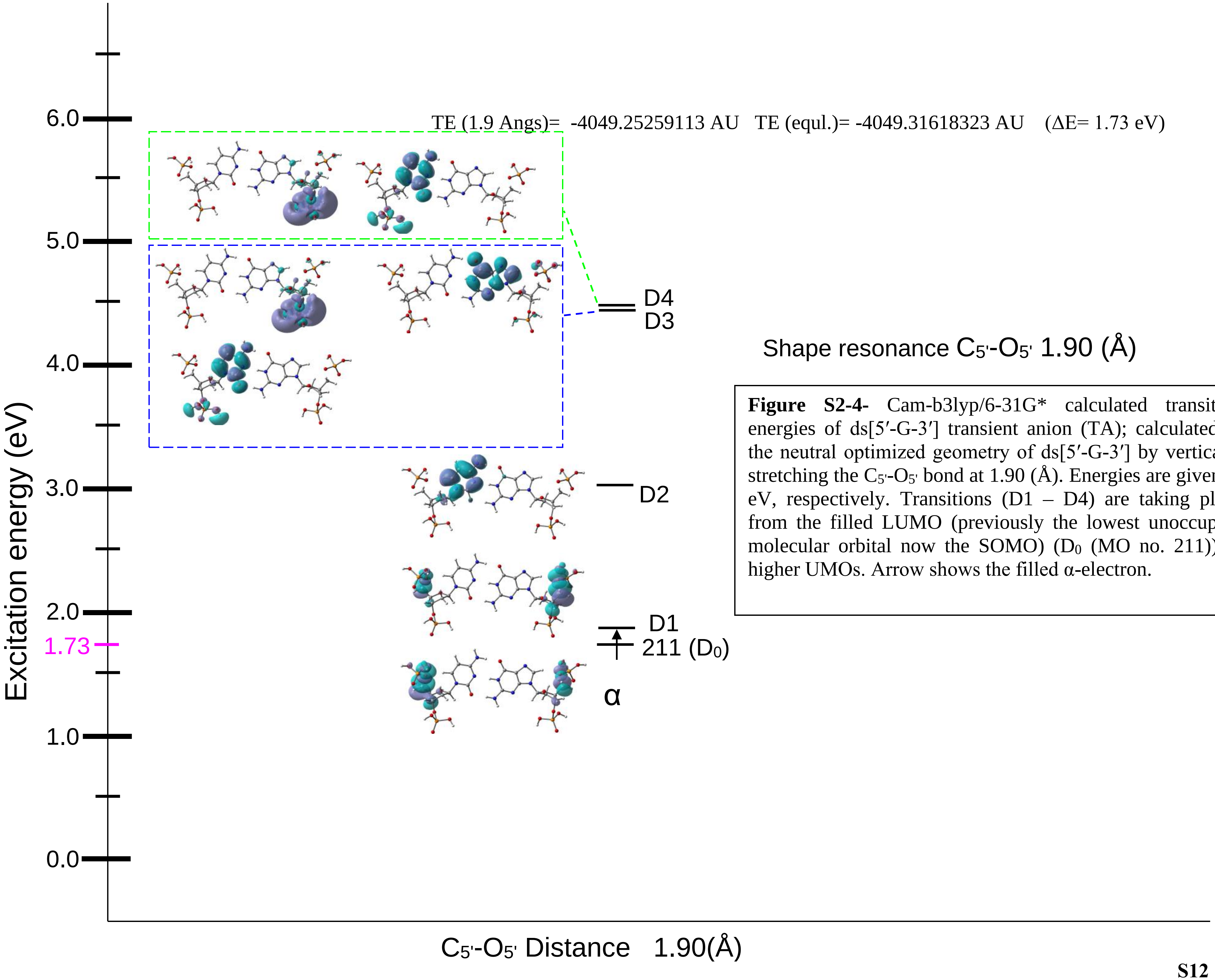

Figure S2-4- Cam-b3lyp/6-31G* calculated transition energies of ds[5′-G-3′] transient anion (TA); calculated in the neutral optimized geometry of ds[5′-G-3′] by vertically stretching the $C_{5'}$-$O_{5'}$ bond at 1.90 (Å). Energies are given in eV, respectively. Transitions (D1 – D4) are taking place from the filled LUMO (previously the lowest unoccupied molecular orbital now the SOMO) (D_0 (MO no. 211)) to higher UMOs. Arrow shows the filled α-electron.

Supporting Information 3

Potential energy surfaces due to lowest core excited shape resonances

Contents

Figure S3-1- Cam-b3lyp/6-31G* calculated lowest nine core excited resonance potential energy surface (PES) of ds[5′-G-3′] transient anion (TA); calculated in the neutral optimized geometry of ds[5′-G-3′] with C5′-O5′ bond elongation. Energies and distances are given in eV and Å, respectively. Lowest curve: Ground state (GS) π-type. Upper curves: CE1 – CE9 are core excited resonances. For nature of each core excited surface, see Figures **S3-2** to **S3-10**.

Figure S3-2- Cam-b3lyp/6-31G* calculated core excited resonance (CE1) potential energy surface (PES) of ds[5′-G-3′] transient anion (TA); calculated in the neutral optimized geometry of ds[5′-G-3′] with C5′-O5′ bond elongation. Energies and distances are given in eV and Å, respectively. Lowest curve: Ground state (GS) π-type. Upper curve: (πσ)*-type.

Figure S3-3- Cam-b3lyp/6-31G* calculated core excited resonance (CE2) potential energy surface (PES) of ds[5′-G-3′] transient anion (TA); calculated in the neutral optimized geometry of ds[5′-G-3′] with C5′-O5′ bond elongation. Energies and distances are given in eV and Å, respectively. Lowest curve: Ground state (GS) π-type. Upper curve: π*-type.

Figure S3-4- Cam-b3lyp/6-31G* calculated core excited resonance (CE3) potential energy surface (PES) of ds[5′-G-3′] transient anion (TA); calculated in the neutral optimized geometry of ds[5′-G-3′] with C5′-O5′ bond elongation. Energies and distances are given in eV and Å, respectively. Lowest curve: Ground state (GS) π-type. Upper curve: π*-type.

Figure S3-5- Cam-b3lyp/6-31G* calculated core excited resonance (CE4) potential energy surface (PES) of ds[5′-G-3′] transient anion (TA); calculated in the neutral optimized geometry of ds[5′-G-3′] with C5′-O5′ bond elongation. Energies and distances are given in eV and Å, respectively. Lowest curve: Ground state (GS) π-type. Upper curve: σ*-type.

Figure S3-6- Cam-b3lyp/6-31G* calculated core excited resonance (CE5) potential energy surface (PES) of ds[5′-G-3′] transient anion (TA); calculated in the neutral optimized geometry of ds[5′-G-3′] with C5′-O5′ bond elongation. Energies and distances are given in eV and Å, respectively. Lowest curve: Ground state (GS) π-type. Upper curve: π*-type.

Figure S3-7- Cam-b3lyp/6-31G* calculated core excited resonance (CE6) potential energy surface (PES) of ds[5′-G-3′] transient anion (TA); calculated in the neutral optimized geometry of ds[5′-G-3′] with C5′-O5′ bond elongation. Energies and distances are given in eV and Å, respectively. Lowest curve: Ground state (GS) π-type. Upper curve: σ*-type.

Figure S3-8- Cam-b3lyp/6-31G* calculated core excited resonance (CE7) potential energy surface (PES) of ds[5′-G-3′] transient anion (TA); calculated in the neutral optimized geometry of ds[5′-G-3′] with C5′-O5′ bond elongation. Energies and distances are given in eV and Å, respectively. Lowest curve: Ground state (GS) n-type. Upper curve: π*-type.

Figure S3-9- Cam-b3lyp/6-31G* calculated core excited resonance (CE8) potential energy surface (PES) of ds[5′-G-3′] transient anion (TA); calculated in the neutral optimized geometry of ds[5′-G-3′] with C5′-O5′ bond elongation. Energies and distances are given in eV and Å, respectively. Lowest curve: Ground state (GS) π-type. Upper curve: π*-type.

Figure S3-10- Cam-b3lyp/6-31G* calculated core excited resonance (CE9) potential energy surface (PES) of ds[5′-G-3′] transient anion (TA); calculated in the neutral optimized geometry of ds[5′-G-3′] with C5′-O5′ bond elongation. Energies and distances are given in eV and Å, respectively. Lowest curve: Ground state (GS) π-type. Upper curve: σ*-type.

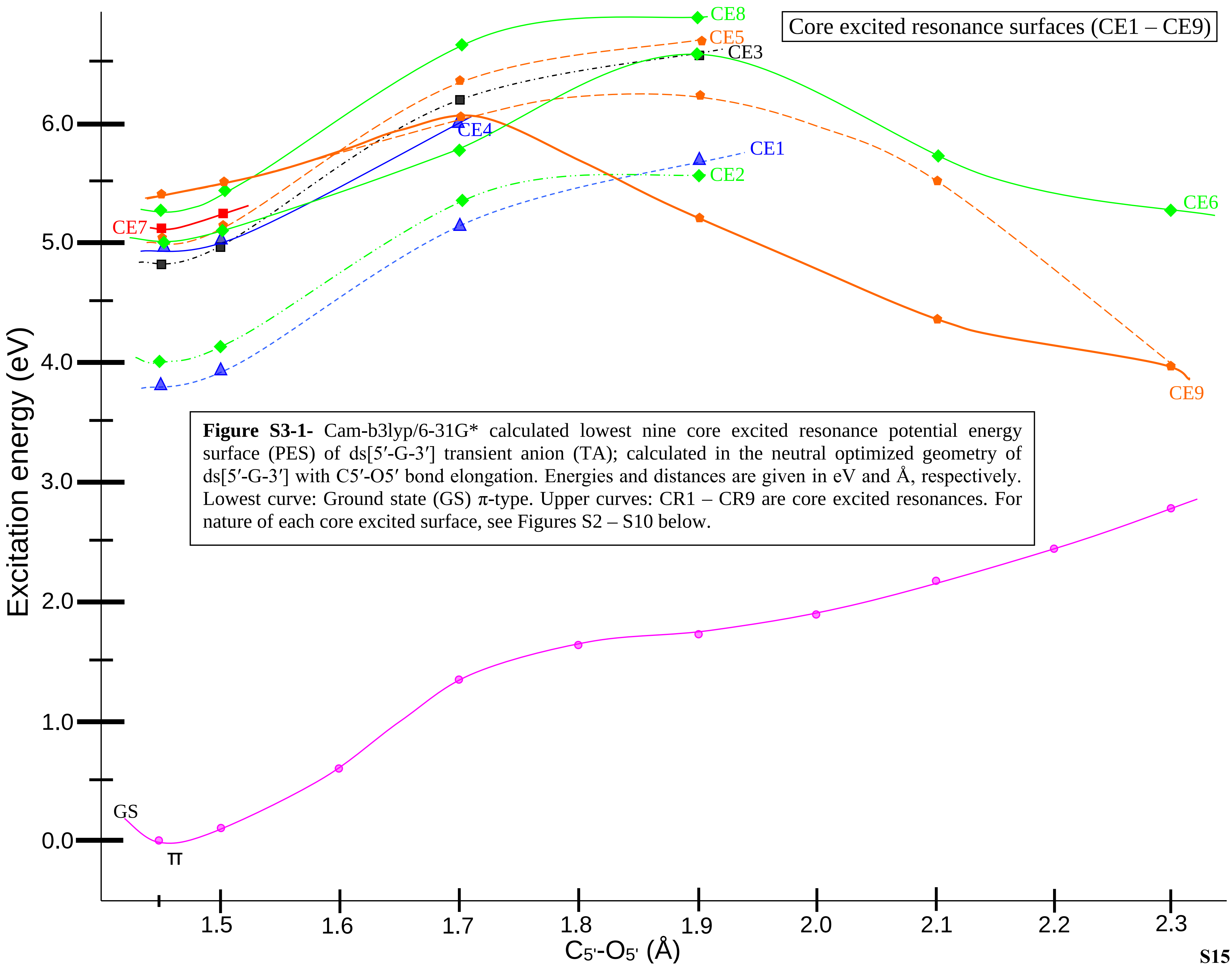

Figure S3-1- Cam-b3lyp/6-31G* calculated lowest nine core excited resonance potential energy surface (PES) of ds[5′-G-3′] transient anion (TA); calculated in the neutral optimized geometry of ds[5′-G-3′] with C5′-O5′ bond elongation. Energies and distances are given in eV and Å, respectively. Lowest curve: Ground state (GS) π-type. Upper curves: CR1 – CR9 are core excited resonances. For nature of each core excited surface, see Figures S2 – S10 below.

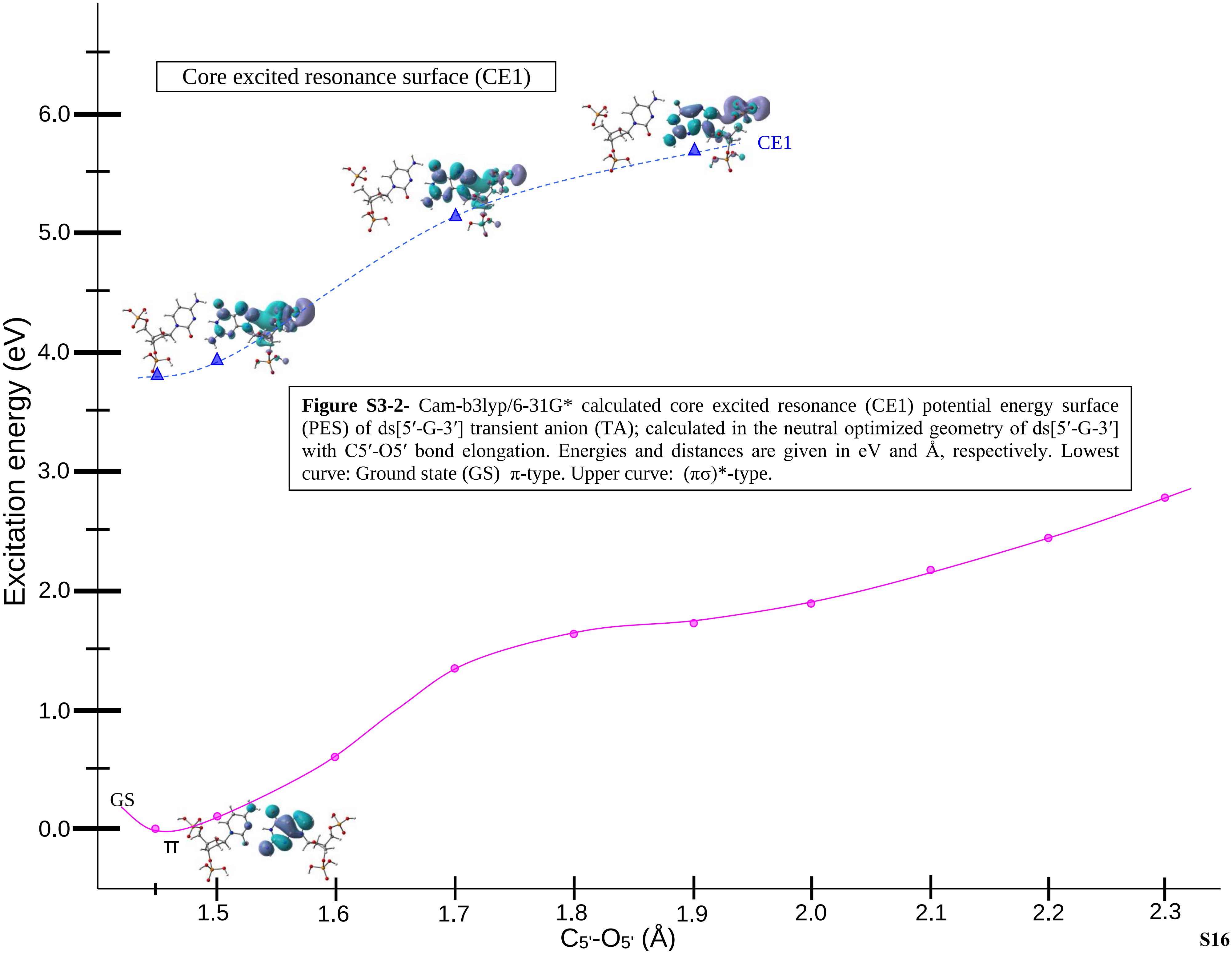

Figure S3-2- Cam-b3lyp/6-31G* calculated core excited resonance (CE1) potential energy surface (PES) of ds[5′-G-3′] transient anion (TA); calculated in the neutral optimized geometry of ds[5′-G-3′] with C5′-O5′ bond elongation. Energies and distances are given in eV and Å, respectively. Lowest curve: Ground state (GS) π-type. Upper curve: (πσ)*-type.

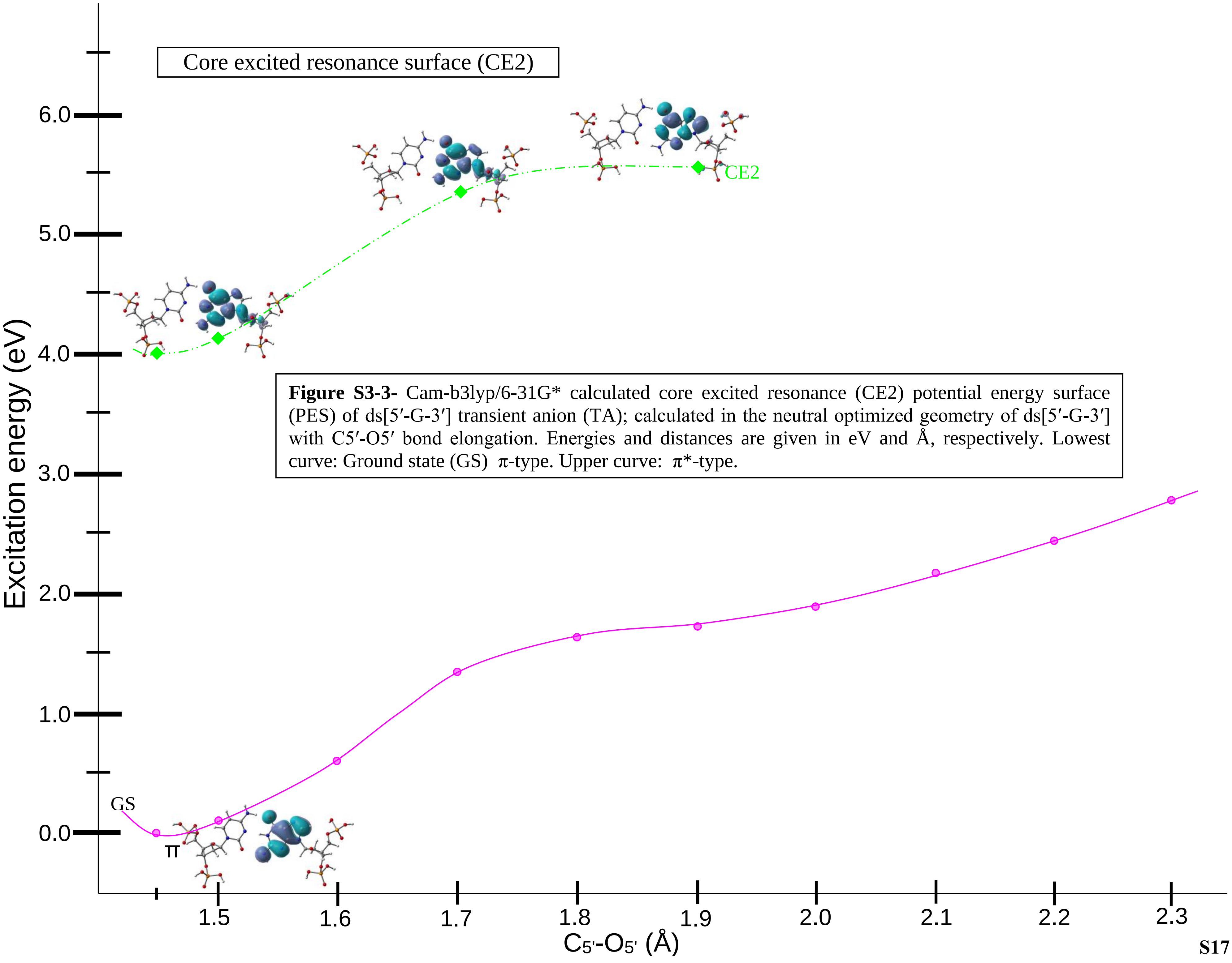

Figure S3-3- Cam-b3lyp/6-31G* calculated core excited resonance (CE2) potential energy surface (PES) of ds[5′-G-3′] transient anion (TA); calculated in the neutral optimized geometry of ds[5′-G-3′] with C5′-O5′ bond elongation. Energies and distances are given in eV and Å, respectively. Lowest curve: Ground state (GS) π-type. Upper curve: π*-type.

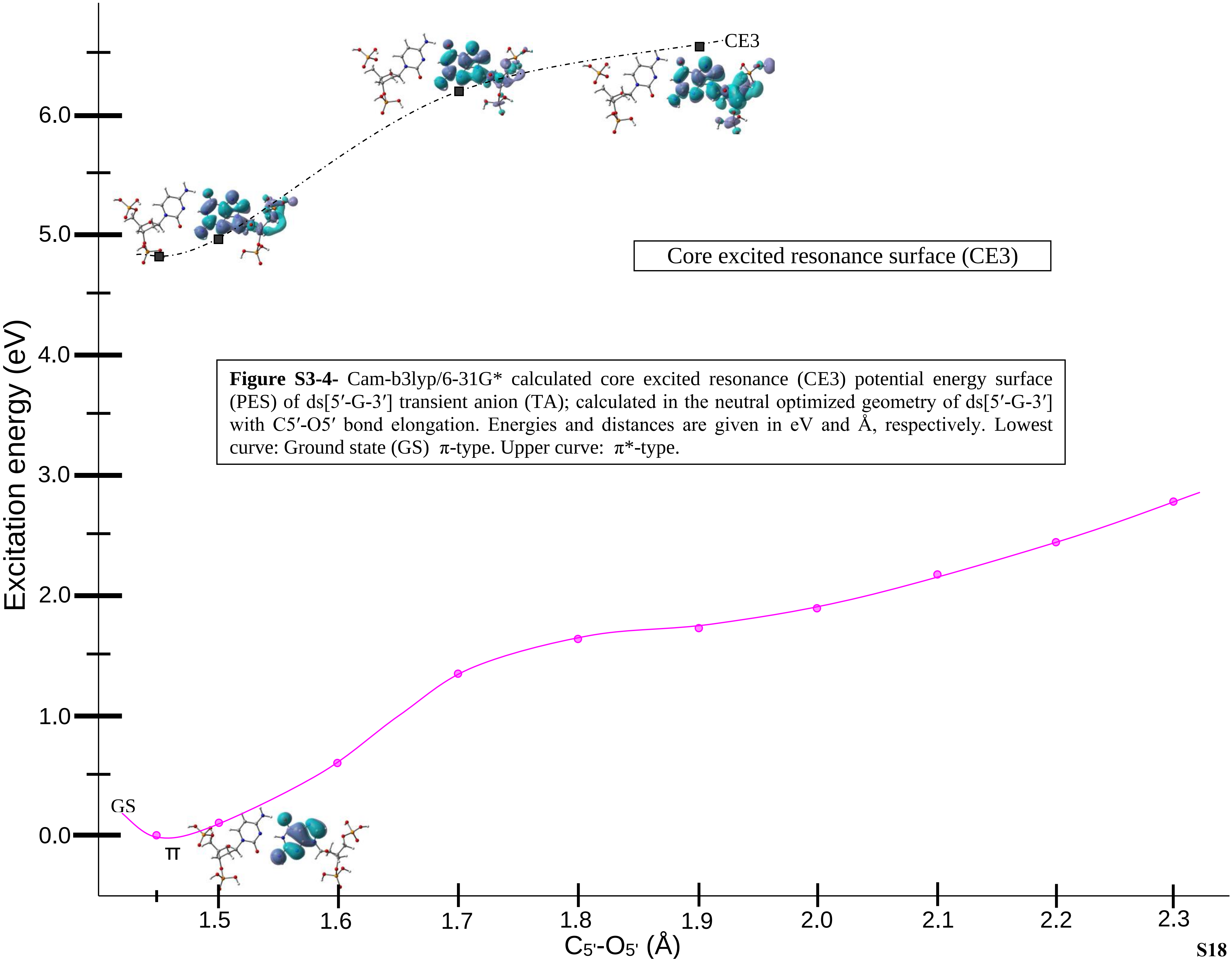

Figure S3-4- Cam-b3lyp/6-31G* calculated core excited resonance (CE3) potential energy surface (PES) of ds[5′-G-3′] transient anion (TA); calculated in the neutral optimized geometry of ds[5′-G-3′] with C5′-O5′ bond elongation. Energies and distances are given in eV and Å, respectively. Lowest curve: Ground state (GS) π-type. Upper curve: π*-type.

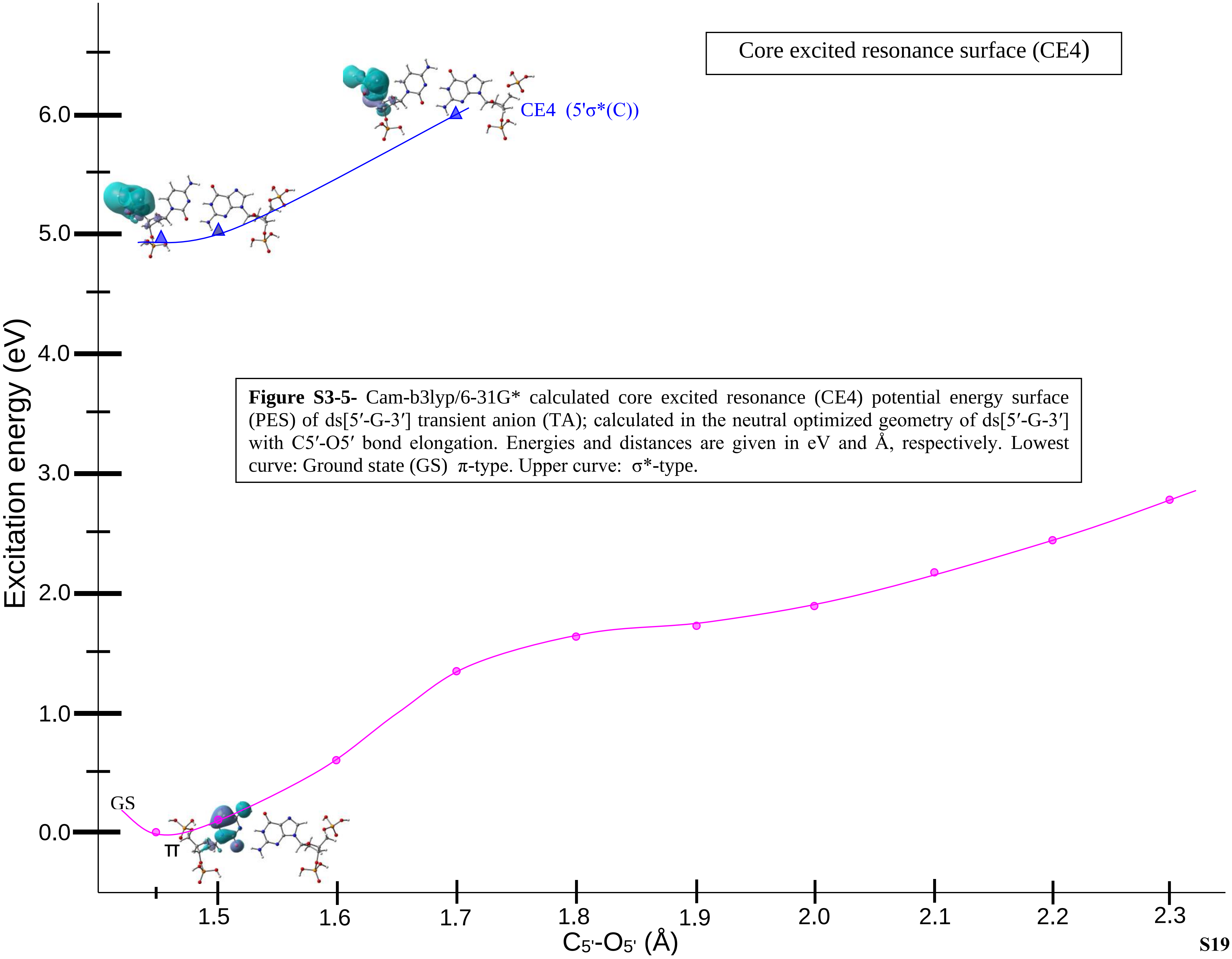

Figure S3-5- Cam-b3lyp/6-31G* calculated core excited resonance (CE4) potential energy surface (PES) of ds[5′-G-3′] transient anion (TA); calculated in the neutral optimized geometry of ds[5′-G-3′] with C5′-O5′ bond elongation. Energies and distances are given in eV and Å, respectively. Lowest curve: Ground state (GS) π-type. Upper curve: σ*-type.

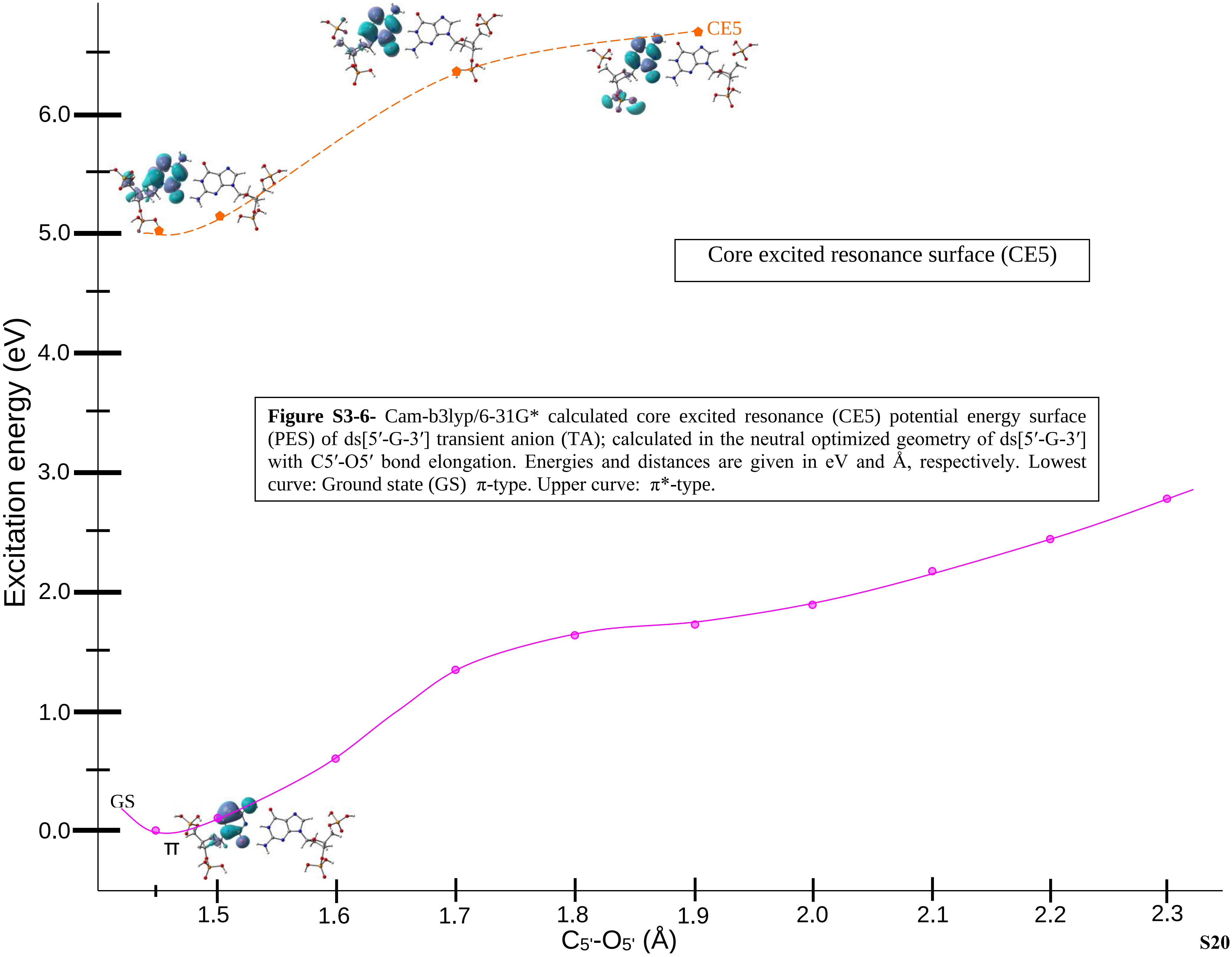

Figure S3-6- Cam-b3lyp/6-31G* calculated core excited resonance (CE5) potential energy surface (PES) of ds[5′-G-3′] transient anion (TA); calculated in the neutral optimized geometry of ds[5′-G-3′] with C5′-O5′ bond elongation. Energies and distances are given in eV and Å, respectively. Lowest curve: Ground state (GS) π-type. Upper curve: π*-type.

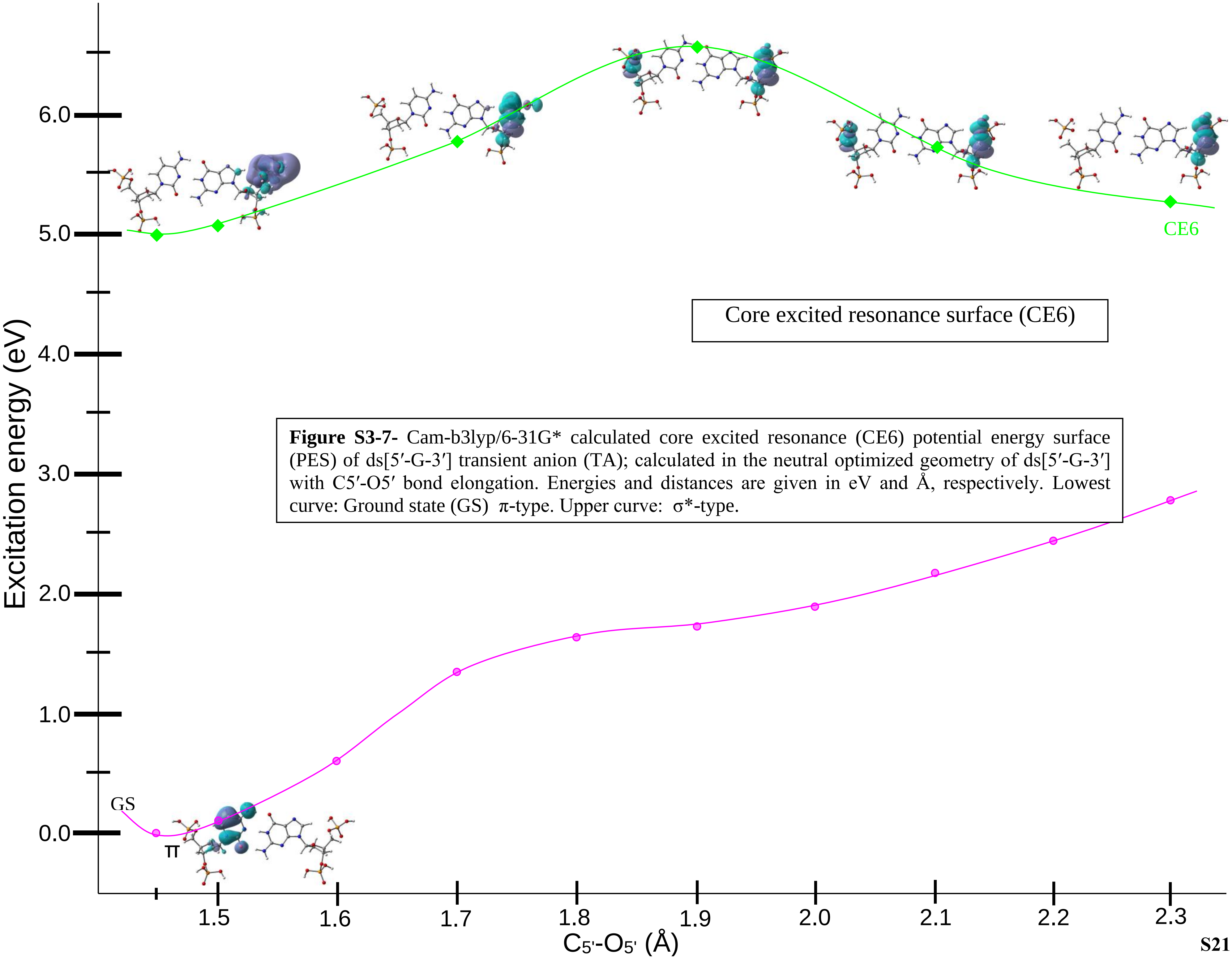

Figure S3-7- Cam-b3lyp/6-31G* calculated core excited resonance (CE6) potential energy surface (PES) of ds[5′-G-3′] transient anion (TA); calculated in the neutral optimized geometry of ds[5′-G-3′] with C5′-O5′ bond elongation. Energies and distances are given in eV and Å, respectively. Lowest curve: Ground state (GS) π-type. Upper curve: σ*-type.

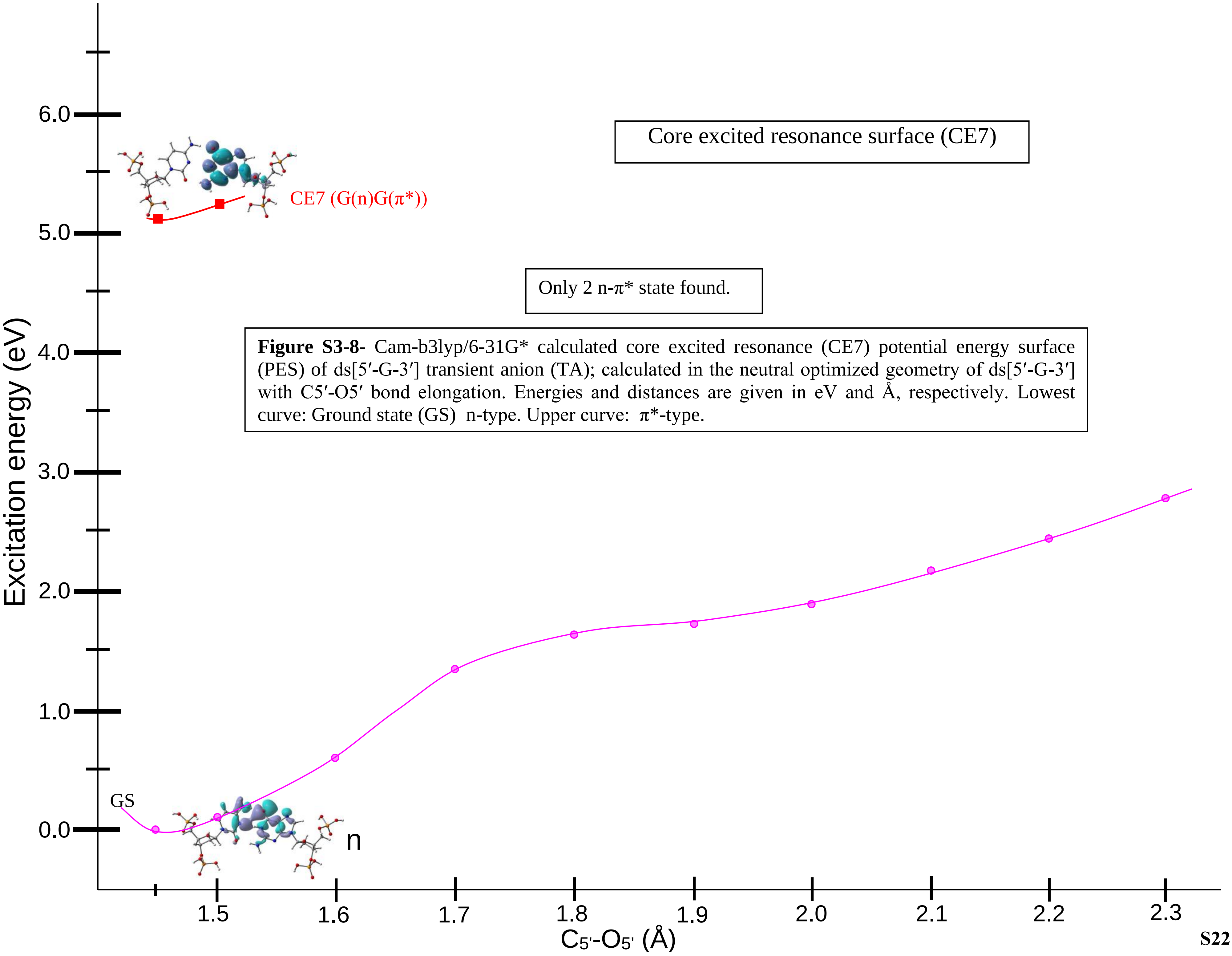

Figure S3-8- Cam-b3lyp/6-31G* calculated core excited resonance (CE7) potential energy surface (PES) of ds[5′-G-3′] transient anion (TA); calculated in the neutral optimized geometry of ds[5′-G-3′] with C5′-O5′ bond elongation. Energies and distances are given in eV and Å, respectively. Lowest curve: Ground state (GS) n-type. Upper curve: π*-type.

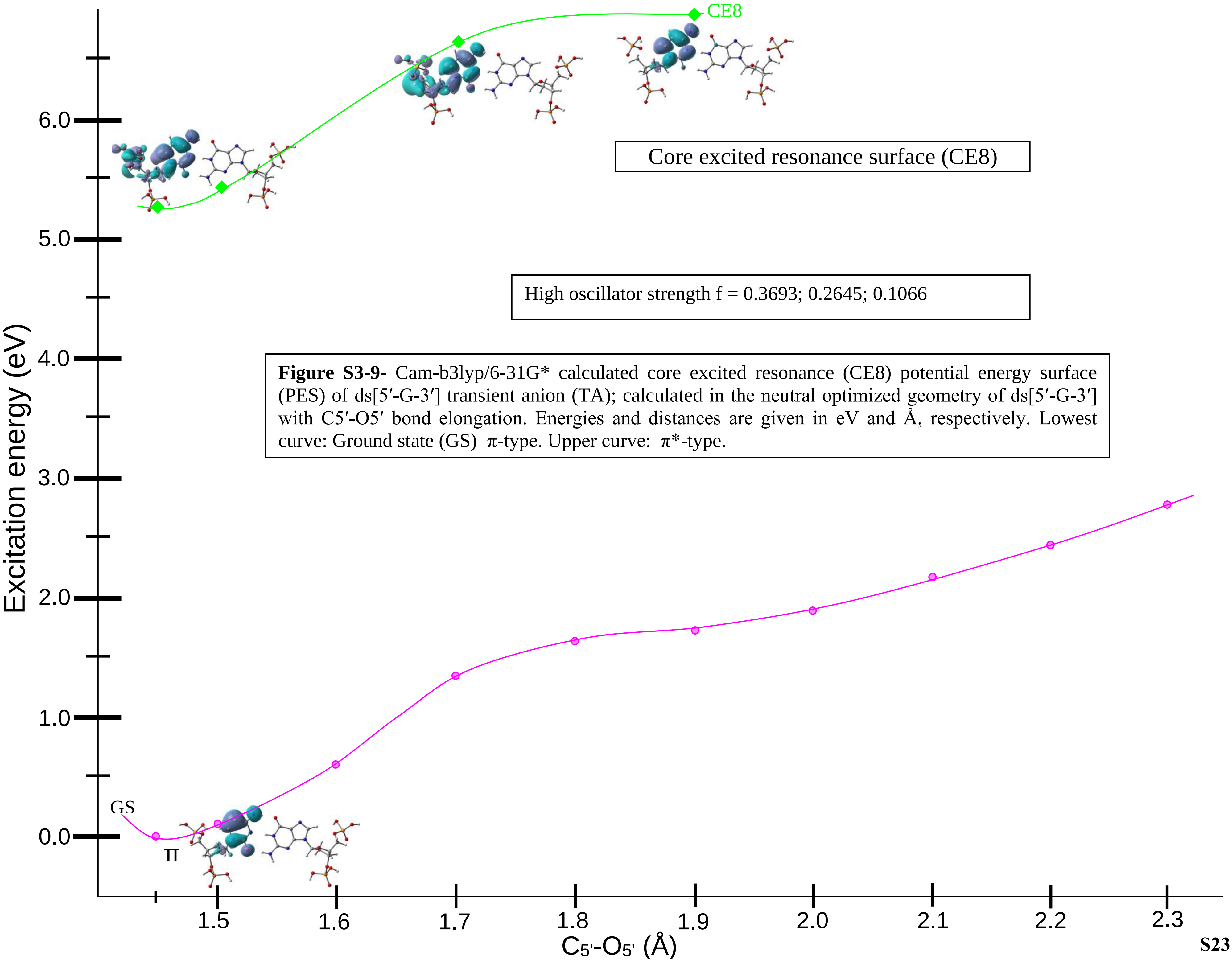

Figure S3-9- Cam-b3lyp/6-31G* calculated core excited resonance (CE8) potential energy surface (PES) of ds[5′-G-3′] transient anion (TA); calculated in the neutral optimized geometry of ds[5′-G-3′] with C5′-O5′ bond elongation. Energies and distances are given in eV and Å, respectively. Lowest curve: Ground state (GS) π-type. Upper curve: π*-type.

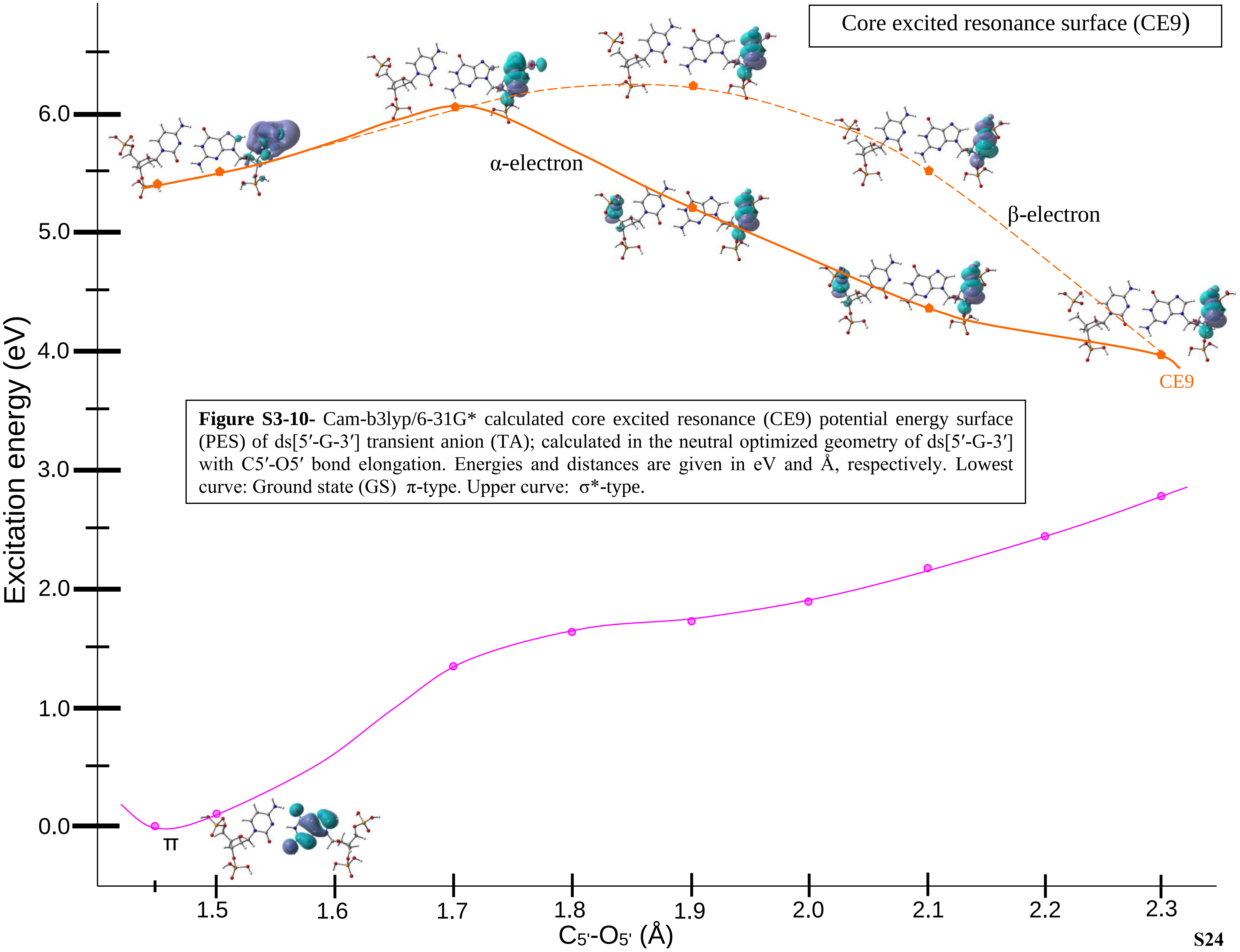

Figure S3-10- Cam-b3lyp/6-31G* calculated core excited resonance (CE9) potential energy surface (PES) of ds[5′-G-3′] transient anion (TA); calculated in the neutral optimized geometry of ds[5′-G-3′] with C5′-O5′ bond elongation. Energies and distances are given in eV and Å, respectively. Lowest curve: Ground state (GS) π-type. Upper curve: σ*-type.

Supporting Information 4

Transition energies with molecular orbitals (MOs) due to nine lowest core excited shape resonances (CE1 – CE9). Transition energies in eV are calculated at $C_{5'}$-$O_{5'}$ distance 1.44 Å

Figure S4-1- Cam-b3lyp/6-31G* calculated transition energies of ds[5′-G-3′] transient anion (TA); calculated in the neutral optimized geometry of ds[5′-G-3′] at $C_{5'}$-$O_{5'}$ distance 1.44 Å. Energies are given in eV, respectively. The 1st lowest core excited transitions (CE1) are taking place from the inner MOs lying below SOMO (MO number 211) to higher UMOs. Upward and downward arrows show the filled α- and β-electrons, respectively.

Figure S4-2- Cam-b3lyp/6-31G* calculated transition energies of ds[5′-G-3′] transient anion (TA); calculated in the neutral optimized geometry of ds[5′-G-3′] at $C_{5'}$-$O_{5'}$ distance 1.44 Å. Energies are given in eV, respectively. The 2nd lowest core excited transitions (CE2) are taking place from the inner MOs lying below SOMO (MO number 211) to higher UMOs. Upward and downward arrows show the filled α- and β-electrons, respectively.

Figure S4-3- Cam-b3lyp/6-31G* calculated transition energies of ds[5′-G-3′] transient anion (TA); calculated in the neutral optimized geometry of ds[5′-G-3′] at $C_{5'}$-$O_{5'}$ distance 1.44 Å. Energies are given in eV, respectively. The 3rd lowest core excited transitions (CE3) are taking place from the inner MOs lying below SOMO (MO number 211) to higher UMOs. Upward and downward arrows show the filled α- and β-electrons, respectively.

Figure S4-4- Cam-b3lyp/6-31G* calculated transition energies of ds[5′-G-3′] transient anion (TA); calculated in the neutral optimized geometry of ds[5′-G-3′] at $C_{5'}$-$O_{5'}$ distance 1.44 Å. Energies are given in eV, respectively. The 4th lowest core excited transitions (CE4) are taking place from the inner MOs lying below SOMO (MO number 211) to higher UMOs. Upward and downward arrows show the filled α- and β-electrons, respectively.

Figure S4-5- Cam-b3lyp/6-31G* calculated transition energies of ds[5′-G-3′] transient anion (TA); calculated in the neutral optimized geometry of ds[5′-G-3′] at $C_{5'}$-$O_{5'}$ distance 1.44 Å. Energies are given in eV, respectively. The 5th lowest core excited transitions (CE5) are taking place from the inner MOs lying below SOMO (MO number 211) to higher UMOs. Upward and downward arrows show the filled α- and β-electrons, respectively.

Figure S4-6- Cam-b3lyp/6-31G* calculated transition energies of ds[5′-G-3′] transient anion (TA); calculated in the neutral optimized geometry of ds[5′-G-3′] at $C_{5'}$-$O_{5'}$ distance 1.44 Å. Energies are given in eV, respectively. The 6th lowest core excited transitions (CE6) are taking place from the inner MOs lying below SOMO (MO number 211) to higher UMOs. Upward and downward arrows show the filled α- and β-electrons, respectively.

Figure S4-7- Cam-b3lyp/6-31G* calculated transition energies of ds[5′-G-3′] transient anion (TA); calculated in the neutral optimized geometry of ds[5′-G-3′] at $C_{5'}$-$O_{5'}$ distance 1.44 Å. Energies are given in eV, respectively. The 7th lowest core excited transitions (CE7) are taking place from the inner MOs lying below SOMO (MO number 211) to higher UMOs. Upward and downward arrows show the filled α- and β-electrons, respectively.

Figure S4-8- Cam-b3lyp/6-31G* calculated transition energies of ds[5′-G-3′] transient anion (TA); calculated in the neutral optimized geometry of ds[5′-G-3′] at $C_{5'}$-$O_{5'}$ distance 1.44 Å. Energies are given in eV, respectively. The 8th lowest core excited transitions (CE8) are taking place from the inner MOs lying below SOMO (MO number 211) to higher UMOs. Upward and downward arrows show the filled α- and β-electrons, respectively.

Figure S4-9- Cam-b3lyp/6-31G* calculated transition energies of ds[5′-G-3′] transient anion (TA); calculated in the neutral optimized geometry of ds[5′-G-3′] at $C_{5'}$-$O_{5'}$ distance 1.44 Å. Energies are given in eV, respectively. The 9th lowest core excited transitions (CE9) are taking place from the inner MOs lying below SOMO (MO number 211) to higher UMOs. Upward and downward arrows show the filled α- and β-electrons, respectively.

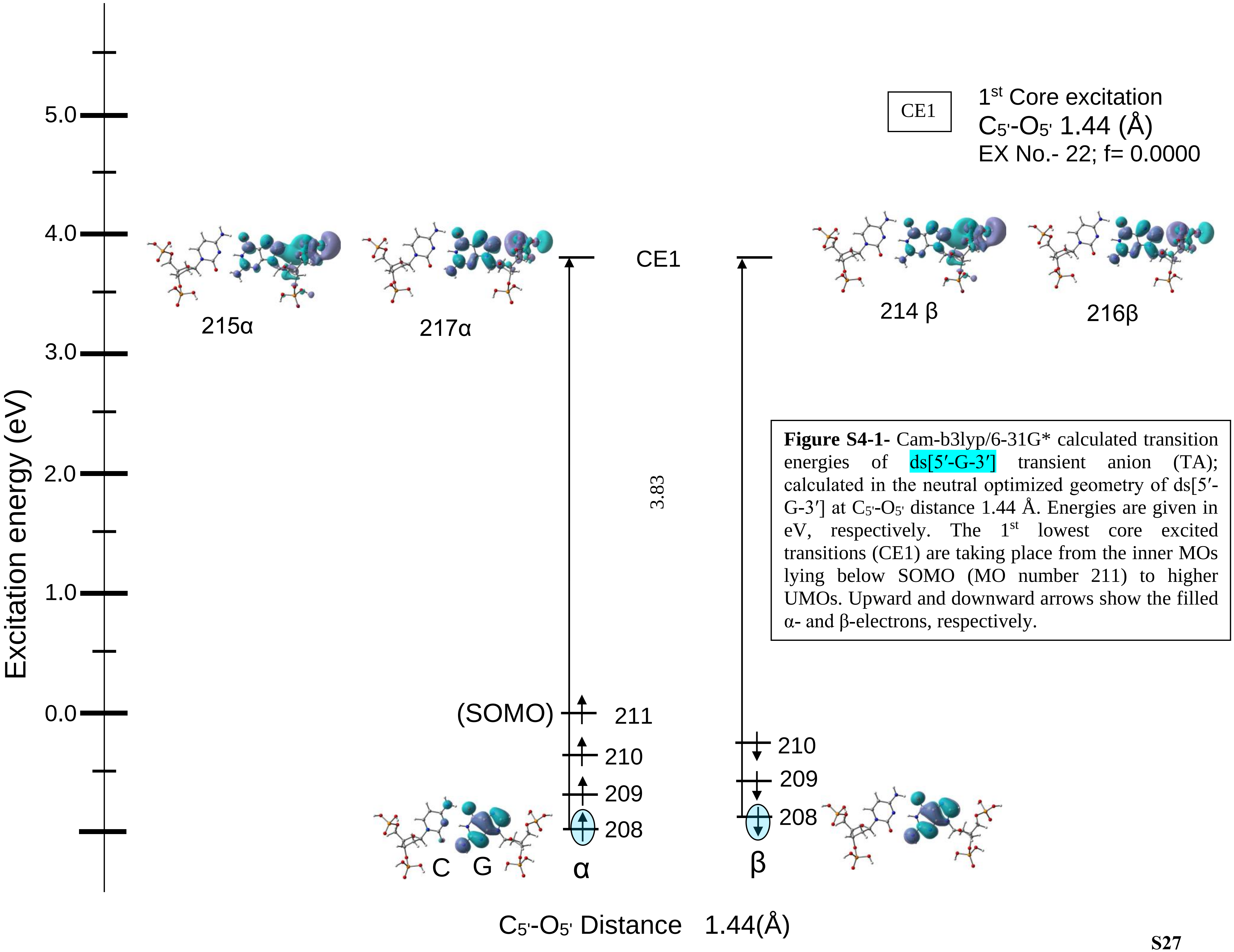

Figure S4-1- Cam-b3lyp/6-31G* calculated transition energies of ds[5′-G-3′] transient anion (TA); calculated in the neutral optimized geometry of ds[5′-G-3′] at $C_{5'}$-$O_{5'}$ distance 1.44 Å. Energies are given in eV, respectively. The 1st lowest core excited transitions (CE1) are taking place from the inner MOs lying below SOMO (MO number 211) to higher UMOs. Upward and downward arrows show the filled α- and β-electrons, respectively.

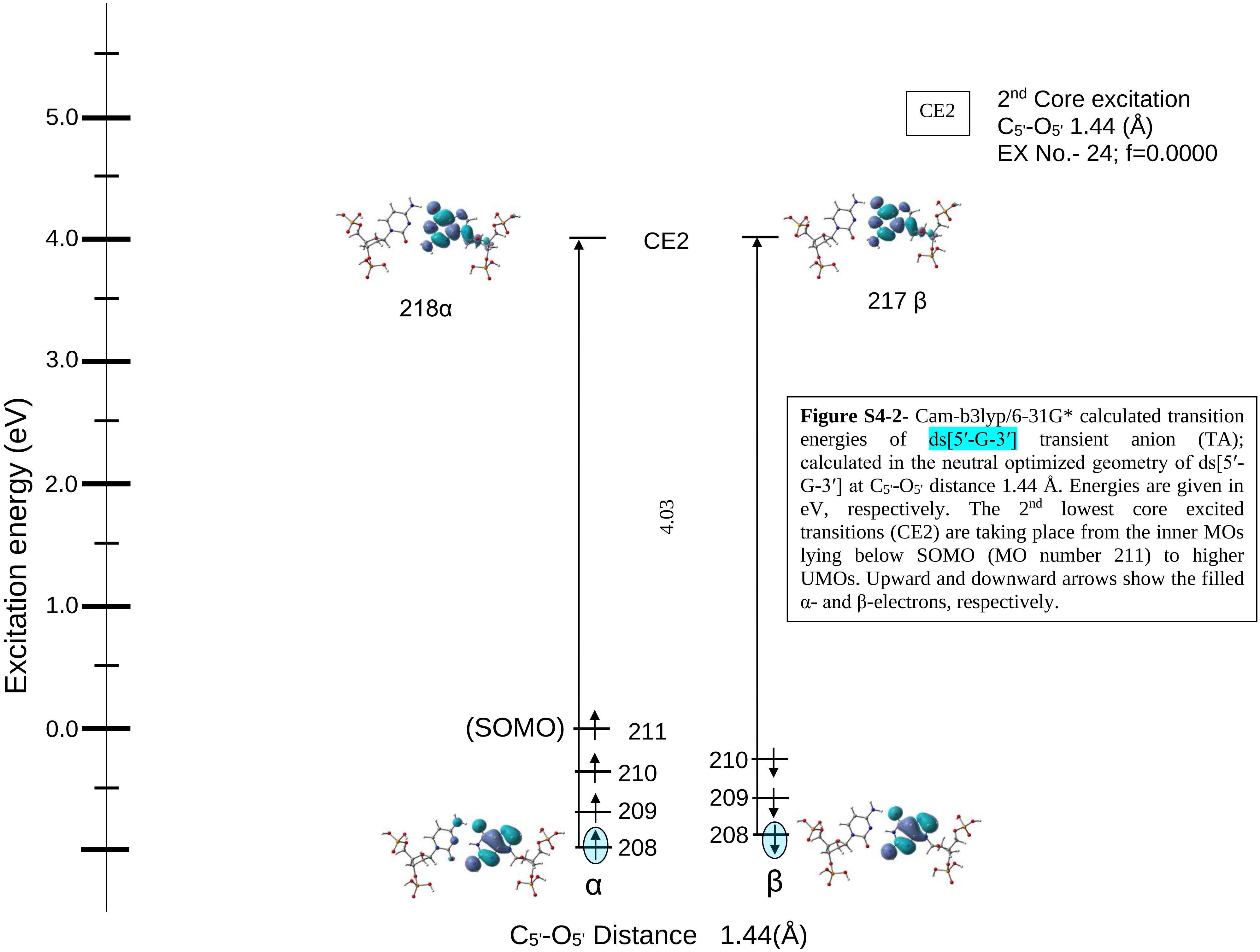

Figure S4-2- Cam-b3lyp/6-31G* calculated transition energies of ds[5′-G-3′] transient anion (TA); calculated in the neutral optimized geometry of ds[5′-G-3′] at $C_{5'}$-$O_{5'}$ distance 1.44 Å. Energies are given in eV, respectively. The 2nd lowest core excited transitions (CE2) are taking place from the inner MOs lying below SOMO (MO number 211) to higher UMOs. Upward and downward arrows show the filled α- and β-electrons, respectively.

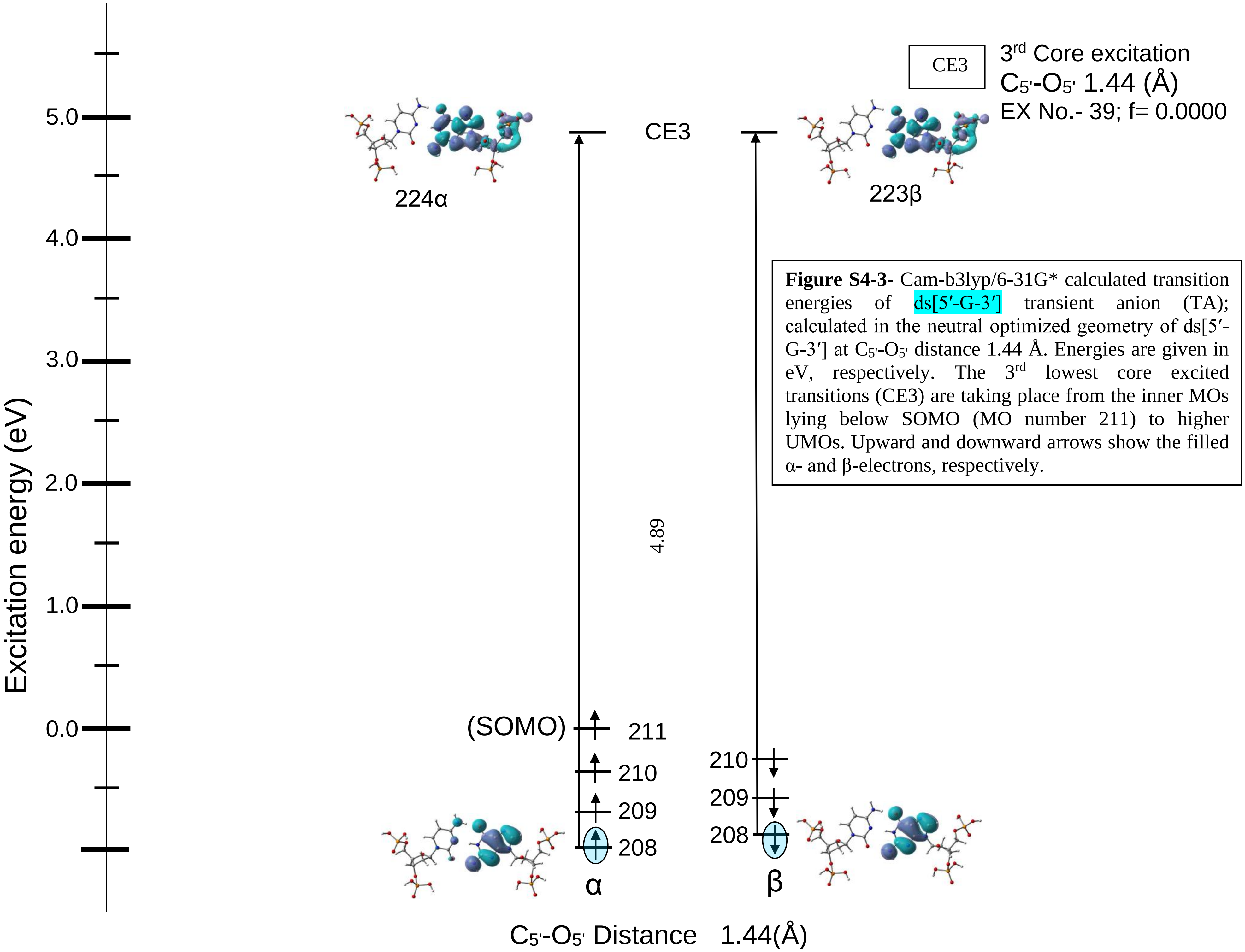

Figure S4-3- Cam-b3lyp/6-31G* calculated transition energies of ds[5′-G-3′] transient anion (TA); calculated in the neutral optimized geometry of ds[5′-G-3′] at $C_{5'}$-$O_{5'}$ distance 1.44 Å. Energies are given in eV, respectively. The 3rd lowest core excited transitions (CE3) are taking place from the inner MOs lying below SOMO (MO number 211) to higher UMOs. Upward and downward arrows show the filled α- and β-electrons, respectively.

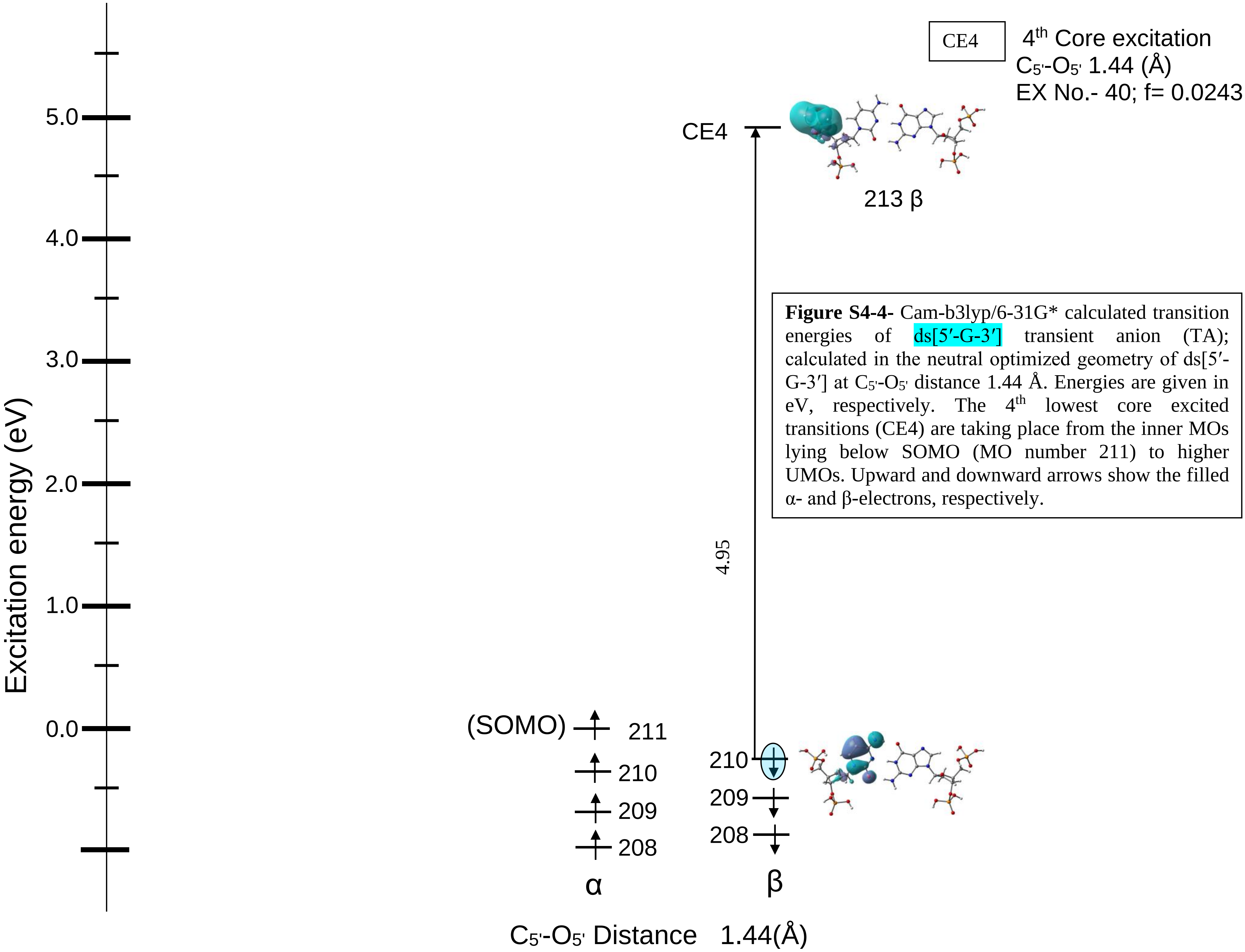

Figure S4-4- Cam-b3lyp/6-31G* calculated transition energies of ds[5′-G-3′] transient anion (TA); calculated in the neutral optimized geometry of ds[5′-G-3′] at $C_{5'}$-$O_{5'}$ distance 1.44 Å. Energies are given in eV, respectively. The 4th lowest core excited transitions (CE4) are taking place from the inner MOs lying below SOMO (MO number 211) to higher UMOs. Upward and downward arrows show the filled α- and β-electrons, respectively.

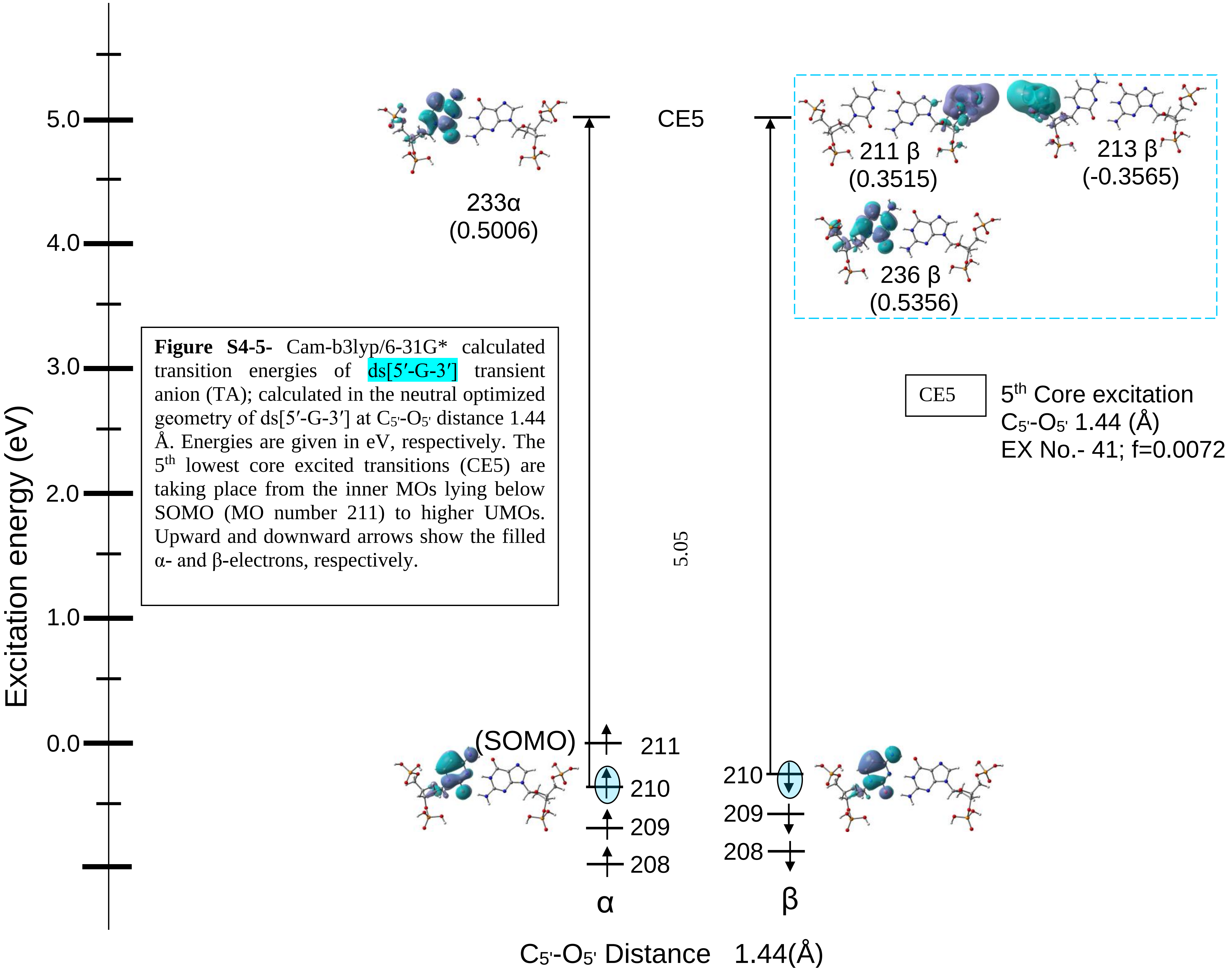

Figure S4-5- Cam-b3lyp/6-31G* calculated transition energies of ds[5′-G-3′] transient anion (TA); calculated in the neutral optimized geometry of ds[5′-G-3′] at $C_{5'}$-$O_{5'}$ distance 1.44 Å. Energies are given in eV, respectively. The 5th lowest core excited transitions (CE5) are taking place from the inner MOs lying below SOMO (MO number 211) to higher UMOs. Upward and downward arrows show the filled α- and β-electrons, respectively.

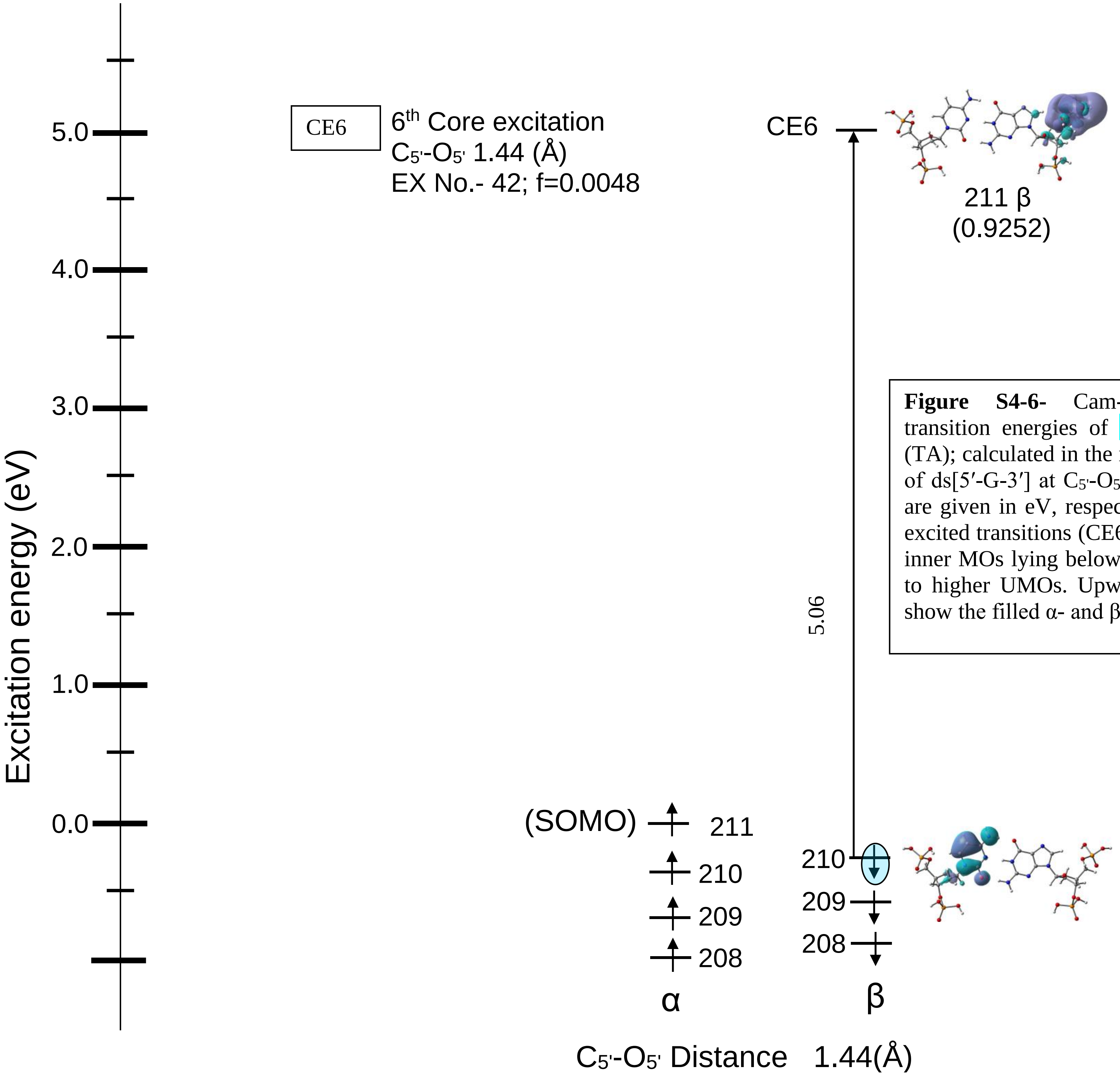

Figure S4-6- Cam-b3lyp/6-31G* calculated transition energies of ds[5′-G-3′] transient anion (TA); calculated in the neutral optimized geometry of ds[5′-G-3′] at $C_{5'}$-$O_{5'}$ distance 1.44 Å. Energies are given in eV, respectively. The 6th lowest core excited transitions (CE6) are taking place from the inner MOs lying below SOMO (MO number 211) to higher UMOs. Upward and downward arrows show the filled α- and β-electrons, respectively.

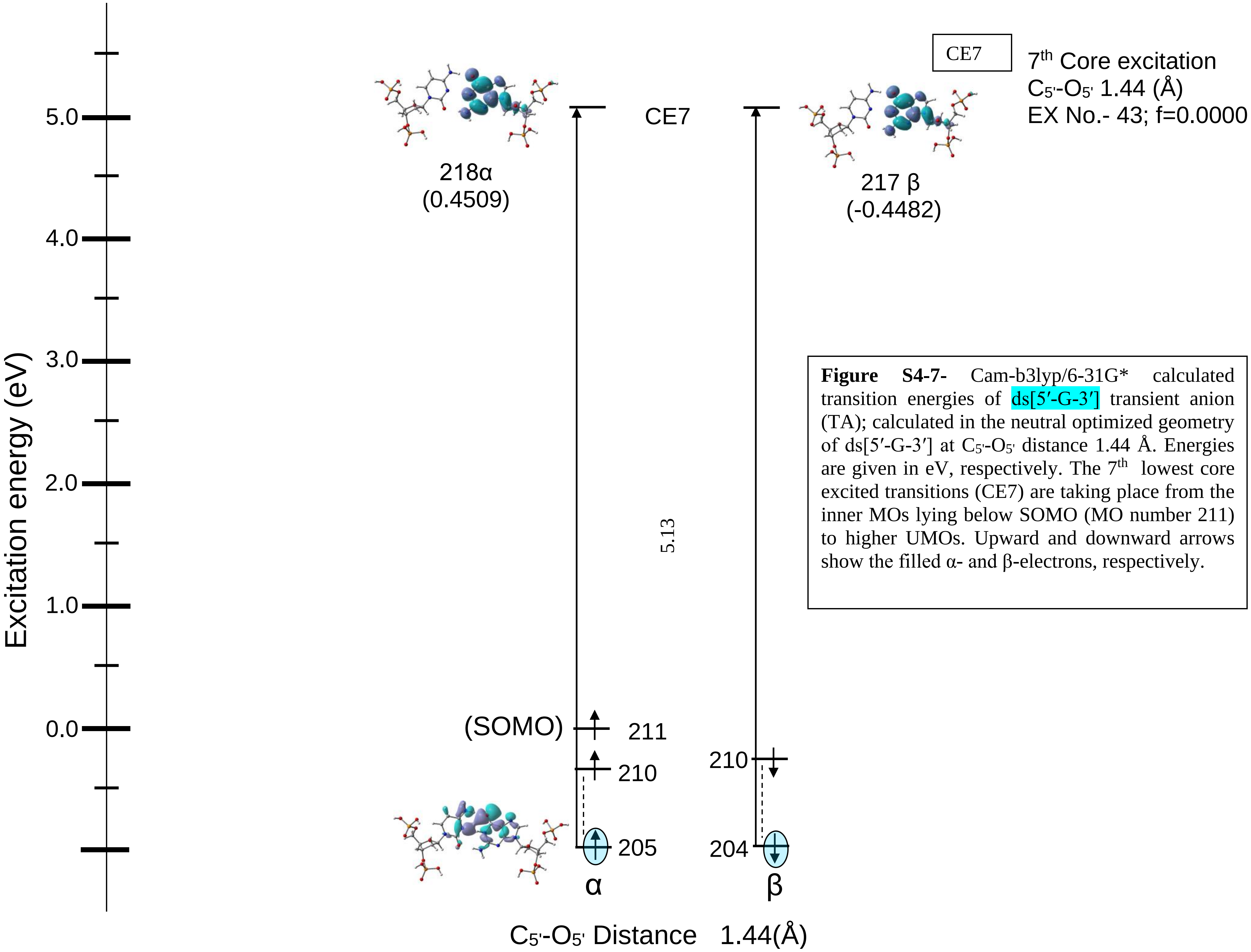

Figure S4-7- Cam-b3lyp/6-31G* calculated transition energies of ds[5′-G-3′] transient anion (TA); calculated in the neutral optimized geometry of ds[5′-G-3′] at $C_{5'}$-$O_{5'}$ distance 1.44 Å. Energies are given in eV, respectively. The 7th lowest core excited transitions (CE7) are taking place from the inner MOs lying below SOMO (MO number 211) to higher UMOs. Upward and downward arrows show the filled α- and β-electrons, respectively.

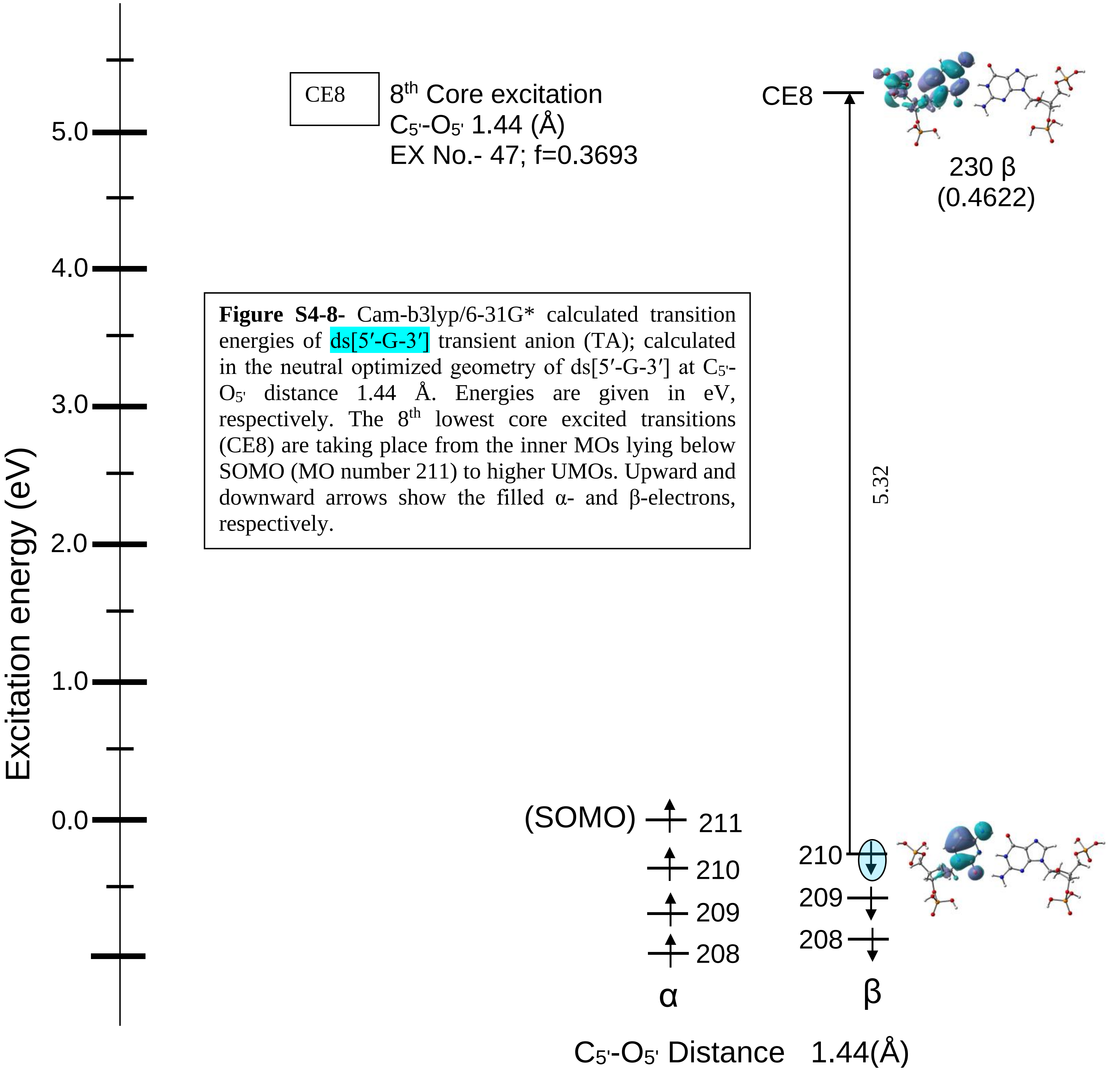

Figure S4-8- Cam-b3lyp/6-31G* calculated transition energies of ds[5′-G-3′] transient anion (TA); calculated in the neutral optimized geometry of ds[5′-G-3′] at $C_{5'}$-$O_{5'}$ distance 1.44 Å. Energies are given in eV, respectively. The 8th lowest core excited transitions (CE8) are taking place from the inner MOs lying below SOMO (MO number 211) to higher UMOs. Upward and downward arrows show the filled α- and β-electrons, respectively.

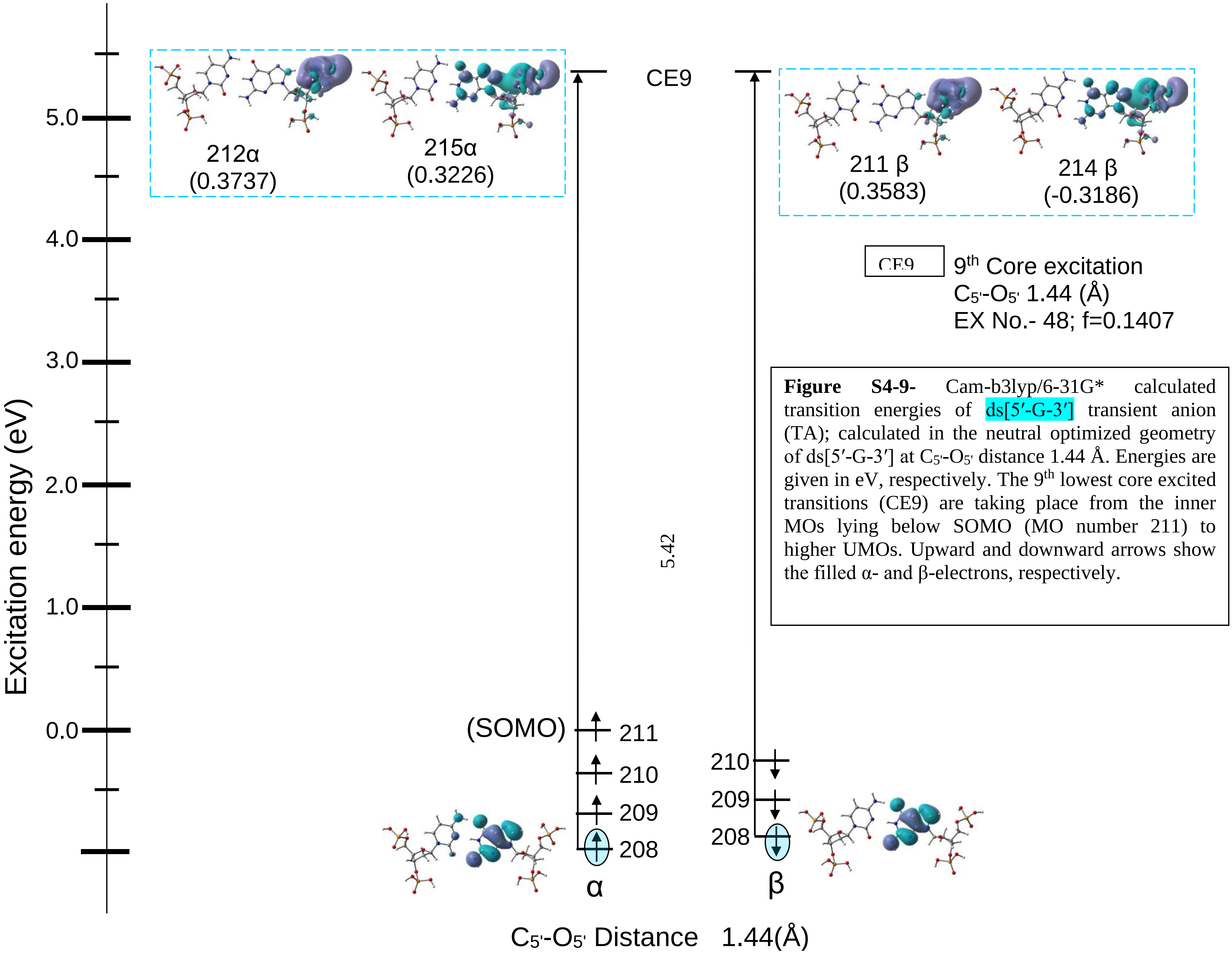

Figure S4-9- Cam-b3lyp/6-31G* calculated transition energies of ds[5′-G-3′] transient anion (TA); calculated in the neutral optimized geometry of ds[5′-G-3′] at $C_{5'}$-$O_{5'}$ distance 1.44 Å. Energies are given in eV, respectively. The 9th lowest core excited transitions (CE9) are taking place from the inner MOs lying below SOMO (MO number 211) to higher UMOs. Upward and downward arrows show the filled α- and β-electrons, respectively.

Supporting Information 5

Transition energies with molecular orbitals (MOs) due to nine lowest core excited shape resonances (CE1 – CE9). Transition energies in eV are calculated at $C_{5'}$-$O_{5'}$ distance 1.50 Å

Figure S5-1- Cam-b3lyp/6-31G* calculated transition energies of ds[5′-G-3′] transient anion (TA); calculated in the neutral optimized geometry of ds[5′-G-3′] at $C_{5'}$-$O_{5'}$ distance 1.50 Å. Energies are given in eV, respectively. The 1st lowest core excited transitions (CE1) are taking place from the inner MOs lying below SOMO (MO number 211) to higher UMOs. Upward and downward arrows show the filled α- and β-electrons, respectively.

Figure S5-2- Cam-b3lyp/6-31G* calculated transition energies of ds[5′-G-3′] transient anion (TA); calculated in the neutral optimized geometry of ds[5′-G-3′] at $C_{5'}$-$O_{5'}$ distance 1.50 Å. Energies are given in eV, respectively. The 2nd lowest core excited transitions (CE2) are taking place from the inner MOs lying below SOMO (MO number 211) to higher UMOs. Upward and downward arrows show the filled α- and β-electrons, respectively.

Figure S5-3- Cam-b3lyp/6-31G* calculated transition energies of ds[5′-G-3′] transient anion (TA); calculated in the neutral optimized geometry of ds[5′-G-3′] at $C_{5'}$-$O_{5'}$ distance 1.50 Å. Energies are given in eV, respectively. The 3rd lowest core excited transitions (CE3) are taking place from the inner MOs lying below SOMO (MO number 211) to higher UMOs. Upward and downward arrows show the filled α- and β-electrons, respectively.

Figure S5-4- Cam-b3lyp/6-31G* calculated transition energies of ds[5′-G-3′] transient anion (TA); calculated in the neutral optimized geometry of ds[5′-G-3′] at $C_{5'}$-$O_{5'}$ distance 1.50 Å. Energies are given in eV, respectively. The 4th lowest core excited transitions (CE4) are taking place from the inner MOs lying below SOMO (MO number 211) to higher UMOs. Upward and downward arrows show the filled α- and β-electrons, respectively.

Figure S5-5- Cam-b3lyp/6-31G* calculated transition energies of ds[5′-G-3′] transient anion (TA); calculated in the neutral optimized geometry of ds[5′-G-3′] at $C_{5'}$-$O_{5'}$ distance 1.50 Å. Energies are given in eV, respectively. The 5th lowest core excited transitions (CE5) are taking place from the inner MOs lying below SOMO (MO number 211) to higher UMOs. Upward and downward arrows show the filled α- and β-electrons, respectively.

Figure S5-6- Cam-b3lyp/6-31G* calculated transition energies of ds[5′-G-3′] transient anion (TA); calculated in the neutral optimized geometry of ds[5′-G-3′] at $C_{5'}$-$O_{5'}$ distance 1.50 Å. Energies are given in eV, respectively. The 6th lowest core excited transitions (CE6) are taking place from the inner MOs lying below SOMO (MO number 211) to higher UMOs. Upward and downward arrows show the filled α- and β-electrons, respectively.

Figure S5-7- Cam-b3lyp/6-31G* calculated transition energies of ds[5′-G-3′] transient anion (TA); calculated in the neutral optimized geometry of ds[5′-G-3′] at $C_{5'}$-$O_{5'}$ distance 1.50 Å. Energies are given in eV, respectively. The 7th lowest core excited transitions (CE7) are taking place from the inner MOs lying below SOMO (MO number 211) to higher UMOs. Upward and downward arrows show the filled α- and β-electrons, respectively.

Figure S5-8- Cam-b3lyp/6-31G* calculated transition energies of ds[5′-G-3′] transient anion (TA); calculated in the neutral optimized geometry of ds[5′-G-3′] at $C_{5'}$-$O_{5'}$ distance 1.50 Å. Energies are given in eV, respectively. The 9th lowest core excited transitions (CE9) are taking place from the inner MOs lying below SOMO (MO number 211) to higher UMOs. Upward and downward arrows show the filled α- and β-electrons, respectively.

Figure S5-9- Cam-b3lyp/6-31G* calculated transition energies of ds[5′-G-3′] transient anion (TA); calculated in the neutral optimized geometry of ds[5′-G-3′] at $C_{5'}$-$O_{5'}$ distance 1.50 Å. Energies are given in eV, respectively. The 10th lowest core excited transitions (CE10) are taking place from the inner MOs lying below SOMO (MO number 211) to higher UMOs. Upward and downward arrows show the filled α- and β-electrons, respectively.

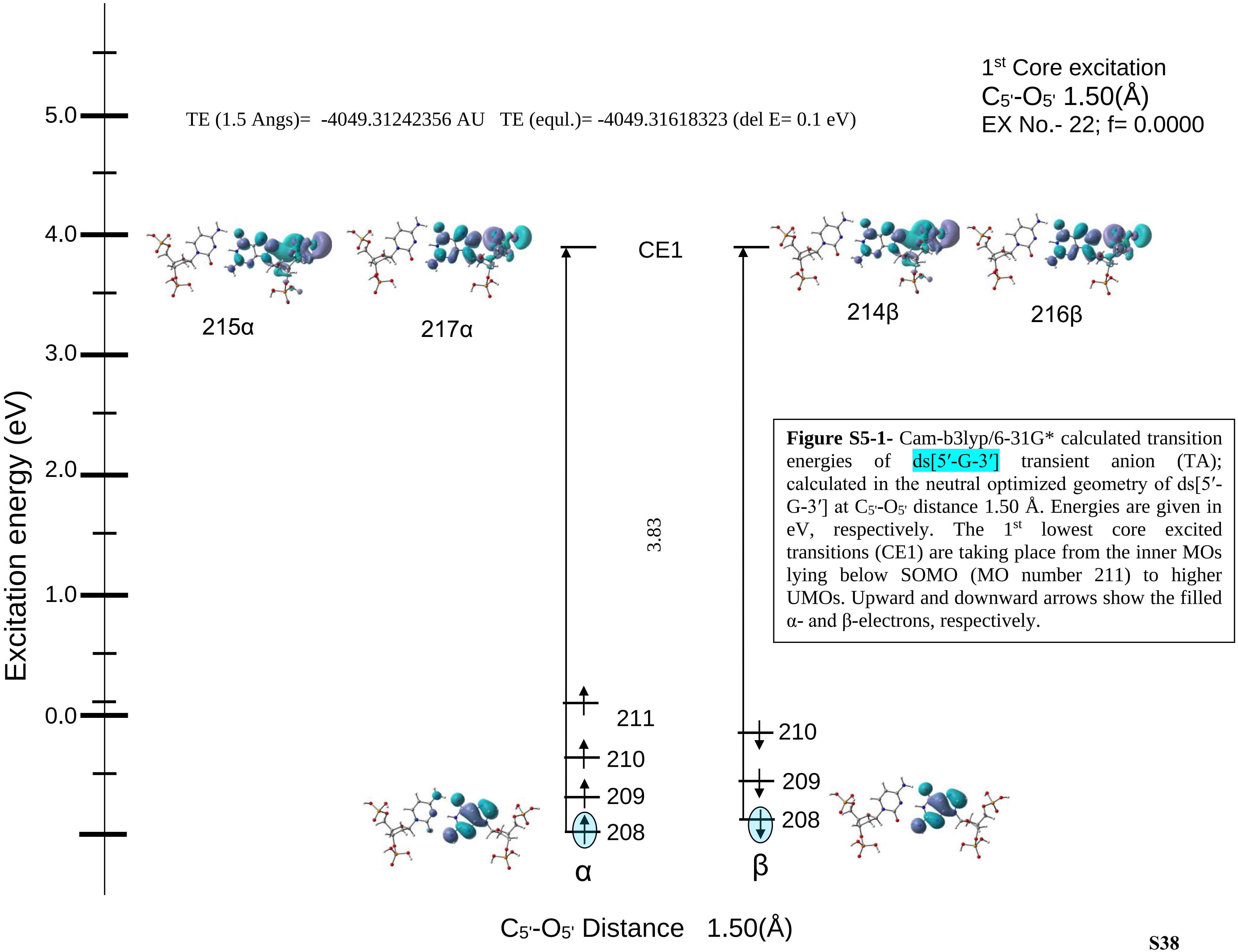

Figure S5-1- Cam-b3lyp/6-31G* calculated transition energies of ds[5′-G-3′] transient anion (TA); calculated in the neutral optimized geometry of ds[5′-G-3′] at $C_{5'}$-$O_{5'}$ distance 1.50 Å. Energies are given in eV, respectively. The 1st lowest core excited transitions (CE1) are taking place from the inner MOs lying below SOMO (MO number 211) to higher UMOs. Upward and downward arrows show the filled α- and β-electrons, respectively.

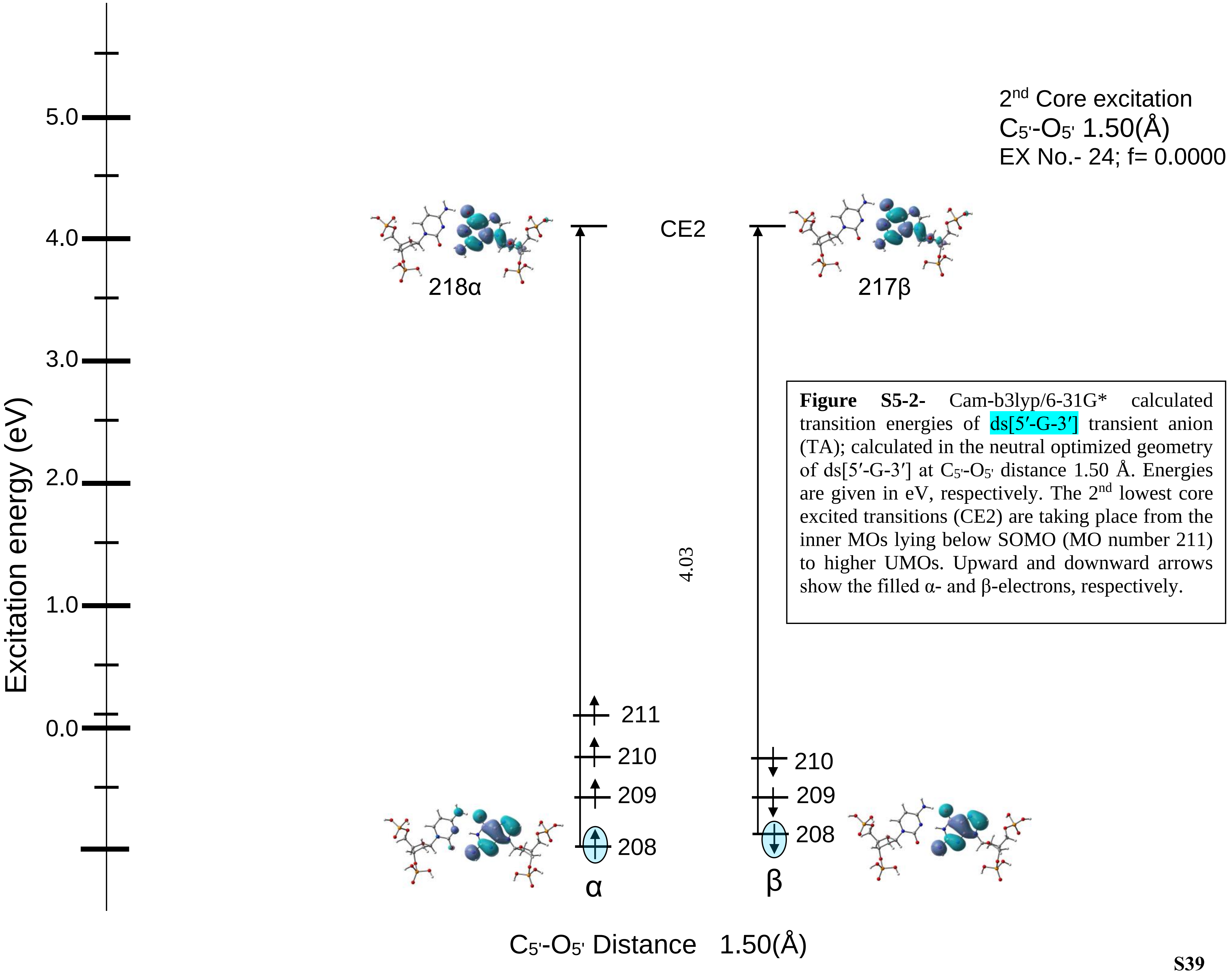

Figure S5-2- Cam-b3lyp/6-31G* calculated transition energies of ds[5′-G-3′] transient anion (TA); calculated in the neutral optimized geometry of ds[5′-G-3′] at $C_{5'}$-$O_{5'}$ distance 1.50 Å. Energies are given in eV, respectively. The 2nd lowest core excited transitions (CE2) are taking place from the inner MOs lying below SOMO (MO number 211) to higher UMOs. Upward and downward arrows show the filled α- and β-electrons, respectively.

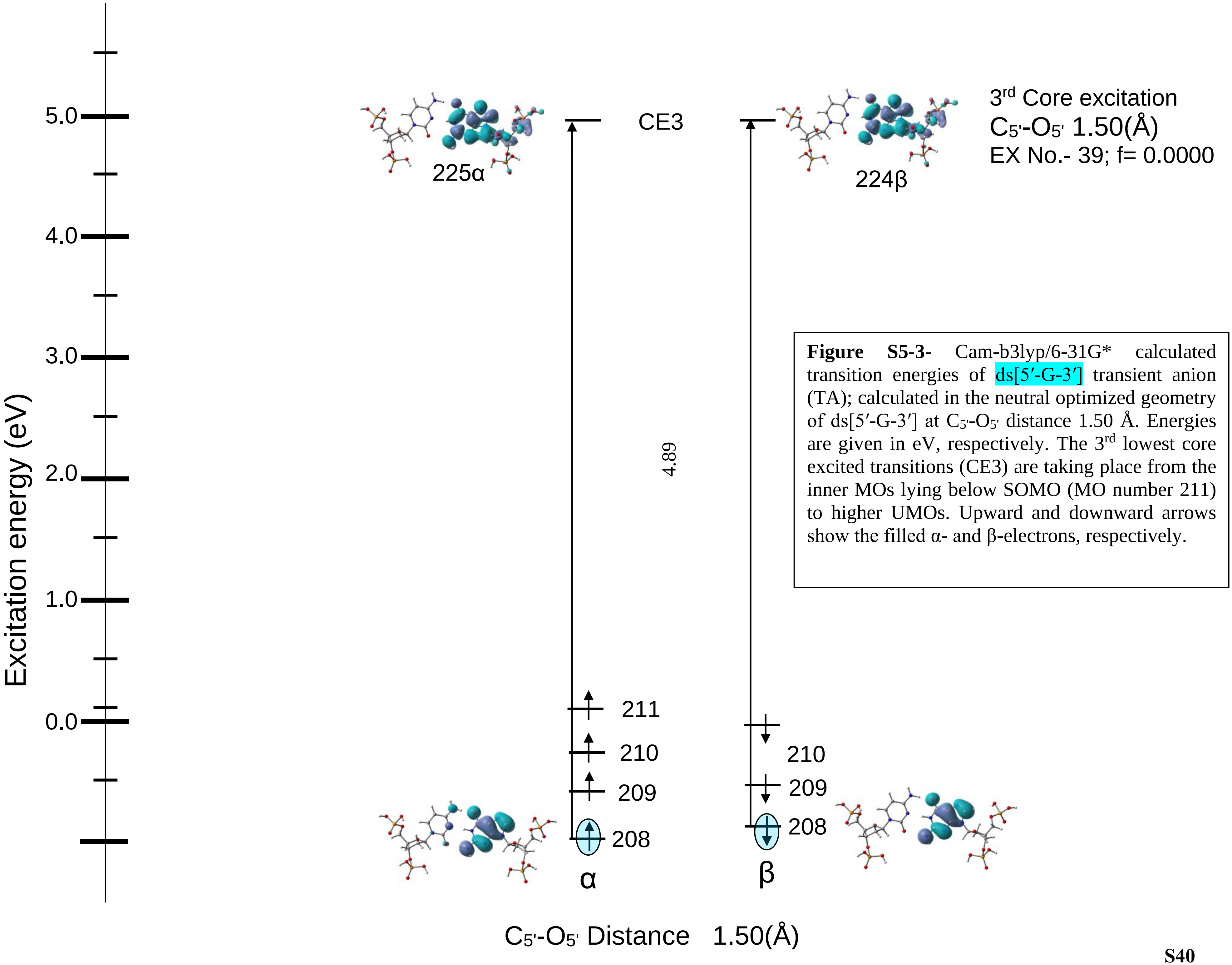

Figure S5-3- Cam-b3lyp/6-31G* calculated transition energies of ds[5′-G-3′] transient anion (TA); calculated in the neutral optimized geometry of ds[5′-G-3′] at $C_{5'}$-$O_{5'}$ distance 1.50 Å. Energies are given in eV, respectively. The 3rd lowest core excited transitions (CE3) are taking place from the inner MOs lying below SOMO (MO number 211) to higher UMOs. Upward and downward arrows show the filled α- and β-electrons, respectively.

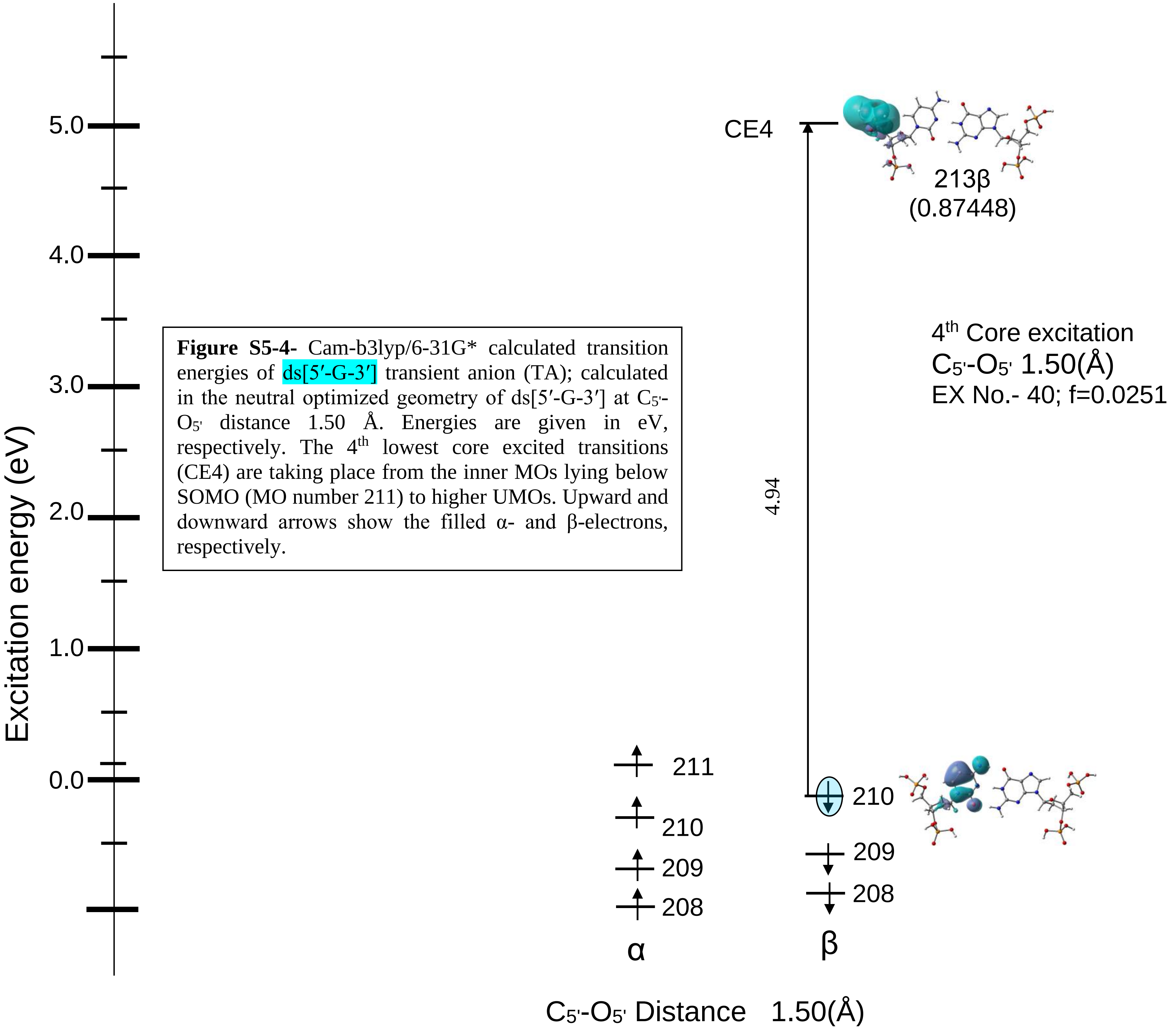

Figure S5-4- Cam-b3lyp/6-31G* calculated transition energies of ds[5′-G-3′] transient anion (TA); calculated in the neutral optimized geometry of ds[5′-G-3′] at $C_{5'}$-$O_{5'}$ distance 1.50 Å. Energies are given in eV, respectively. The 4th lowest core excited transitions (CE4) are taking place from the inner MOs lying below SOMO (MO number 211) to higher UMOs. Upward and downward arrows show the filled α- and β-electrons, respectively.

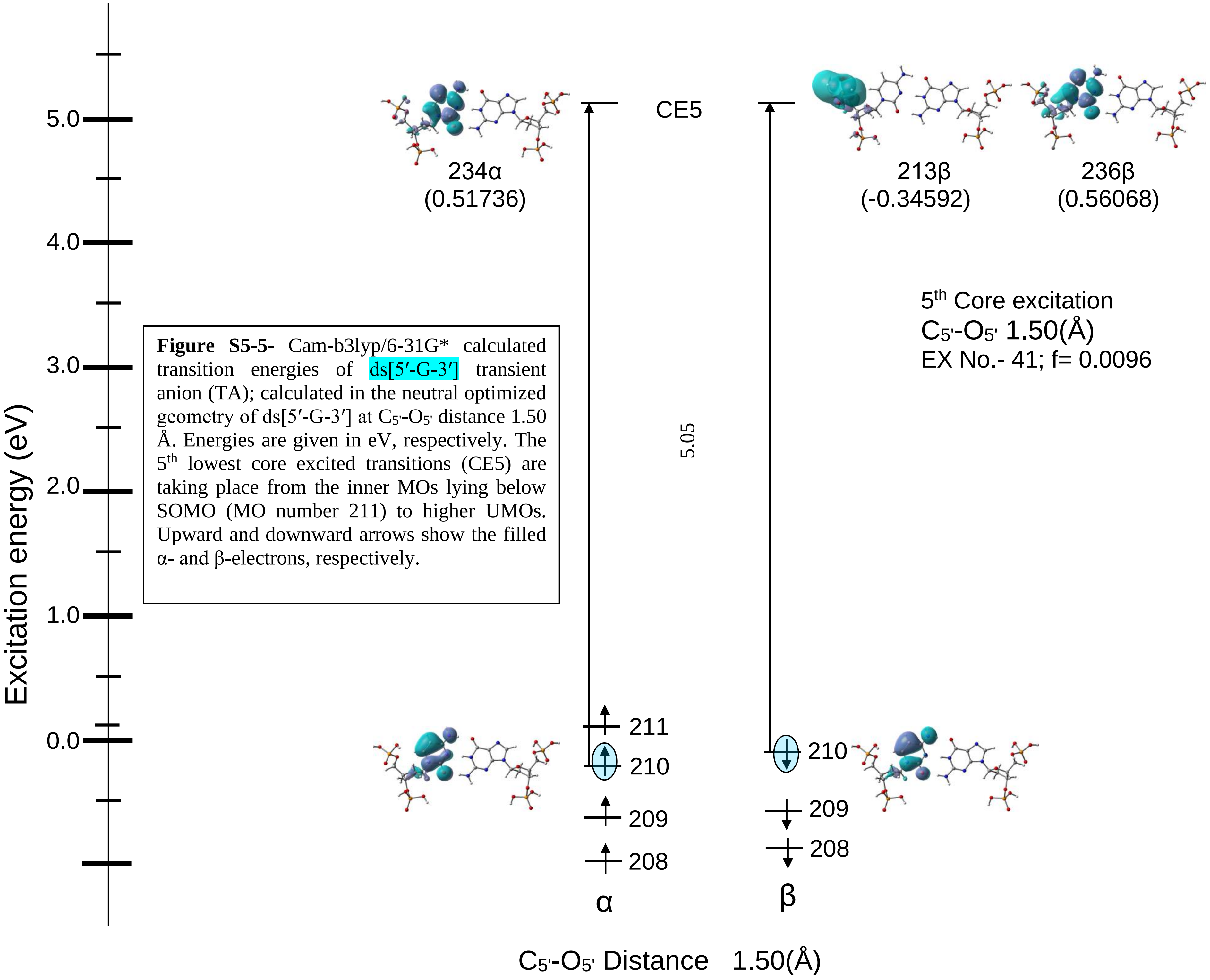

Figure S5-5- Cam-b3lyp/6-31G* calculated transition energies of ds[5′-G-3′] transient anion (TA); calculated in the neutral optimized geometry of ds[5′-G-3′] at $C_{5'}$-$O_{5'}$ distance 1.50 Å. Energies are given in eV, respectively. The 5th lowest core excited transitions (CE5) are taking place from the inner MOs lying below SOMO (MO number 211) to higher UMOs. Upward and downward arrows show the filled α- and β-electrons, respectively.

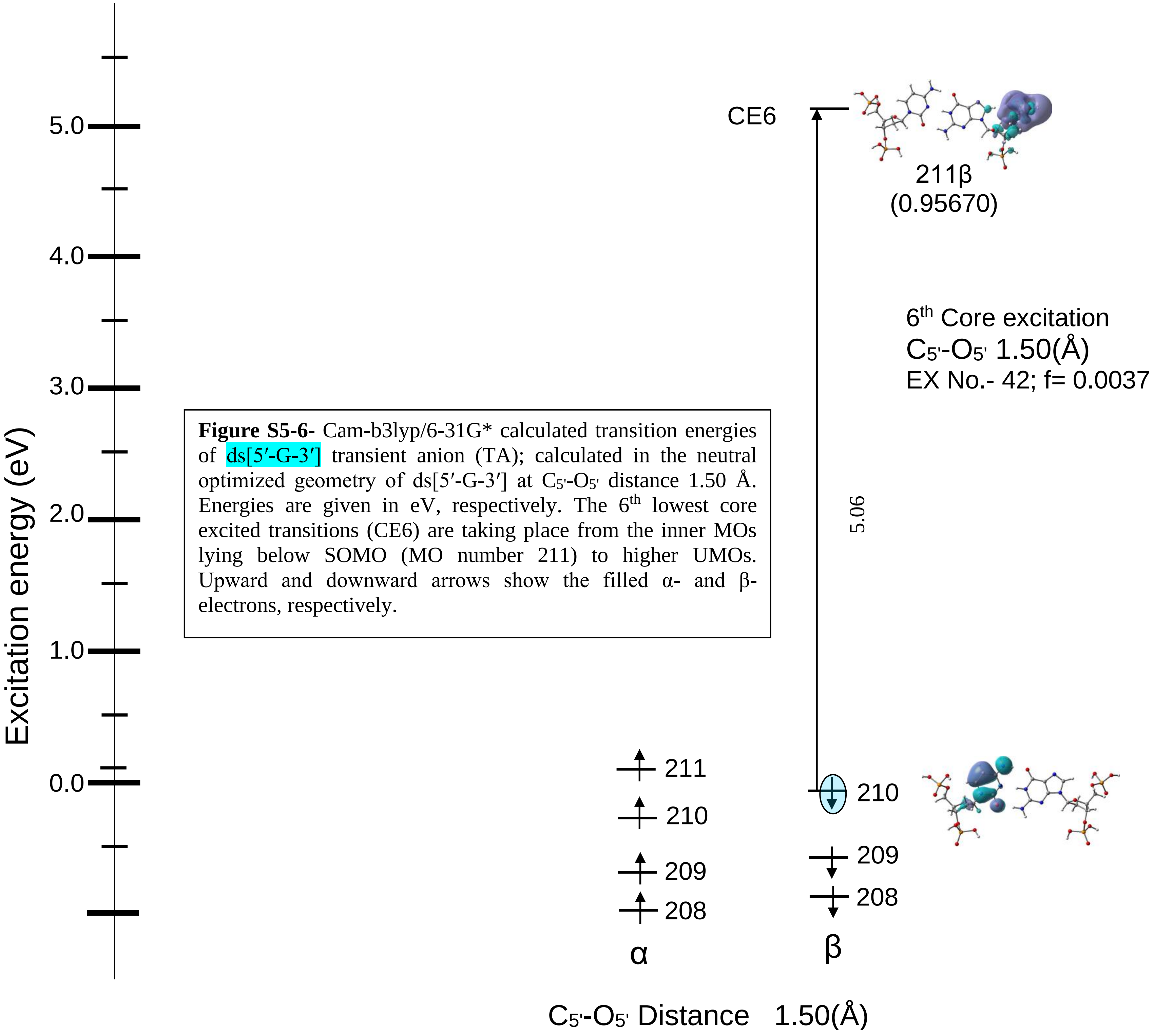

Figure S5-6- Cam-b3lyp/6-31G* calculated transition energies of ds[5′-G-3′] transient anion (TA); calculated in the neutral optimized geometry of ds[5′-G-3′] at $C_{5'}$-$O_{5'}$ distance 1.50 Å. Energies are given in eV, respectively. The 6th lowest core excited transitions (CE6) are taking place from the inner MOs lying below SOMO (MO number 211) to higher UMOs. Upward and downward arrows show the filled α- and β-electrons, respectively.

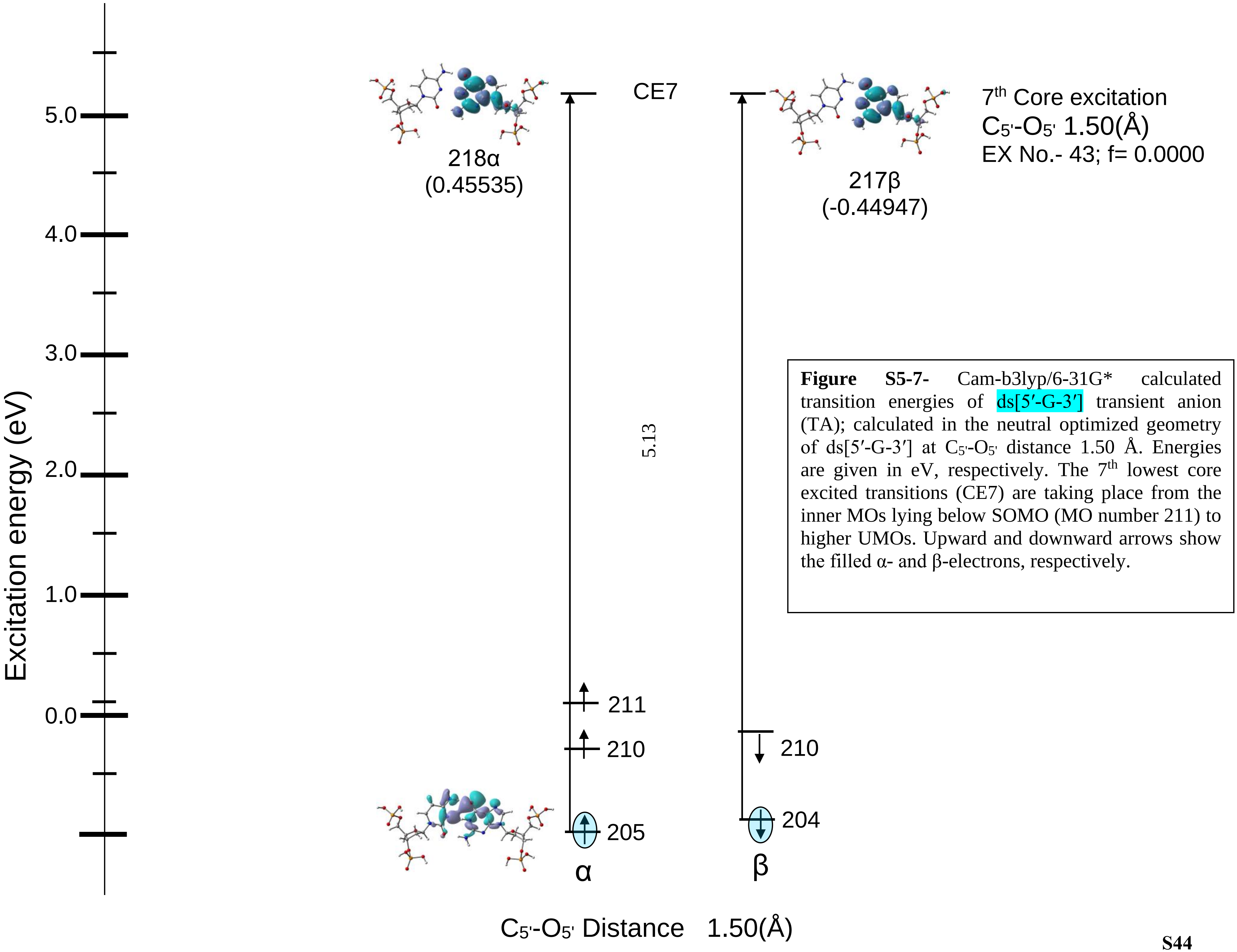

Figure S5-7- Cam-b3lyp/6-31G* calculated transition energies of ds[5′-G-3′] transient anion (TA); calculated in the neutral optimized geometry of ds[5′-G-3′] at $C_{5'}$-$O_{5'}$ distance 1.50 Å. Energies are given in eV, respectively. The 7th lowest core excited transitions (CE7) are taking place from the inner MOs lying below SOMO (MO number 211) to higher UMOs. Upward and downward arrows show the filled α- and β-electrons, respectively.

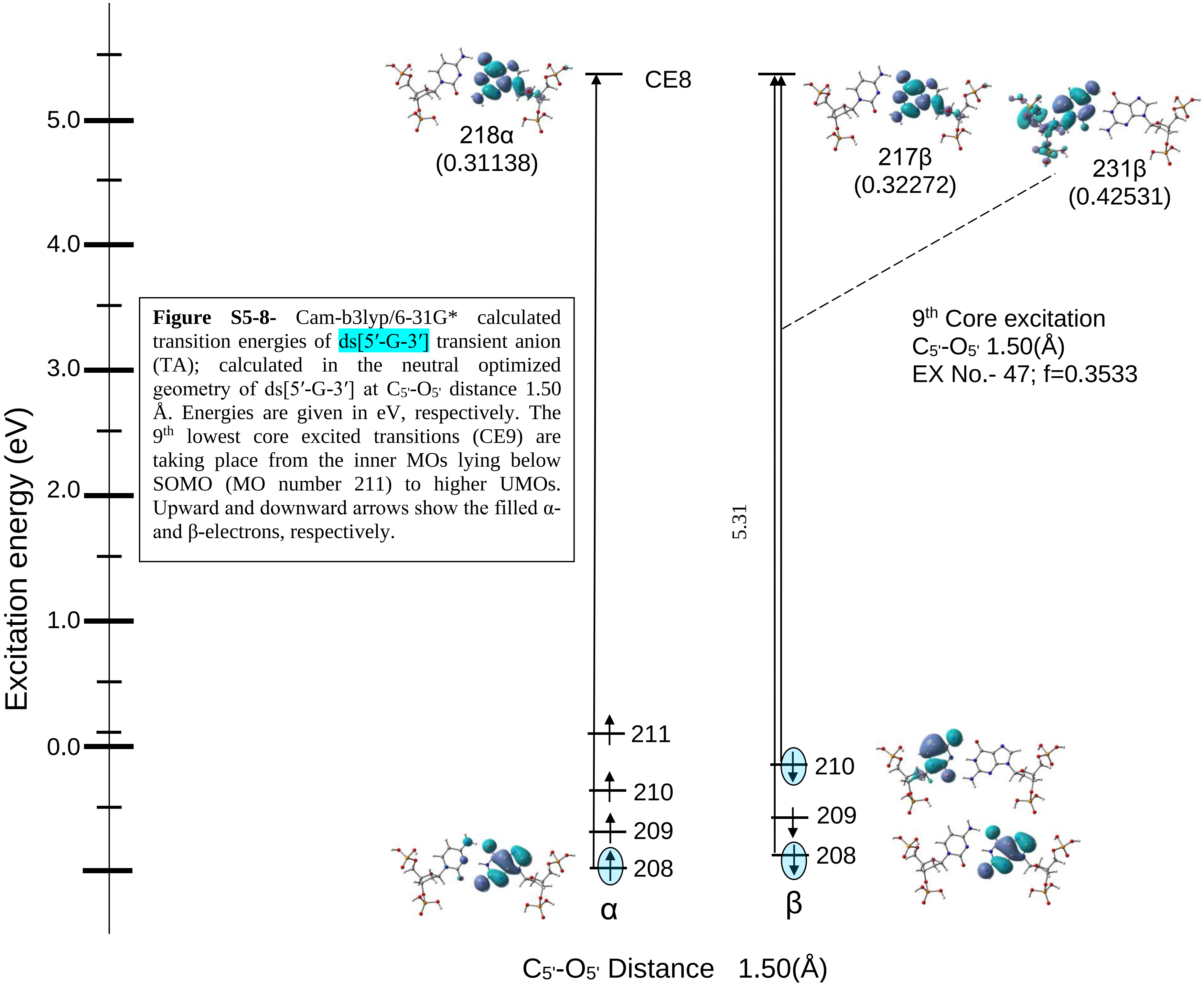

Figure S5-8- Cam-b3lyp/6-31G* calculated transition energies of ds[5′-G-3′] transient anion (TA); calculated in the neutral optimized geometry of ds[5′-G-3′] at $C_{5'}$-$O_{5'}$ distance 1.50 Å. Energies are given in eV, respectively. The 9th lowest core excited transitions (CE9) are taking place from the inner MOs lying below SOMO (MO number 211) to higher UMOs. Upward and downward arrows show the filled α- and β-electrons, respectively.

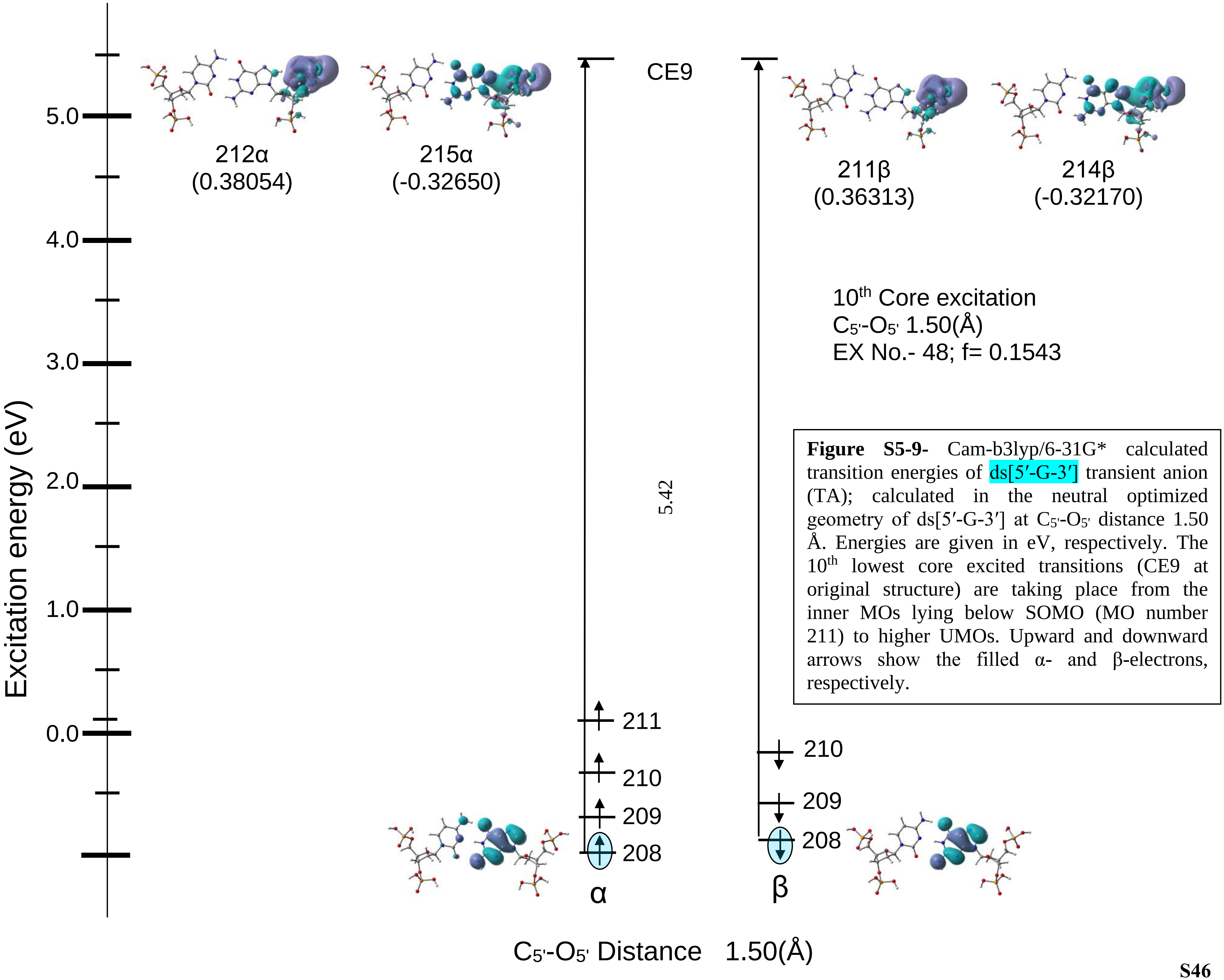

Figure S5-9- Cam-b3lyp/6-31G* calculated transition energies of ds[5′-G-3′] transient anion (TA); calculated in the neutral optimized geometry of ds[5′-G-3′] at $C_{5'}$-$O_{5'}$ distance 1.50 Å. Energies are given in eV, respectively. The 10th lowest core excited transitions (CE9 at original structure) are taking place from the inner MOs lying below SOMO (MO number 211) to higher UMOs. Upward and downward arrows show the filled α- and β-electrons, respectively.

Supporting Information 6

Transition energies with molecular orbitals (MOs) due to nine lowest core excited shape resonances (CE1 – CE9). Transition energies in eV are calculated at $C_{5'}$-$O_{5'}$ distance 1.70 Å

Figure S6-1- Cam-b3lyp/6-31G* calculated transition energies of ds[5′-G-3′] transient anion (TA); calculated in the neutral optimized geometry of ds[5′-G-3′] at $C_{5'}$-$O_{5'}$ distance 1.70 Å. Energies are given in eV, respectively. The 1st lowest core excited transitions (CE1) are taking place from the inner MOs lying below SOMO (MO number 211) to higher UMOs. Upward and downward arrows show the filled α- and β-electrons, respectively. (CE1)

Figure S6-2- Cam-b3lyp/6-31G* calculated transition energies of ds[5′-G-3′] transient anion (TA); calculated in the neutral optimized geometry of ds[5′-G-3′] at $C_{5'}$-$O_{5'}$ distance 1.70 Å. Energies are given in eV, respectively. The 2nd lowest core excited transitions (CE2) are taking place from the inner MOs lying below SOMO (MO number 211) to higher UMOs. Upward and downward arrows show the filled α- and β-electrons, respectively. (CE2)

Figure S6-3- Cam-b3lyp/6-31G* calculated transition energies of ds[5′-G-3′] transient anion (TA); calculated in the neutral optimized geometry of ds[5′-G-3′] at $C_{5'}$-$O_{5'}$ distance 1.70 Å. Energies are given in eV, respectively. The 3rd lowest core excited transitions (CE3) are taking place from the inner MOs lying below SOMO (MO number 211) to higher UMOs. Upward and downward arrows show the filled α- and β-electrons, respectively. (CE6)

Figure S6-4- Cam-b3lyp/6-31G* calculated transition energies of ds[5′-G-3′] transient anion (TA); calculated in the neutral optimized geometry of ds[5′-G-3′] at $C_{5'}$-$O_{5'}$ distance 1.70 Å. Energies are given in eV, respectively. The 4th lowest core excited transitions (CE4) are taking place from the inner MOs lying below SOMO (MO number 211) to higher UMOs. Upward and downward arrows show the filled α- and β-electrons, respectively. (CE9)

Figure S6-5- Cam-b3lyp/6-31G* calculated transition energies of ds[5′-G-3′] transient anion (TA); calculated in the neutral optimized geometry of ds[5′-G-3′] at $C_{5'}$-$O_{5'}$ distance 1.70 Å. Energies are given in eV, respectively. The 5th lowest core excited transitions (CE5) are taking place from the inner MOs lying below SOMO (MO number 211) to higher UMOs. Upward and downward arrows show the filled α- and β-electrons, respectively. (CE4)

Figure S6-6- Cam-b3lyp/6-31G* calculated transition energies of ds[5′-G-3′] transient anion (TA); calculated in the neutral optimized geometry of ds[5′-G-3′] at $C_{5'}$-$O_{5'}$ distance 1.70 Å. Energies are given in eV, respectively. The 6th lowest core excited transitions (CE6) are taking place from the inner MOs lying below SOMO (MO number 211) to higher UMOs. Upward and downward arrows show the filled α- and β-electrons, respectively. (CE3)

Figure S6-7- Cam-b3lyp/6-31G* calculated transition energies of ds[5′-G-3′] transient anion (TA); calculated in the neutral optimized geometry of ds[5′-G-3′] at $C_{5'}$-$O_{5'}$ distance 1.70 Å. Energies are given in eV, respectively. The 7th lowest core excited transitions (CE7) are taking place from the inner MOs lying below SOMO (MO number 211) to higher UMOs. Upward and downward arrows show the filled α- and β-electrons, respectively. (CE5)

Figure S6-8- Cam-b3lyp/6-31G* calculated transition energies of ds[5′-G-3′] transient anion (TA); calculated in the neutral optimized geometry of ds[5′-G-3′] at $C_{5'}$-$O_{5'}$ distance 1.70 Å. Energies are given in eV, respectively. The 8th core excited transitions (CE8) are taking place from the inner MOs lying below SOMO (MO number 211) to higher UMOs. Upward and downward arrows show the filled α- and β-electrons, respectively. (CE8)

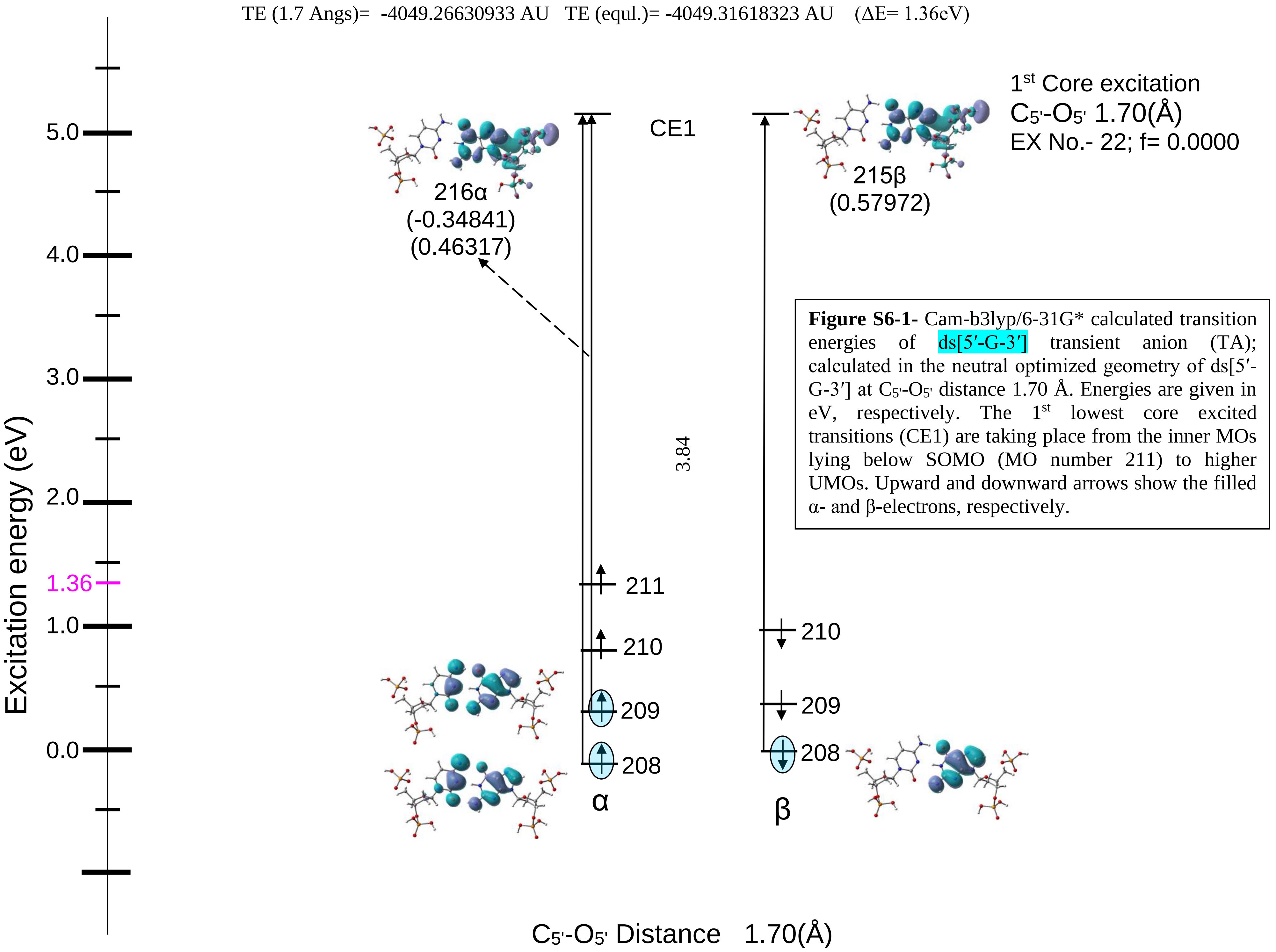

Figure S6-1- Cam-b3lyp/6-31G* calculated transition energies of ds[5′-G-3′] transient anion (TA); calculated in the neutral optimized geometry of ds[5′-G-3′] at $C_{5'}$-$O_{5'}$ distance 1.70 Å. Energies are given in eV, respectively. The 1st lowest core excited transitions (CE1) are taking place from the inner MOs lying below SOMO (MO number 211) to higher UMOs. Upward and downward arrows show the filled α- and β-electrons, respectively.

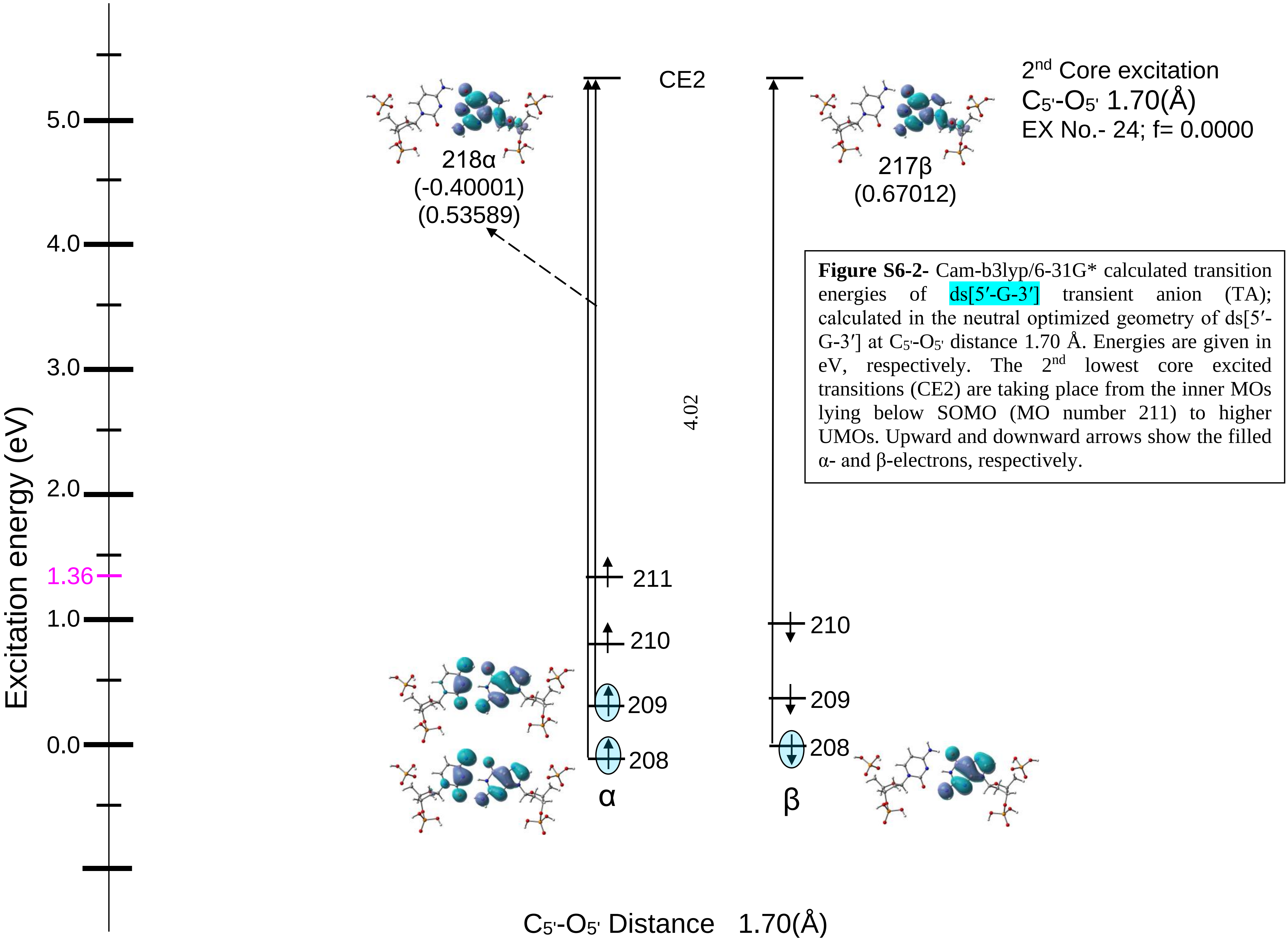

Figure S6-2- Cam-b3lyp/6-31G* calculated transition energies of ds[5′-G-3′] transient anion (TA); calculated in the neutral optimized geometry of ds[5′-G-3′] at $C_{5'}$-$O_{5'}$ distance 1.70 Å. Energies are given in eV, respectively. The 2nd lowest core excited transitions (CE2) are taking place from the inner MOs lying below SOMO (MO number 211) to higher UMOs. Upward and downward arrows show the filled α- and β-electrons, respectively.

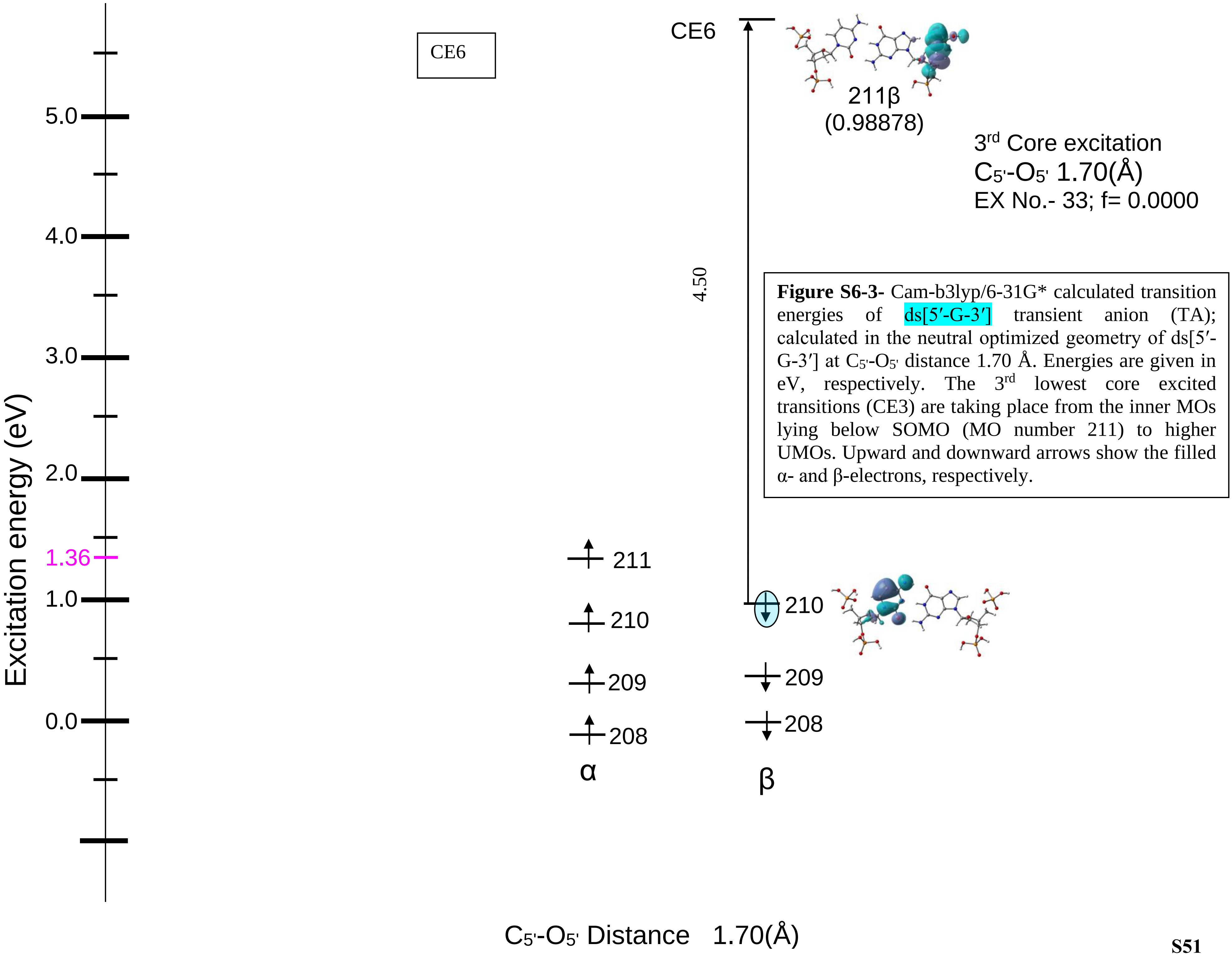

Figure S6-3- Cam-b3lyp/6-31G* calculated transition energies of ds[5′-G-3′] transient anion (TA); calculated in the neutral optimized geometry of ds[5′-G-3′] at $C_{5'}$-$O_{5'}$ distance 1.70 Å. Energies are given in eV, respectively. The 3rd lowest core excited transitions (CE3) are taking place from the inner MOs lying below SOMO (MO number 211) to higher UMOs. Upward and downward arrows show the filled α- and β-electrons, respectively.

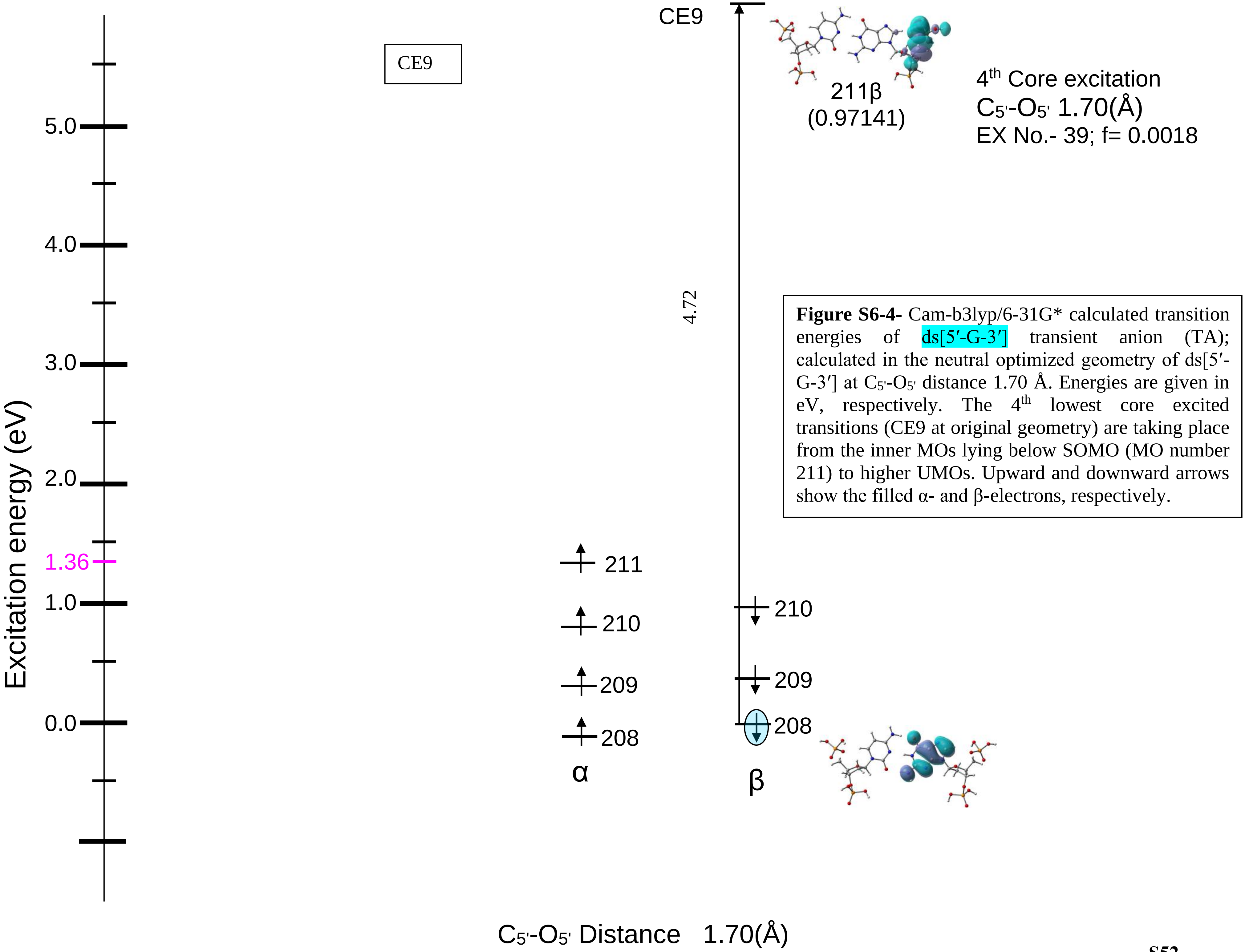

Figure S6-4- Cam-b3lyp/6-31G* calculated transition energies of ds[5′-G-3′] transient anion (TA); calculated in the neutral optimized geometry of ds[5′-G-3′] at $C_{5'}$-$O_{5'}$ distance 1.70 Å. Energies are given in eV, respectively. The 4th lowest core excited transitions (CE9 at original geometry) are taking place from the inner MOs lying below SOMO (MO number 211) to higher UMOs. Upward and downward arrows show the filled α- and β-electrons, respectively.

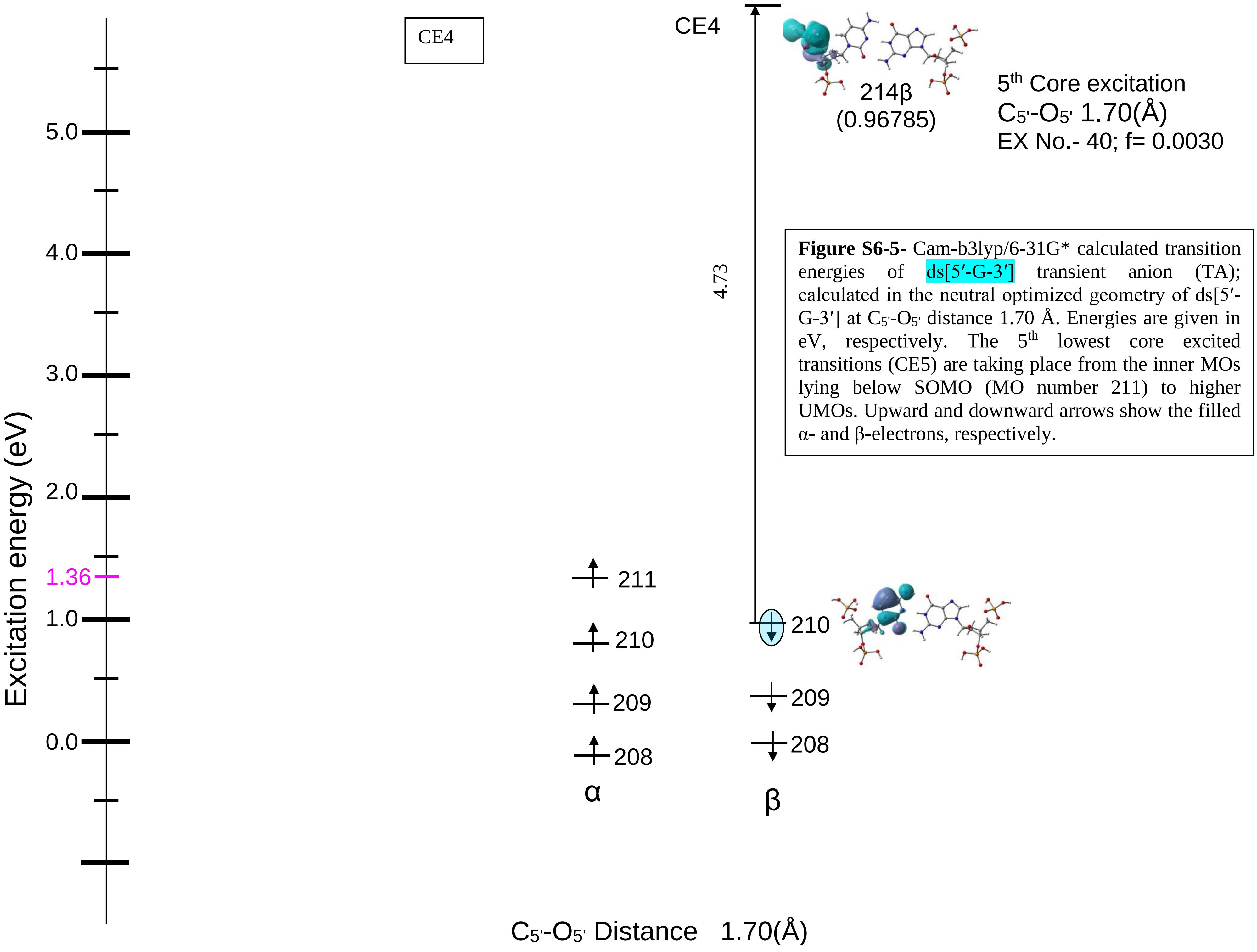

Figure S6-5- Cam-b3lyp/6-31G* calculated transition energies of ds[5′-G-3′] transient anion (TA); calculated in the neutral optimized geometry of ds[5′-G-3′] at $C_{5'}$-$O_{5'}$ distance 1.70 Å. Energies are given in eV, respectively. The 5th lowest core excited transitions (CE5) are taking place from the inner MOs lying below SOMO (MO number 211) to higher UMOs. Upward and downward arrows show the filled α- and β-electrons, respectively.

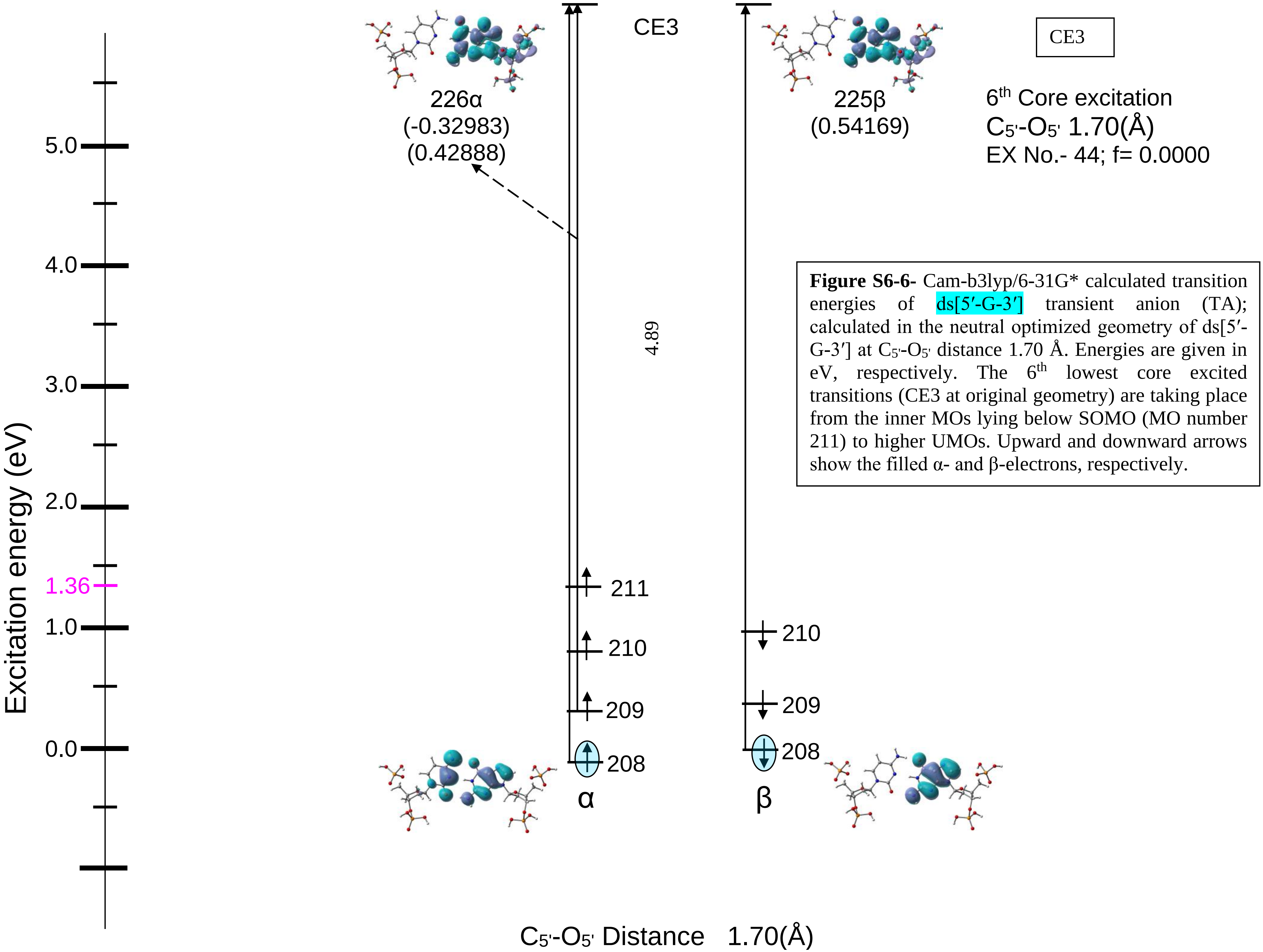

Figure S6-6- Cam-b3lyp/6-31G* calculated transition energies of ds[5′-G-3′] transient anion (TA); calculated in the neutral optimized geometry of ds[5′-G-3′] at $C_{5'}$-$O_{5'}$ distance 1.70 Å. Energies are given in eV, respectively. The 6th lowest core excited transitions (CE3 at original geometry) are taking place from the inner MOs lying below SOMO (MO number 211) to higher UMOs. Upward and downward arrows show the filled α- and β-electrons, respectively.

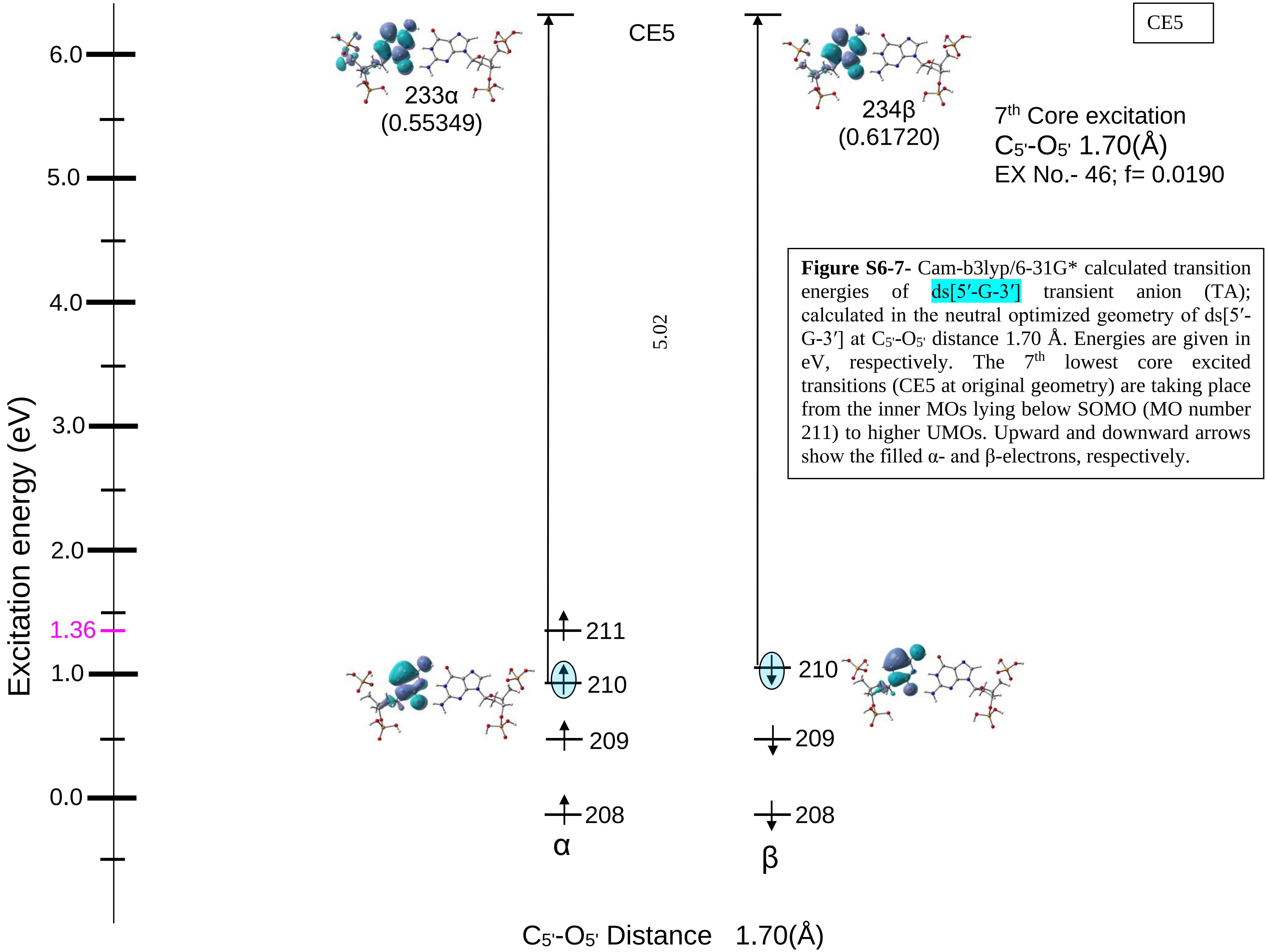

Figure S6-7- Cam-b3lyp/6-31G* calculated transition energies of ds[5′-G-3′] transient anion (TA); calculated in the neutral optimized geometry of ds[5′-G-3′] at $C_{5'}$-$O_{5'}$ distance 1.70 Å. Energies are given in eV, respectively. The 7th lowest core excited transitions (CE5 at original geometry) are taking place from the inner MOs lying below SOMO (MO number 211) to higher UMOs. Upward and downward arrows show the filled α- and β-electrons, respectively.

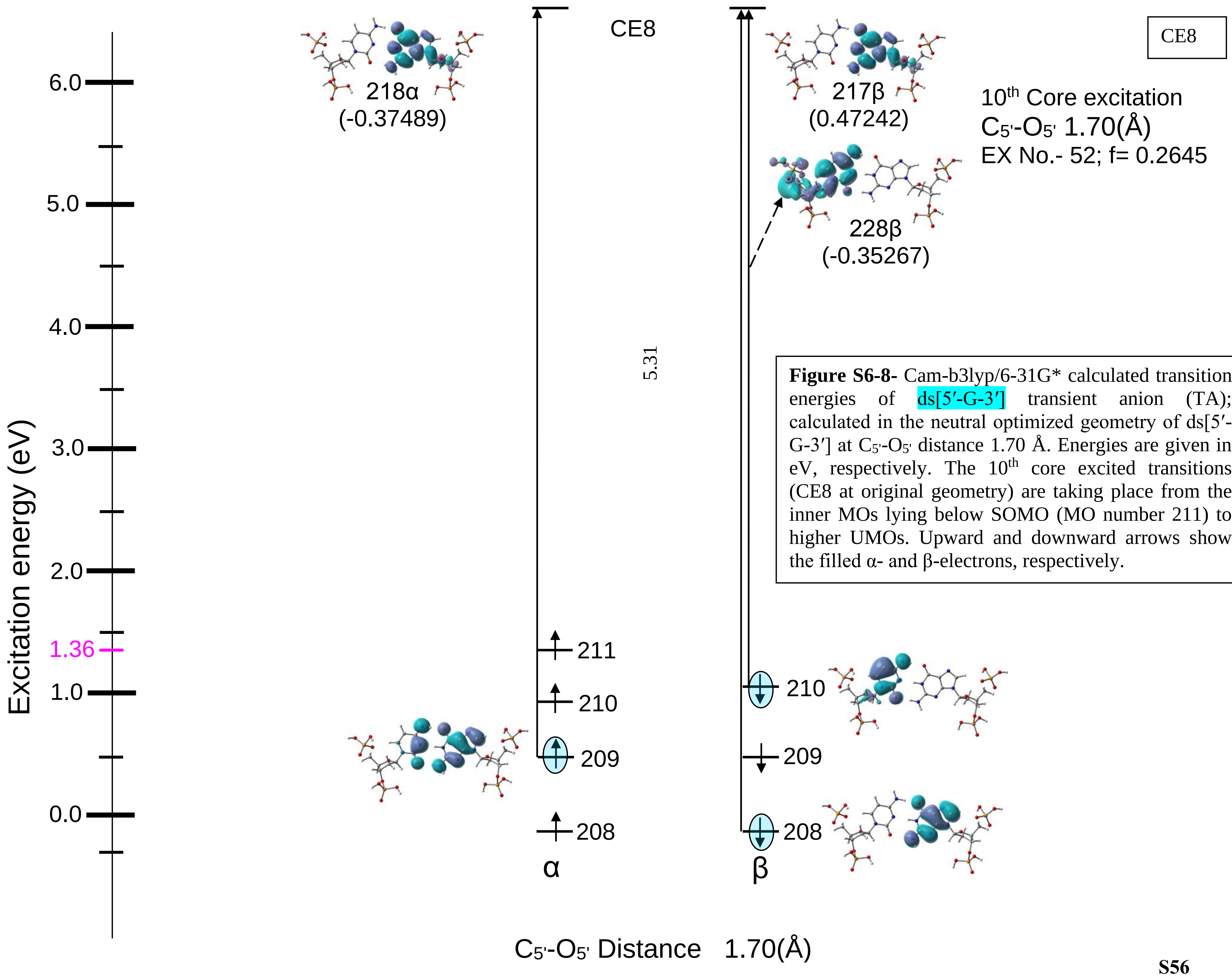

Figure S6-8- Cam-b3lyp/6-31G* calculated transition energies of ds[5′-G-3′] transient anion (TA); calculated in the neutral optimized geometry of ds[5′-G-3′] at $C_{5'}$-$O_{5'}$ distance 1.70 Å. Energies are given in eV, respectively. The 10th core excited transitions (CE8 at original geometry) are taking place from the inner MOs lying below SOMO (MO number 211) to higher UMOs. Upward and downward arrows show the filled α- and β-electrons, respectively.

Supporting Information 7

Transition energies with molecular orbitals (MOs) due to eight lowest core excited shape resonances (CE1 – CE8). Transition energies in eV are calculated at $C_{5'}$-$O_{5'}$ distance 1.90 Å

Figure S7-1- Cam-b3lyp/6-31G* calculated transition energies of ds[5′-G-3′] transient anion (TA); calculated in the neutral optimized geometry of ds[5′-G-3′] at $C_{5'}$-$O_{5'}$ distance 1.90 Å. Energies are given in eV, respectively. The 1st lowest core excited transitions (CE9 in original structure) are taking place from the inner MOs lying below SOMO (MO number 211 α) to higher UMOs. Upward and downward arrows show the filled α- and β-electrons, respectively. (CE9)

Figure S7-2- Cam-b3lyp/6-31G* calculated transition energies of ds[5′-G-3′] transient anion (TA); calculated in the neutral optimized geometry of ds[5′-G-3′] at $C_{5'}$-$O_{5'}$ distance 1.90 Å. Energies are given in eV, respectively. The 3rd lowest core excited transitions (CE2 in original structure) are taking place from the inner MOs lying below SOMO (MO number 211) to higher UMOs. Upward and downward arrows show the filled α- and β-electrons, respectively. (CE2)

Figure S7-3- Cam-b3lyp/6-31G* calculated transition energies of ds[5′-G-3′] transient anion (TA); calculated in the neutral optimized geometry of ds[5′-G-3′] at $C_{5'}$-$O_{5'}$ distance 1.90 Å. Energies are given in eV, respectively. The 4th lowest core excited transitions (CE1 in original structure) are taking place from the inner MOs lying below SOMO (MO number 211) to higher UMOs. Upward and downward arrows show the filled α- and β-electrons, respectively. (CE1)

Figure S7-4- Cam-b3lyp/6-31G* calculated transition energies of ds[5′-G-3′] transient anion (TA); calculated in the neutral optimized geometry of ds[5′-G-3′] at $C_{5'}$-$O_{5'}$ distance 1.90 Å. Energies are given in eV, respectively. The 5th lowest core excited transitions (CE9 in original structure) are taking place from the inner MOs lying below SOMO (MO number 211 β) to higher UMOs. Upward and downward arrows show the filled α- and β-electrons, respectively. (CE9)

Figure S7-5- Cam-b3lyp/6-31G* calculated transition energies of ds[5′-G-3′] transient anion (TA); calculated in the neutral optimized geometry of ds[5′-G-3′] at $C_{5'}$-$O_{5'}$ distance 1.90 Å. Energies are given in eV, respectively. The 6th lowest core excited transitions (also CE6 in original structure) are taking place from the inner MOs lying below SOMO (MO number 211) to higher UMOs. Upward and downward arrows show the filled α- and β-electrons, respectively. (CE6)

Figure S7-6- Cam-b3lyp/6-31G* calculated transition energies of ds[5′-G-3′] transient anion (TA); calculated in the neutral optimized geometry of ds[5′-G-3′] at $C_{5'}$-$O_{5'}$ distance 1.90 Å. Energies are given in eV, respectively. The 7th lowest core excited transitions (CE3 in the original structure) are taking place from the inner MOs lying below SOMO (MO number 211) to higher UMOs. Upward and downward arrows show the filled α- and β-electrons, respectively. (CE3)

Figure S7-7- Cam-b3lyp/6-31G* calculated transition energies of ds[5′-G-3′] transient anion (TA); calculated in the neutral optimized geometry of ds[5′-G-3′] at $C_{5'}$-$O_{5'}$ distance 1.90 Å. Energies are given in eV, respectively. The 8th lowest core excited transitions (CE5 in the original structure) are taking place from the inner MOs lying below SOMO (MO number 211) to higher UMOs. Upward and downward arrows show the filled α- and β-electrons, respectively. (CE5)

Figure S7-8- Cam-b3lyp/6-31G* calculated transition energies of ds[5′-G-3′] transient anion (TA); calculated in the neutral optimized geometry of ds[5′-G-3′] at $C_{5'}$-$O_{5'}$ distance 1.90 Å. Energies are given in eV, respectively. The 9th lowest core excited transitions (CE8 in the original structure) are taking place from the inner MOs lying below SOMO (MO number 211) to higher UMOs. Upward and downward arrows show the filled α- and β-electrons, respectively. (CE8)

TE (1.9 Angs)= -4049.25259113 AU TE (equl.)= -4049.31618323 AU (ΔE= 1.73 eV)

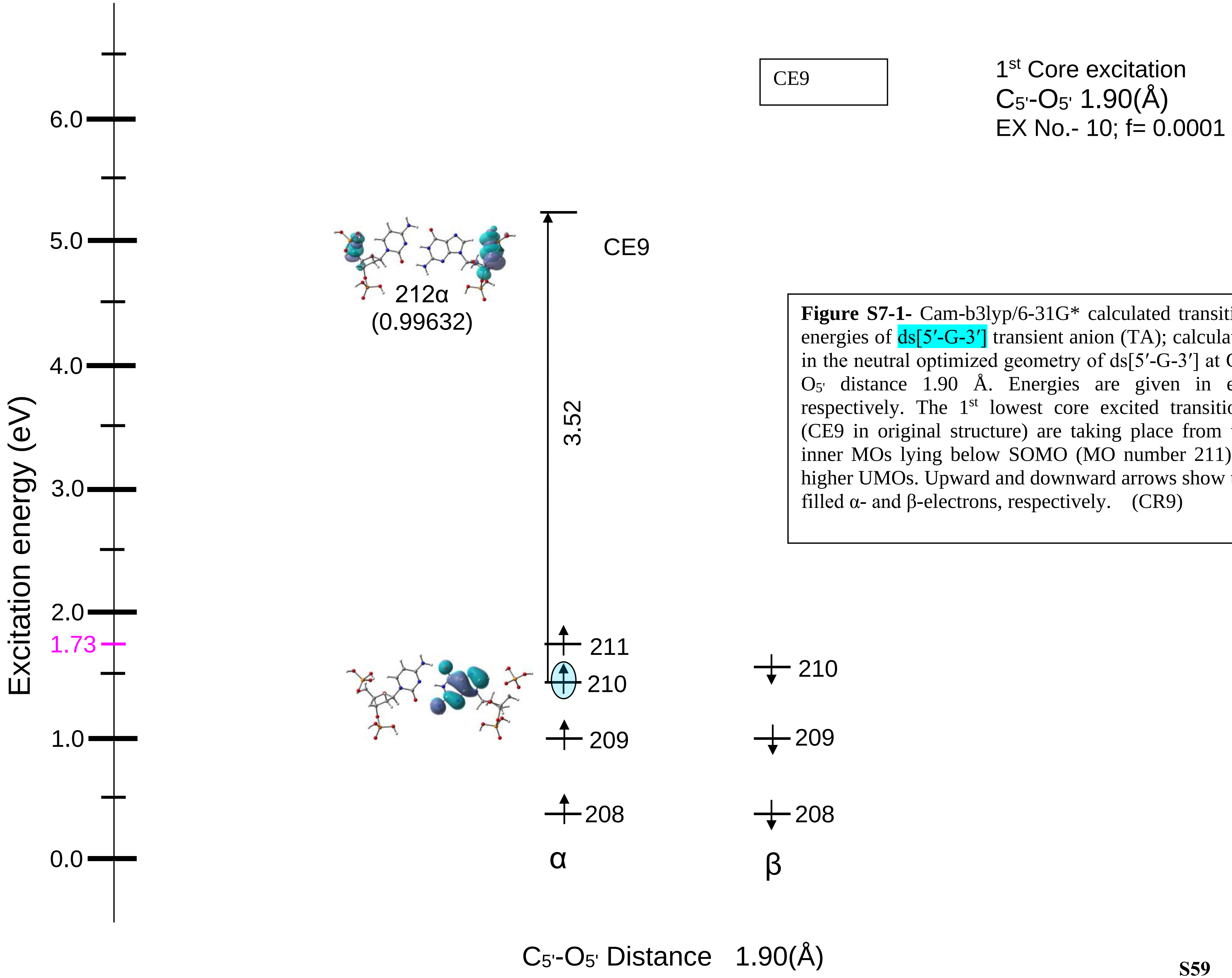

Figure S7-1- Cam-b3lyp/6-31G* calculated transition energies of ds[5′-G-3′] transient anion (TA); calculated in the neutral optimized geometry of ds[5′-G-3′] at $C_{5'}$-$O_{5'}$ distance 1.90 Å. Energies are given in eV, respectively. The 1st lowest core excited transitions (CE9 in original structure) are taking place from the inner MOs lying below SOMO (MO number 211) to higher UMOs. Upward and downward arrows show the filled α- and β-electrons, respectively. (CR9)

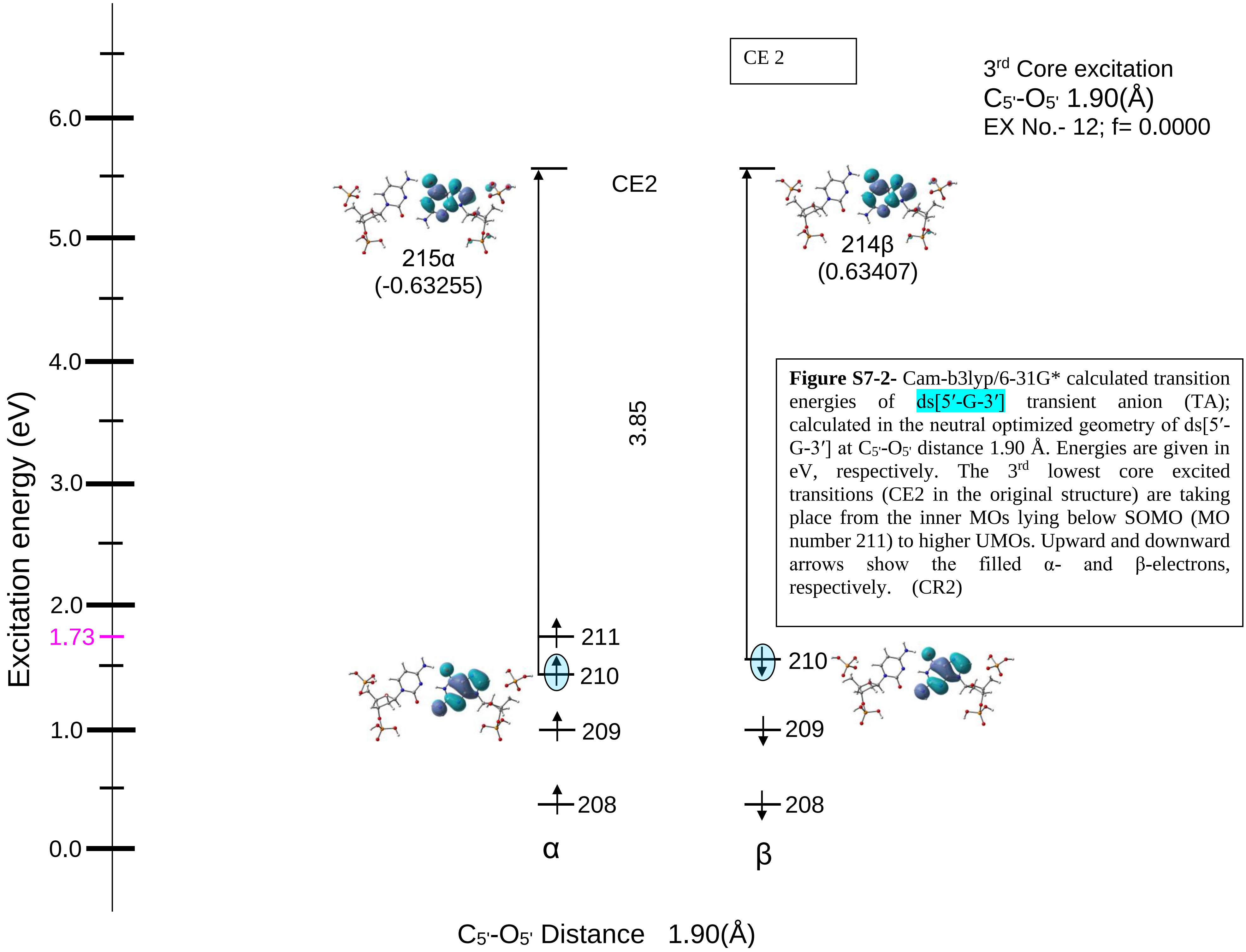

Figure S7-2- Cam-b3lyp/6-31G* calculated transition energies of ds[5′-G-3′] transient anion (TA); calculated in the neutral optimized geometry of ds[5′-G-3′] at $C_{5'}$-$O_{5'}$ distance 1.90 Å. Energies are given in eV, respectively. The 3rd lowest core excited transitions (CE2 in the original structure) are taking place from the inner MOs lying below SOMO (MO number 211) to higher UMOs. Upward and downward arrows show the filled α- and β-electrons, respectively. (CR2)

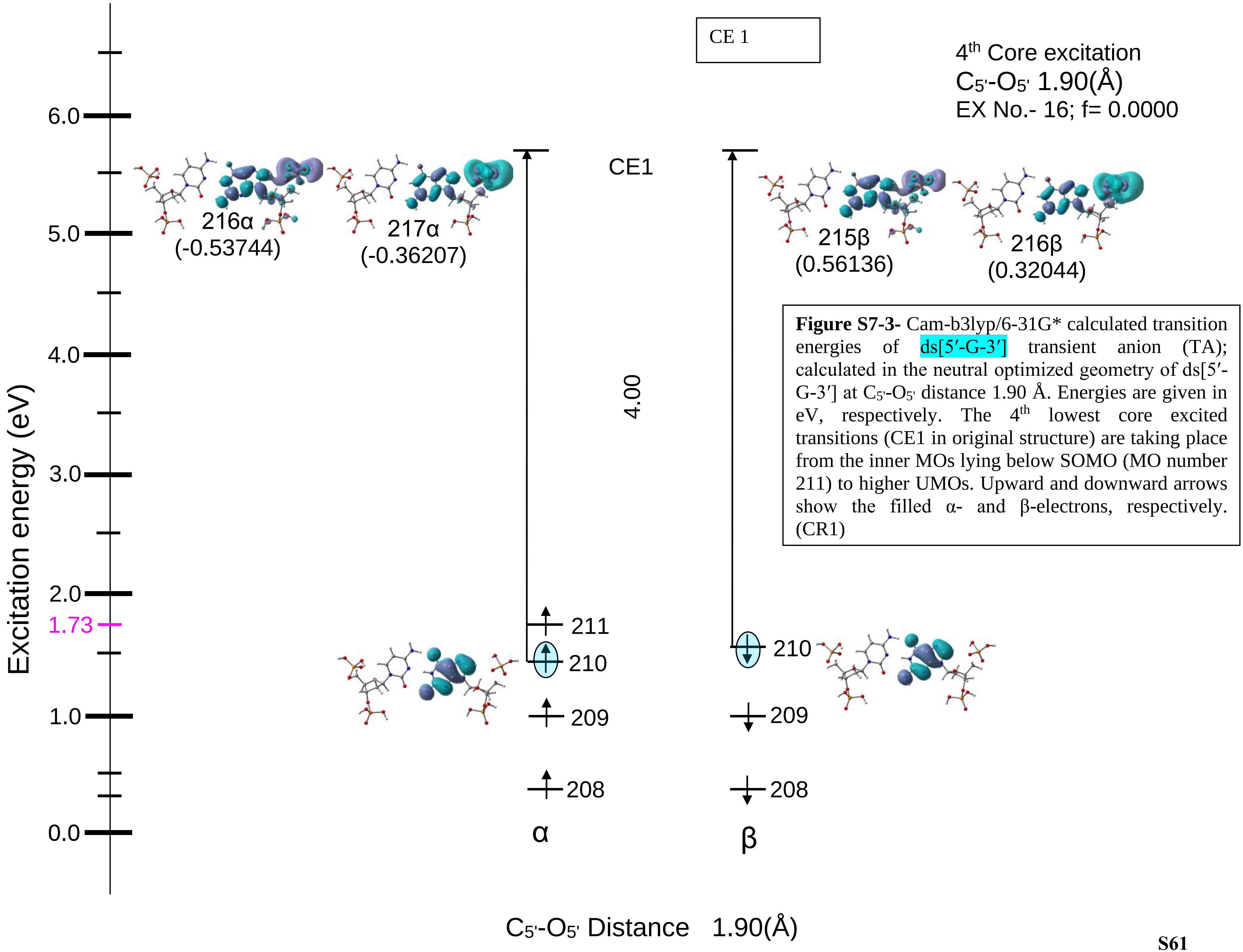

Figure S7-3- Cam-b3lyp/6-31G* calculated transition energies of ds[5′-G-3′] transient anion (TA); calculated in the neutral optimized geometry of ds[5′-G-3′] at $C_{5'}$-$O_{5'}$ distance 1.90 Å. Energies are given in eV, respectively. The 4th lowest core excited transitions (CE1 in original structure) are taking place from the inner MOs lying below SOMO (MO number 211) to higher UMOs. Upward and downward arrows show the filled α- and β-electrons, respectively. (CR1)

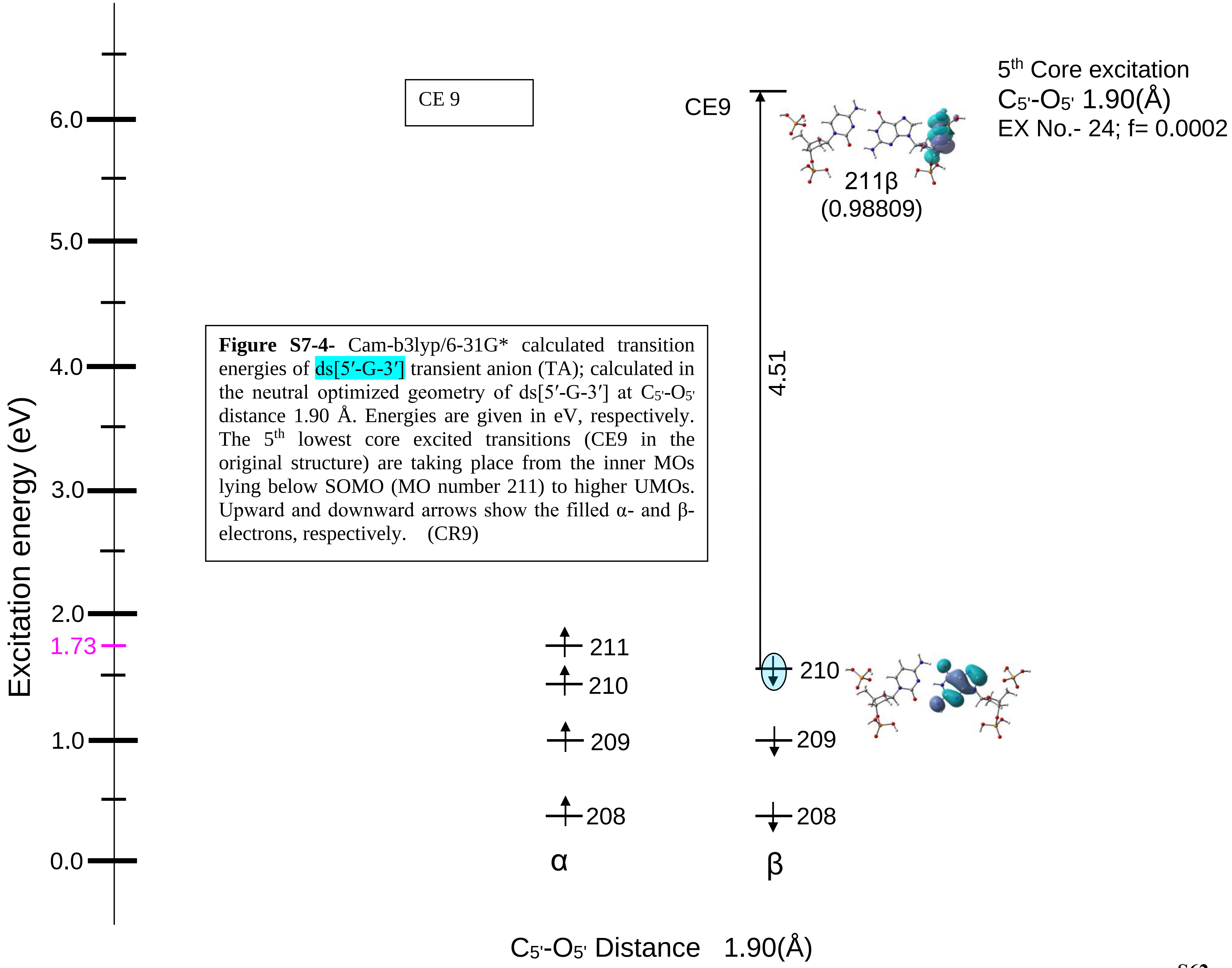

Figure S7-4- Cam-b3lyp/6-31G* calculated transition energies of ds[5′-G-3′] transient anion (TA); calculated in the neutral optimized geometry of ds[5′-G-3′] at $C_{5'}$-$O_{5'}$ distance 1.90 Å. Energies are given in eV, respectively. The 5th lowest core excited transitions (CE9 in the original structure) are taking place from the inner MOs lying below SOMO (MO number 211) to higher UMOs. Upward and downward arrows show the filled α- and β-electrons, respectively. (CR9)

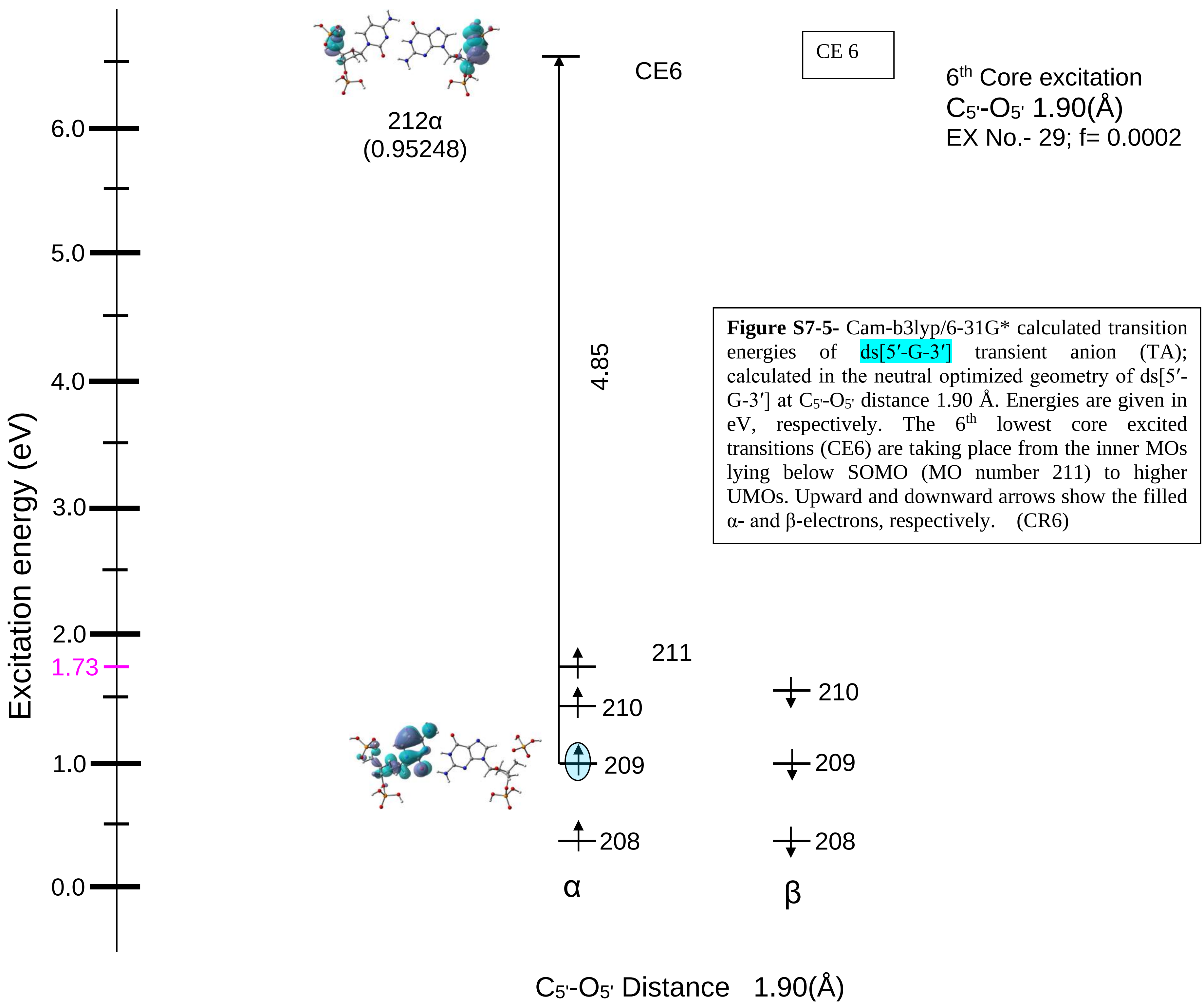

Figure S7-5- Cam-b3lyp/6-31G* calculated transition energies of ds[5′-G-3′] transient anion (TA); calculated in the neutral optimized geometry of ds[5′-G-3′] at $C_{5'}$-$O_{5'}$ distance 1.90 Å. Energies are given in eV, respectively. The 6th lowest core excited transitions (CE6) are taking place from the inner MOs lying below SOMO (MO number 211) to higher UMOs. Upward and downward arrows show the filled α- and β-electrons, respectively. (CR6)

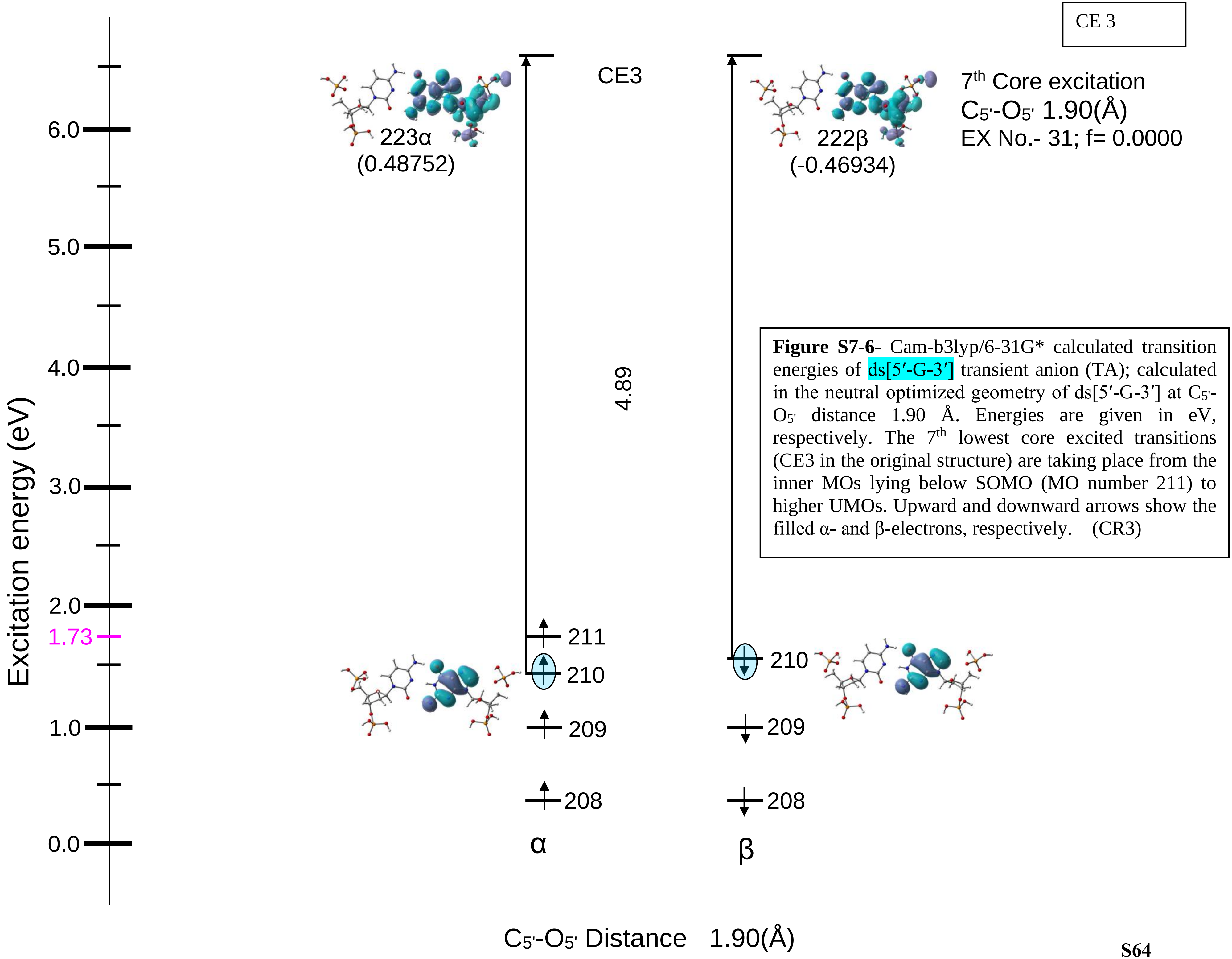

Figure S7-6- Cam-b3lyp/6-31G* calculated transition energies of ds[5′-G-3′] transient anion (TA); calculated in the neutral optimized geometry of ds[5′-G-3′] at $C_{5'}$-$O_{5'}$ distance 1.90 Å. Energies are given in eV, respectively. The 7th lowest core excited transitions (CE3 in the original structure) are taking place from the inner MOs lying below SOMO (MO number 211) to higher UMOs. Upward and downward arrows show the filled α- and β-electrons, respectively. (CR3)

CE 5

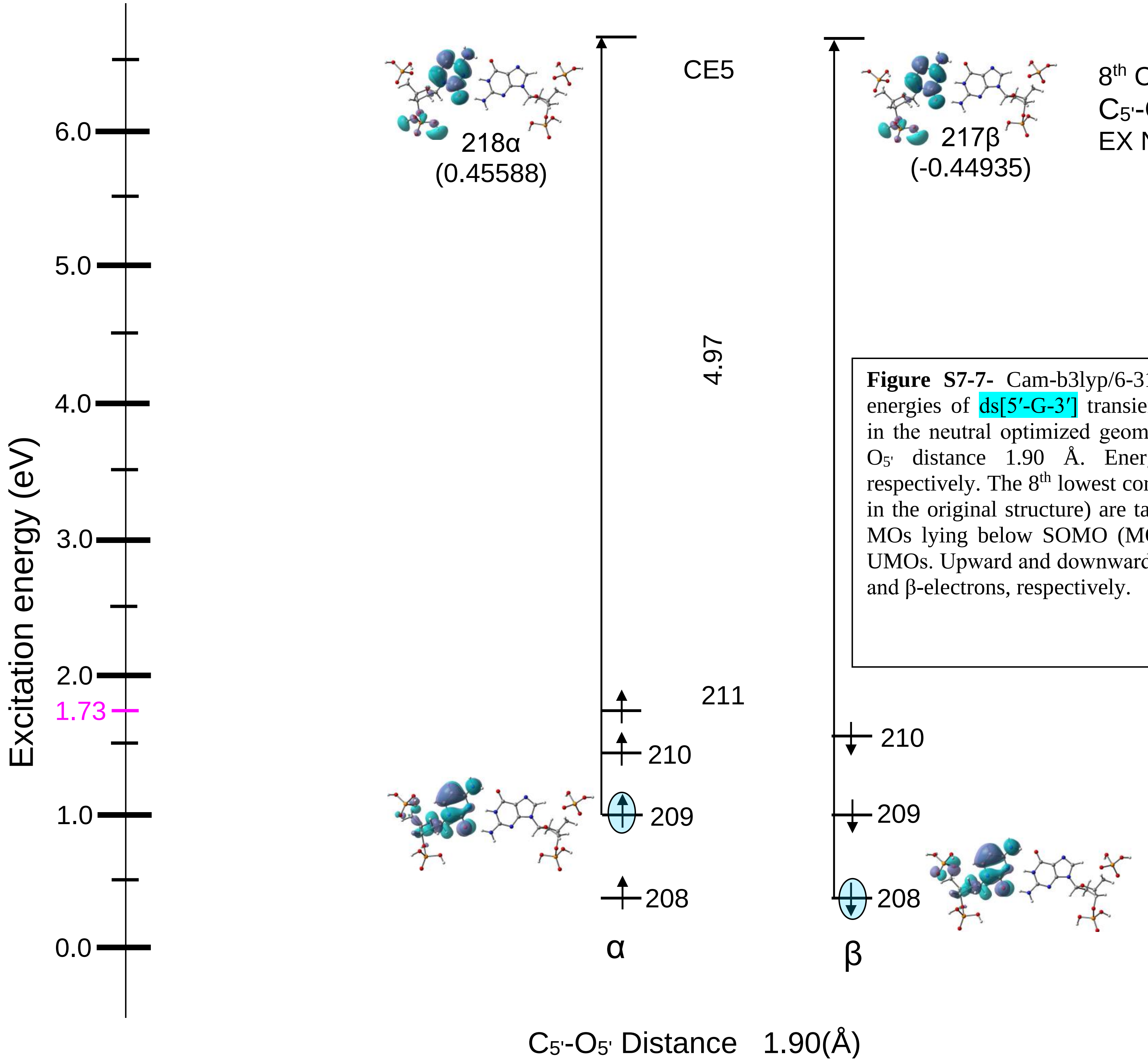

Figure S7-7- Cam-b3lyp/6-31G* calculated transition energies of ds[5′-G-3′] transient anion (TA); calculated in the neutral optimized geometry of ds[5′-G-3′] at $C_{5'}$-$O_{5'}$ distance 1.90 Å. Energies are given in eV, respectively. The 8th lowest core excited transitions (CE5 in the original structure) are taking place from the inner MOs lying below SOMO (MO number 211) to higher UMOs. Upward and downward arrows show the filled α- and β-electrons, respectively. (CR5)

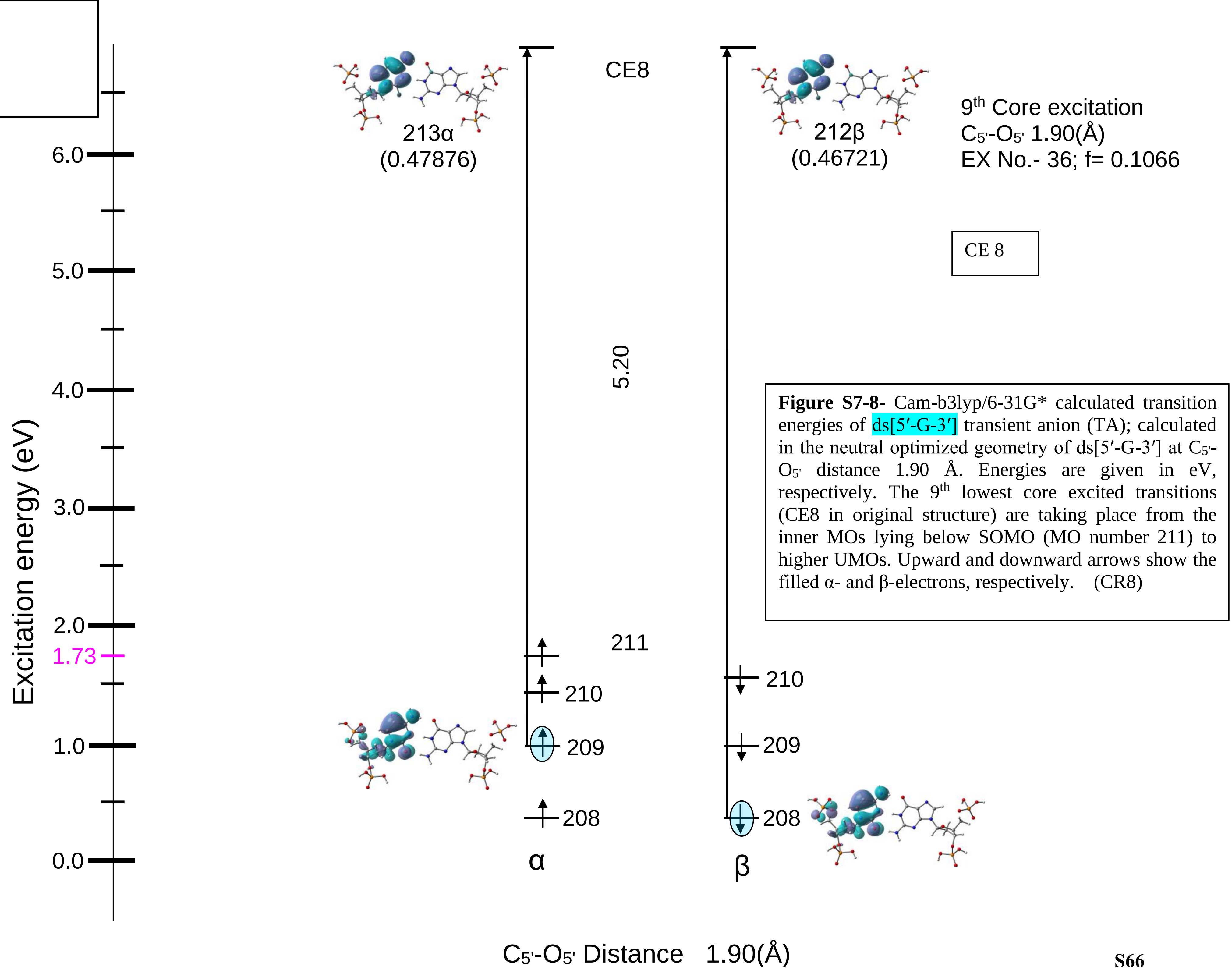

Figure S7-8- Cam-b3lyp/6-31G* calculated transition energies of ds[5′-G-3′] transient anion (TA); calculated in the neutral optimized geometry of ds[5′-G-3′] at $C_{5'}$-$O_{5'}$ distance 1.90 Å. Energies are given in eV, respectively. The 9th lowest core excited transitions (CE8 in original structure) are taking place from the inner MOs lying below SOMO (MO number 211) to higher UMOs. Upward and downward arrows show the filled α- and β-electrons, respectively. (CR8)

Supporting Information 8

Transition energies with molecular orbitals (MOs) due to three lowest core excited shape resonances (CE1 – CE9). Transition energies in eV are calculated at $C_{5'}$-$O_{5'}$ distance 2.10 Å

Figure S8-1- Cam-b3lyp/6-31G* calculated transition energies of ds[5′-G-3′] transient anion (TA); calculated in the neutral optimized geometry of ds[5′-G-3′] at $C_{5'}$-$O_{5'}$ distance 2.10 Å. Energies are given in eV, respectively. The 1st lowest core excited transitions (CE9 in original structure) are taking place from the inner MOs lying below SOMO (MO number 211) to higher UMOs. Upward and downward arrows show the filled α- and β-electrons, respectively. (CE9)

Figure S8-2- Cam-b3lyp/6-31G* calculated transition energies of ds[5′-G-3′] transient anion (TA); calculated in the neutral optimized geometry of ds[5′-G-3′] at $C_{5'}$-$O_{5'}$ distance 2.10 Å. Energies are given in eV, respectively. The 3rd lowest core excited transitions (CE9 in original structure) are taking place from the inner MOs lying below SOMO (MO number 211) to higher UMOs. Upward and downward arrows show the filled α- and β-electrons, respectively. (CE9)

Figure S8-3- Cam-b3lyp/6-31G* calculated transition energies of ds[5′-G-3′] transient anion (TA); calculated in the neutral optimized geometry of ds[5′-G-3′] at $C_{5'}$-$O_{5'}$ distance 2.10 Å. Energies are given in eV, respectively. The 4th lowest core excited transitions (CE6 in the original structure) are taking place from the inner MOs lying below SOMO (MO number 211) to higher UMOs. Upward and downward arrows show the filled α- and β-electrons, respectively. (CE6)

TE (2.1 Angs)= -4049.23614149 AU TE (equl.)= -4049.31618323 AU (ΔE= 2.18 eV)

1st Core excitation
$C_{5'}$-$O_{5'}$ 2.10 (Å)
EX No.- 2; f= 0.0000

CE9

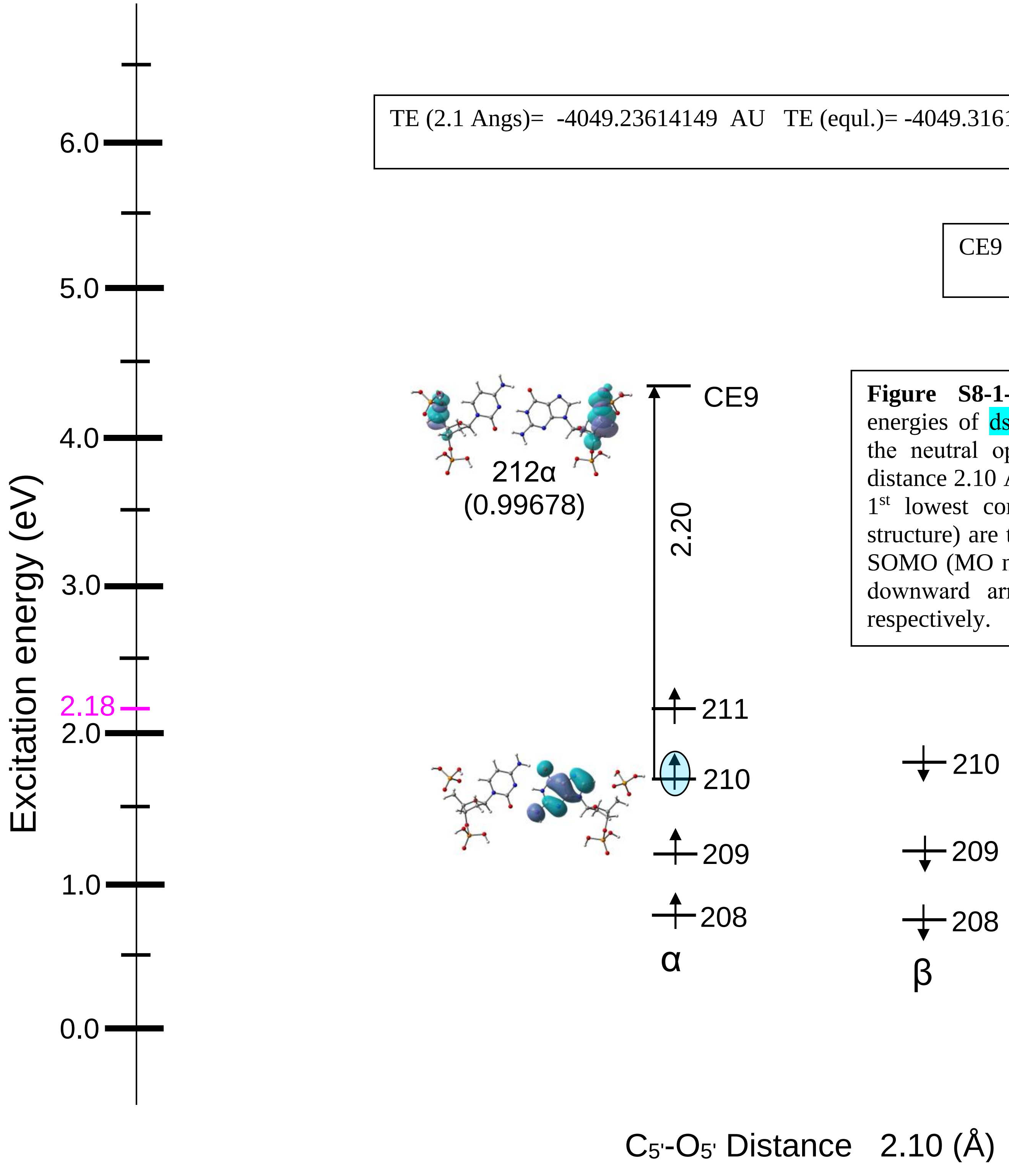

Figure S8-1- Cam-b3lyp/6-31G* calculated transition energies of ds[5′-G-3′] transient anion (TA); calculated in the neutral optimized geometry of ds[5′-G-3′] at $C_{5'}$-$O_{5'}$ distance 2.10 Å. Energies are given in eV, respectively. The 1st lowest core excited transitions (CE9 in the original structure) are taking place from the inner MOs lying below SOMO (MO number 211 α) to higher UMOs. Upward and downward arrows show the filled α- and β-electrons, respectively. (CR9)

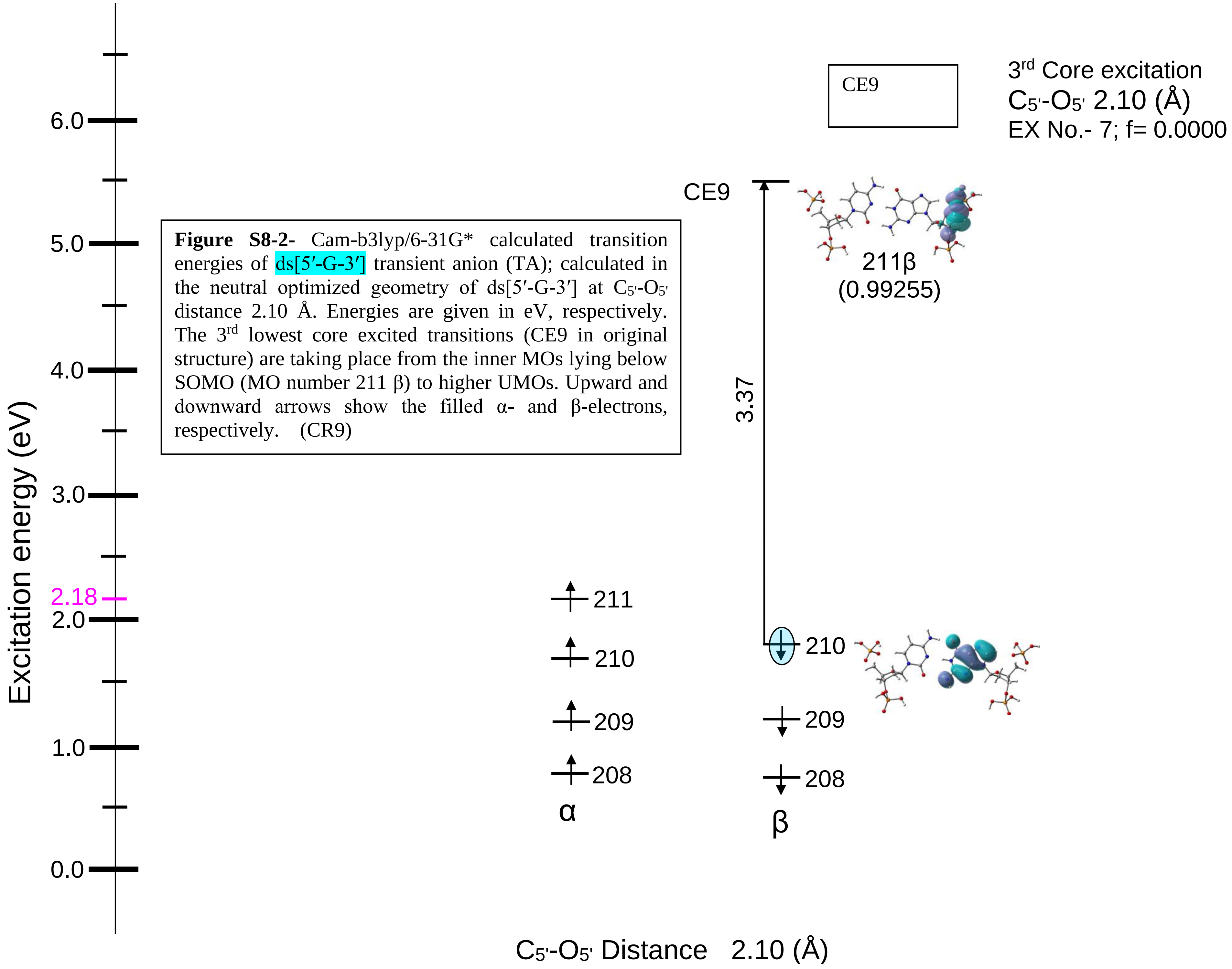

Figure S8-2- Cam-b3lyp/6-31G* calculated transition energies of ds[5′-G-3′] transient anion (TA); calculated in the neutral optimized geometry of ds[5′-G-3′] at $C_{5'}$-$O_{5'}$ distance 2.10 Å. Energies are given in eV, respectively. The 3rd lowest core excited transitions (CE9 in original structure) are taking place from the inner MOs lying below SOMO (MO number 211 β) to higher UMOs. Upward and downward arrows show the filled α- and β-electrons, respectively. (CR9)

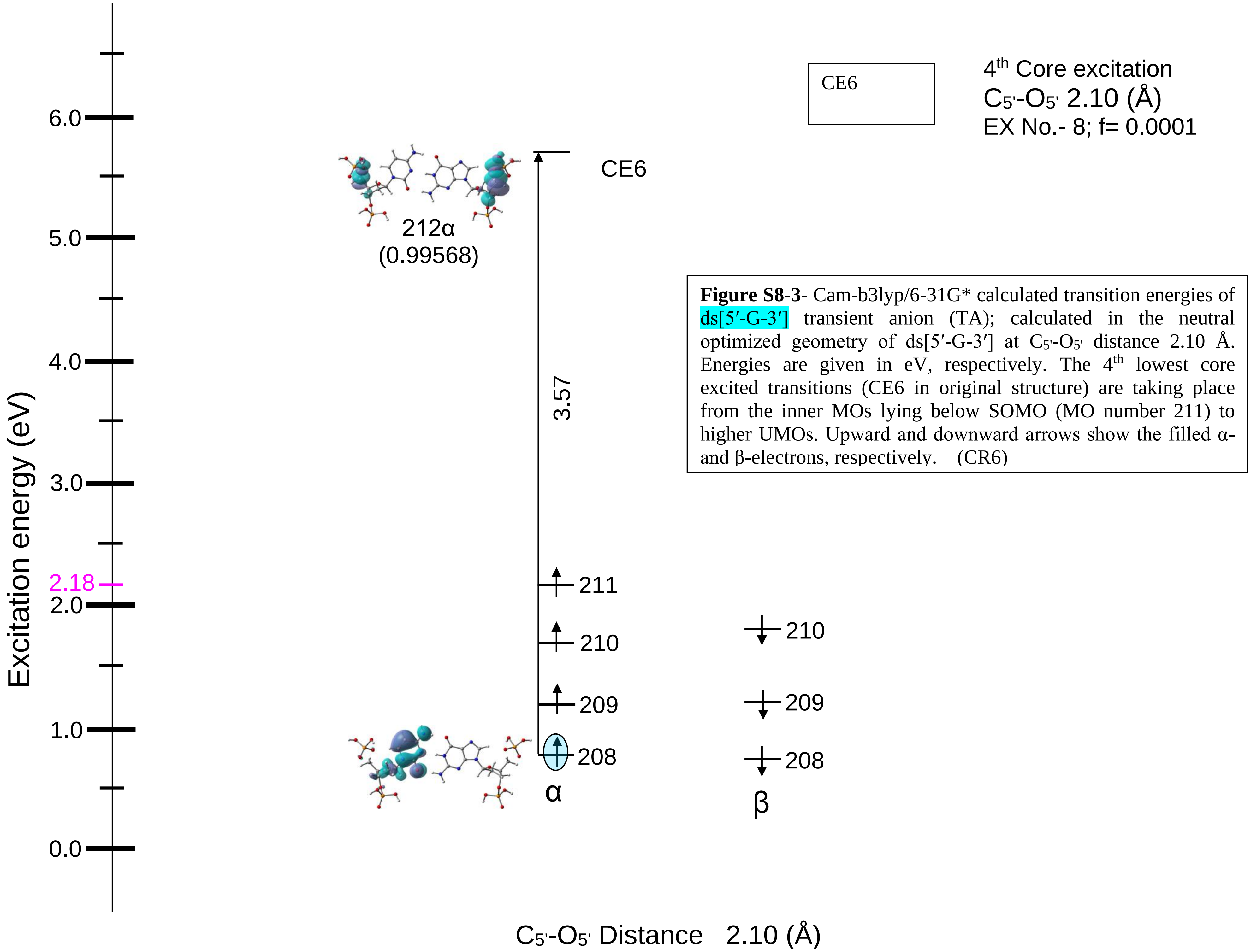

Figure S8-3- Cam-b3lyp/6-31G* calculated transition energies of ds[5′-G-3′] transient anion (TA); calculated in the neutral optimized geometry of ds[5′-G-3′] at $C_{5'}$-$O_{5'}$ distance 2.10 Å. Energies are given in eV, respectively. The 4th lowest core excited transitions (CE6 in original structure) are taking place from the inner MOs lying below SOMO (MO number 211) to higher UMOs. Upward and downward arrows show the filled α- and β-electrons, respectively. (CR6)
